# LiCl-promoted amination of β-methoxy amides (γ-lactones)†

**DOI:** 10.1039/d0ra07170f

**Published:** 2020-09-21

**Authors:** Ru Zhao, Bing-Lin Zeng, Wen-Qiang Jia, Hong-Yi Zhao, Long-Ying Shen, Xiao-Jian Wang, Xian-Dao Pan

**Affiliations:** State Key Laboratory of Bioactive Substances and Functions of Natural Medicines, Institute of Materia Medica, Peking Union Medical College and Chinese Academy of Medical Sciences Beijing 100050 China xdp@imm.ac.cn; School of Pharmacy, Anhui University of Chinese Medicine Hefei 230012 China

## Abstract

An efficient and mild method has been developed for the amination of β-methoxy amides (γ-lactones) including natural products michelolide, costunolide and parthenolide derivatives by using lithium chloride in good yields. This reaction is applicable to a wide range of substrates with good functional group tolerance. Mechanism studies show that the reactions undergo a LiCl promoted MeOH elimination from the substrates to form the corresponding α,β-unsaturated intermediates followed by the Michael addition of amines.

## Abstract 2

The amination of β-methoxy amides (γ-lactones) including natural products michelolide, costunolide and parthenolide derivatives were first developed by using lithium chloride.

## Graphical abstract

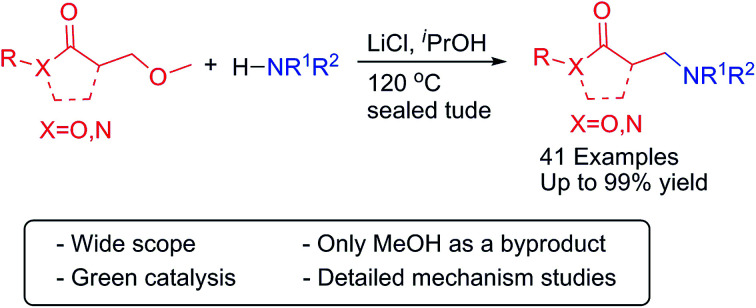

The formation of carbon–nitrogen bonds remains one of the most fundamental and widely practiced reactions in organic synthesis, due to the prevalence of this functionality in the preparations of functional molecules in pharmaceutical chemistry, biochemistry and material sciences.^1^ Various synthetic methodologies have been developed to form C(sp^2^)-N bonds, including the Goldberg reaction,^2^ Buchwald–Hartwig reaction,^3^ imine reduction^4^ and the nucleophilic addition of carbon-nucleophiles to imine derivatives.^5^ Meanwhile, the formations of C(sp^3^)-N bonds can be achieved by reductive amination, which involves the conversion of a carbonyl group to an amine *via* an imine intermediate, such as Eschweiler–Clarke reaction^6^ and Borch reductive amination.^7^ Nucleophilic substitution of alkyl(pseudo)halides with amines (amine alkylation) serves as one direct strategy for the preparation of alkylamines, while the necessity of pre-installation of the halogen atoms and the production of stoichiometric inorganic salt wastes are considered as two main drawbacks for its application in large scale industrial synthesis.^8^

Methoxy as the leaving group in the amination reactions has recently attracted the attention of organic chemists. For instance, Chiba and coworkers reported a method for the nucleophilic amination of methoxy arenes,^9^ which was achieved by using sodium hydride (NaH) in the presence of lithium iodide (LiI) through a concerted nucleophilic aromatic substitution pathway (Fig. 1a).^10^ Kondo and coworkers demonstrated that the organic superbase t-Bu-P4 efficiently catalyzes the amination of methoxy(hetero)arenes with the amine nucleophiles (Fig. 1b).^11^ The t-Bu-P4 is also suitable to catalyze the amination of β-(hetero)arylethyl ethers with amines to synthesize β-(hetero)arylethylamines (Fig. 1c).^12^ Sun and coworkers reported that C–S bond cleavage to access *N*-substituted acrylamide and β-aminopropanamide(Fig. 1d).^13^

**Fig. 1:**
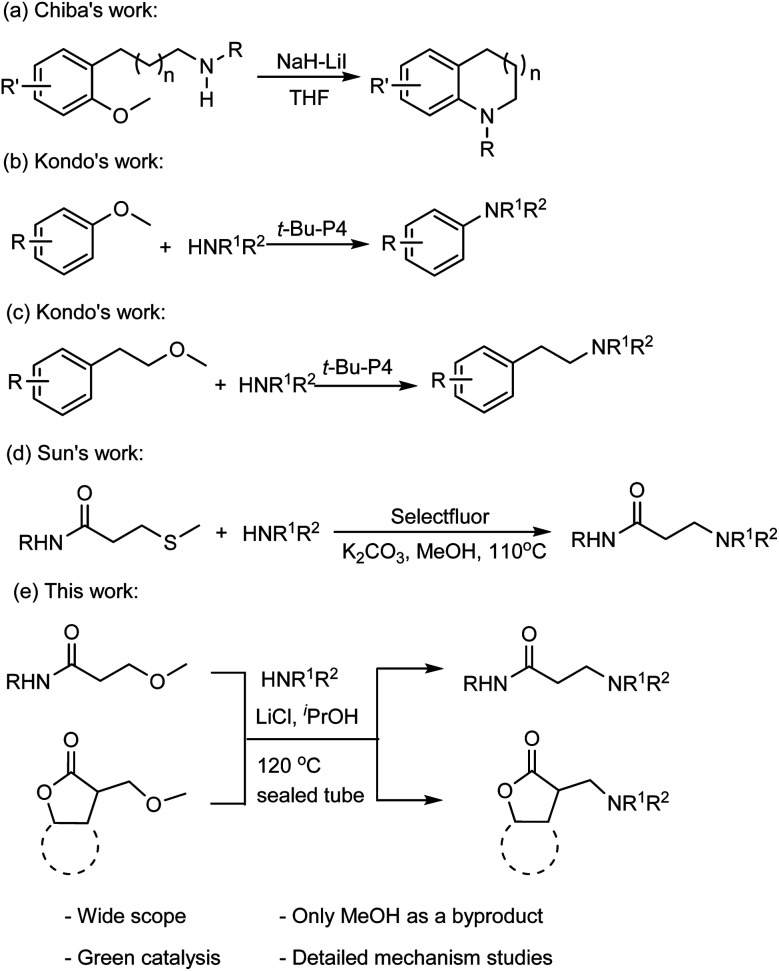
Amination reactions of methyl ethers.

Recently, we described the application of a CuBr–LiCl composite for the short-chain alkoxylation of aryl bromides.^14^ During that course of study, the single-shell lithium ion was found to embrace a unique affinity for oxygen and can be used as an additive to activate C–O bond and facilitate the nucleophilic reaction. On the basis of this study, we herein present the synthesis of β-amino amides (γ-lactones) *via* the elimination of methoxy group followed by Michael addition of an amine, that was promoted by LiCl in good yields under conventional conditions.

We initiated our study with the reaction of 3-methoxy-*N*-phenylpropanamide 1a and piperidine 2a in the presence of lithium salts (Table 1). It was found that the reaction performed in ^i^PrOH at 120 °C in a sealed tube proceeded smoothly in the presence of 2.0 equiv. of LiCl, giving the desired product 3aa in 70% yield (entry 1), while using other additives, including LiBr, LiI, LiOTf, Li_2_CO_3_ and NaCl instead, dramatically decreased the yields (entries 2–6). ^i^PrOH was proved to be a better solvent, whereas using other solvents, such as DMF or toluene, gave poor results (entries 7 and 8). Lowering the equivalent of LiCl to 1.0 equiv. reduced the yield of 3aa to 38% (entry 9). No conversion was observed when the reaction was performed in the absence of LiCl (entry 10). Moreover, reducing the reaction temperature or reaction time resulted in diminished yields (entries 11 and 12). Therefore, 1.0 equiv. of 3-methoxy propanamide, 2.0 equiv. of alkyl amine and 2.0 equiv. of LiCl in ^i^PrOH was chosen as the standard condition for the amination of 3-methoxy propanamides.

**Table 1:** Examination of the reaction conditions^a^

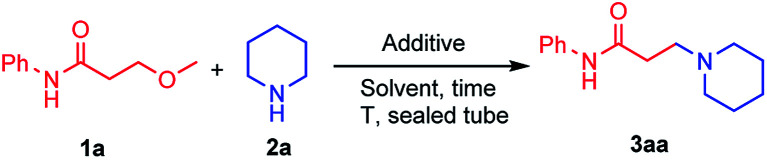					
Entry	Additive (equiv.)	Solvent	*T* (°C)	Time (h)	Yield^b^ (%)
1	LiCl (2.0)	^i^PrOH	120	12	70
2	LiBr (2.0)	^i^PrOH	120	12	30
3	LiI (2.0)	^i^PrOH	120	12	43
4	LiOTf (2.0)	^i^PrOH	120	12	38
5	Li_2_CO_3_ (2.0)	^i^PrOH	120	12	6
6	NaCl (2.0)	^i^PrOH	120	12	N. R.
7	LiCl (2.0)	DMF	120	12	46
8	LiCl (2.0)	Toluene	120	12	21
9	LiCl (1.0)	^i^PrOH	120	12	38
10^c^	—	^i^PrOH	120	12	N. R.
11	LiCl (2.0)	^i^PrOH	80	12	23
12	LiCl (2.0)	^i^PrOH	120	6	49

a Reaction conditions: 1a (0.45 mmol), 2a (0.90 mmol) and additive (2.0 equiv.) in solvent (3.0 mL) at 120 °C in sealed tube.

b Yield of isolated product.

c No LiCl was used.

With the optimized condition in hand, the substrate scope and functional group tolerance of the transformation was then examined (Scheme 1). It was found that the 3-methoxy-*N*-arylpropanamides without substitution or substituted with electron-donating (–OMe) or electron-withdrawing (–Cl, –Br) groups at the *para*-position of the *N*-aryl ring exhibit good tolerance under the present conditions, giving good yields of 70–77% (3aa–3da). Moreover, the diversity of amines was studied, including pyrrolidine, diethyl amine, dimethyl amine, morpholine and methyl amine solution, and the amination products were formed in moderate to good yields in all cases (3ab–3db, 3ac–3dc, 3ad–3dd, 3ae–3de, 3af). However, when using anilin (2g) as the starting material, no reaction took place. Replacement of the *N*-phenyl substituent with a benzyl group (1e) led to an increased yield of 83% (3ed). Remarkably, challenging 3-methoxypropanoyl piperazine derivatives also worked well under the optimized conditions, producing the desired products in good yields (3fa–3fe). Promoted by the successful amination of the amide, we then extended this transformation to β-methoxy γ-lactones. It was noteworthy to find that 3-methoxymethyl γ-lactones 4a also worked for this reaction with the high yield of 85% 5ad.

**Scheme 1:**
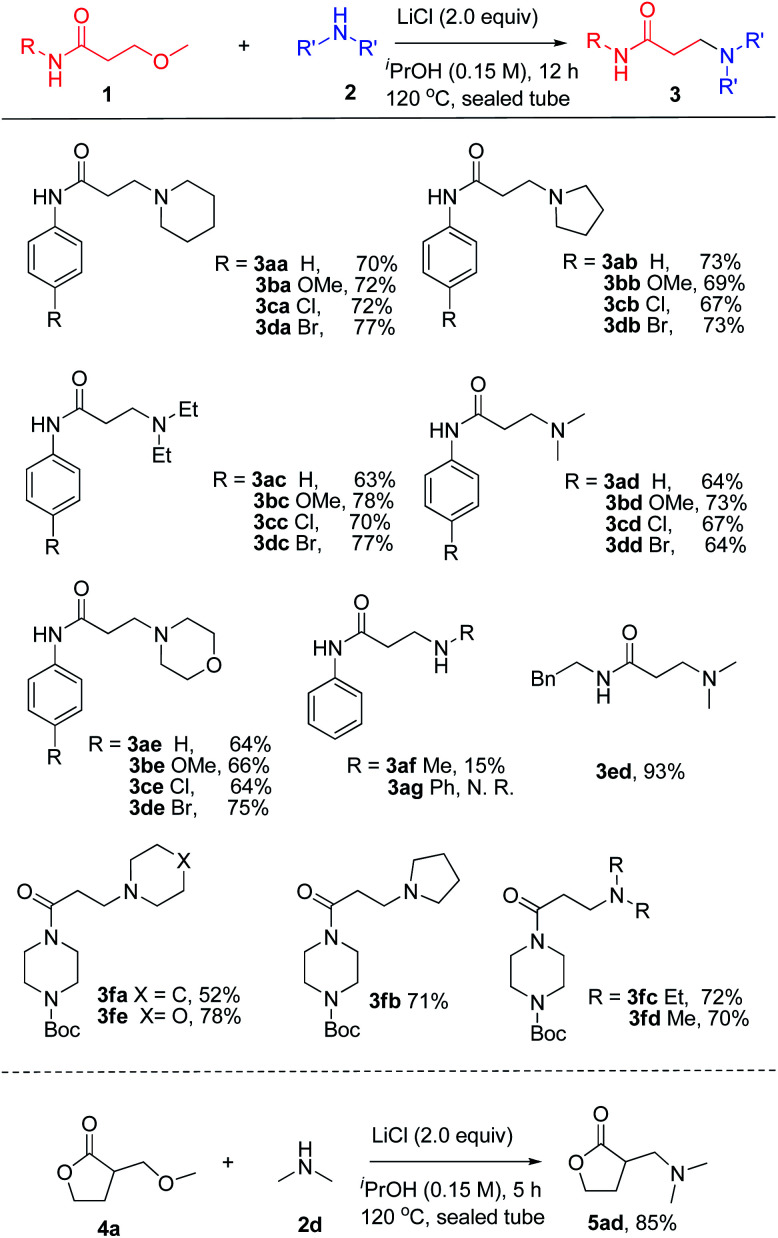
Evaluation of the substrate scope of β-methoxy amides and amines. ^*a*^Reactions were carried out with 1a (1.0 equiv.), 2a (2.0 equiv.) and LiCl (2.0 equiv.) in ^i^PrOH (0.15 M) at 120 °C for 12 h in sealed tube. Yields of isolated products are given.

Encouraged by the above results, our research was then extended to perform this transformation between the natural product michelolide derivatives 4b with β-methoxy γ-lactone subunit and various amines 2 (Scheme 2).^15^ Due to a high tolerance and compatibility of function groups, this strategy can be applied to 4b possessing both hydroxy group and carbon–carbon double bond. Both cyclic amines (2a, 2b, 2e, 2h) and linear amines (2d, 2i, 2f, 2j) gave the corresponding products in moderate to excellent yields. Additionally, the structure of product 5bb was unambiguously identified by X-ray crystallography.

**Scheme 2:**
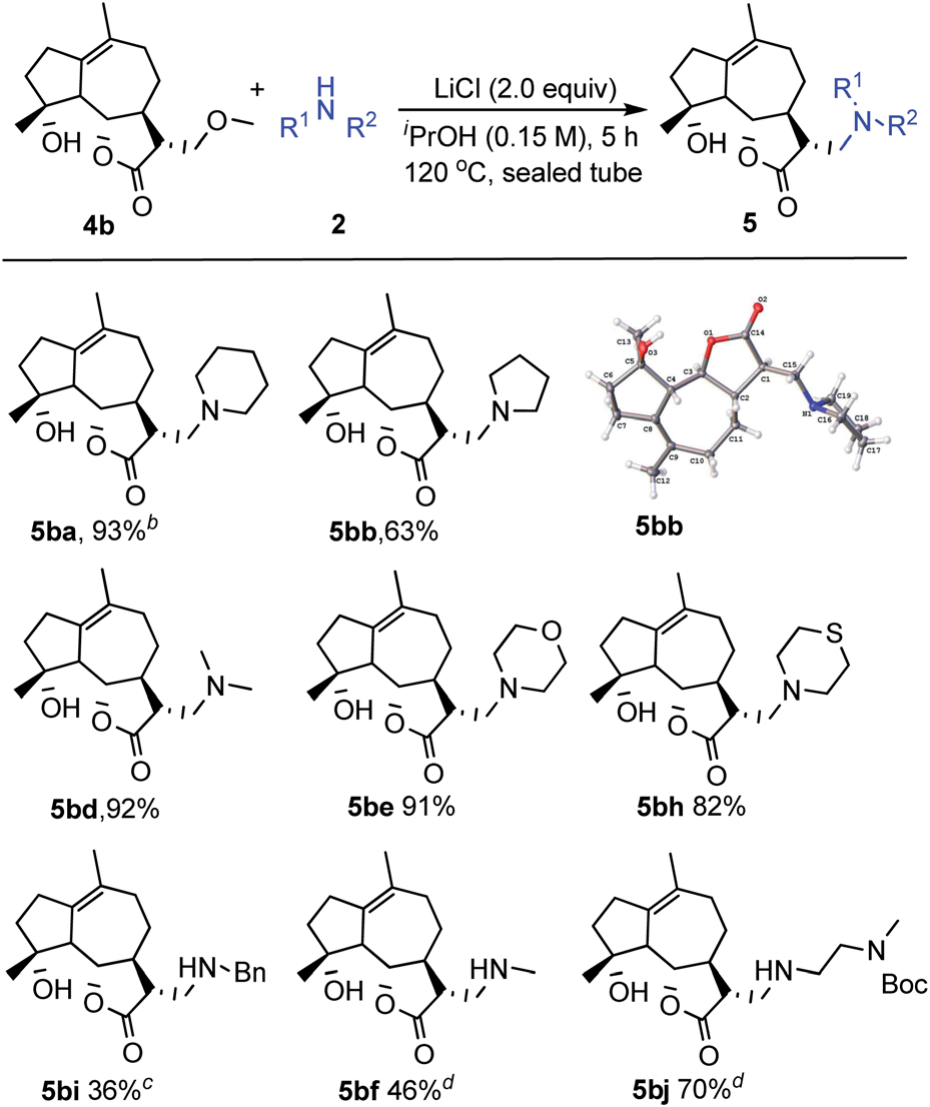
Evaluation of the substrate scope of amines with michelolide derivatives. ^*a*^Reactions were carried out with 4b (1.0 equiv.), 2 (2.0 equiv.) and LiCl (2.0 equiv.) in ^i^PrOH (0.15 M) at 120 °C for 5 h in sealed tube. Yields of isolated products are given. ^*b*^Reaction was conducted for 10 h. ^*c*^Reaction was conducted for 20 h. ^*d*^Reaction was conducted for 15 h.

Meanwhile, it is well demonstrated that amine substituted natural products is an efficient hydrophilic modification strategy used in medicinal chemistry.^16^ Therefore, this system was then extended to the amination of other natural product derivatives (4c–4g) containing β-methoxy γ-lactone subunit (Table 2).^17^ Arglabin derivative 4c underwent the amination to give the product (5cd) in 99% yield, which is equivalent to the commercially available antitumor agent Arglabin-DMA.^16*a*,18^ Michelolide derivative (4d and 4e) gave similarly good yields, in which the epoxy subunit does not affect the yield under the optimized conditions.^19^ The costunolide derivative 4f was converted to the corresponding product 5fd in 60% yield, while the reaction based on the parthenolide derivative 4g gave the desired product 5gd in 48% yield.

**Table 2:** Evaluation of the substrate scope of β-methoxy γ-lactones of natural products^a^

Entry	Substrate	Product	Yield^b^ (%)
1	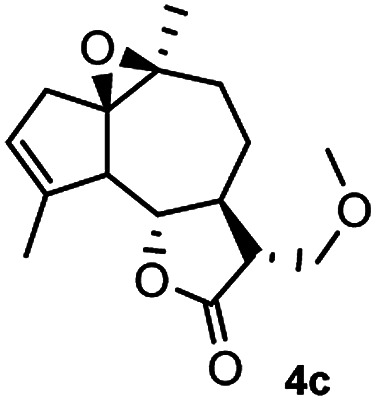	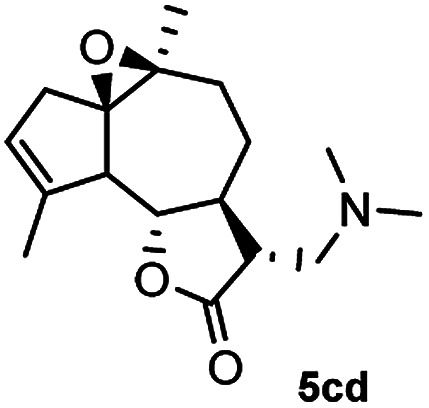	99
2	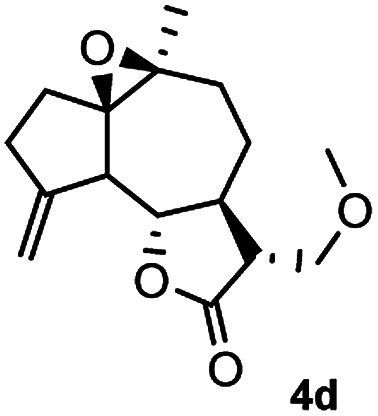	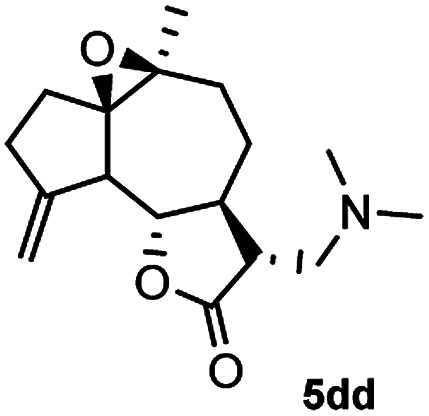	70
3	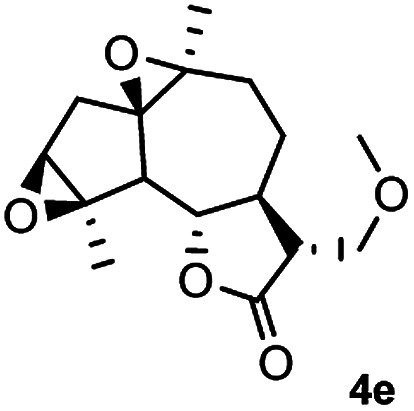	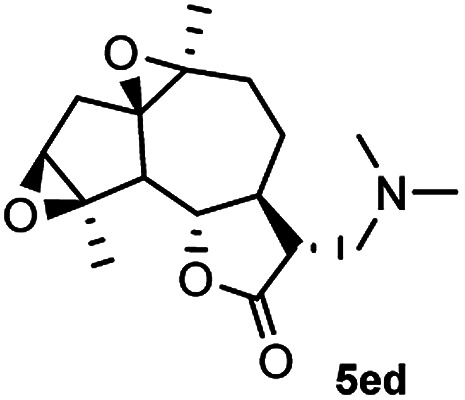	61^c^
4	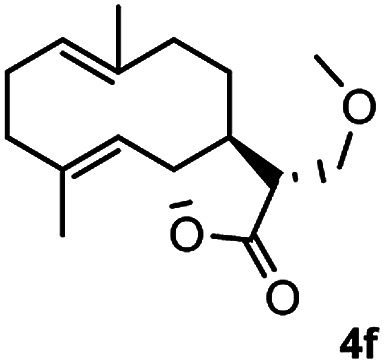	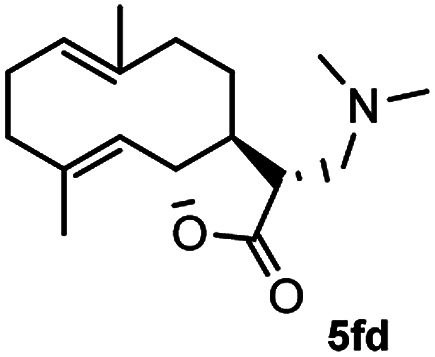	60
5	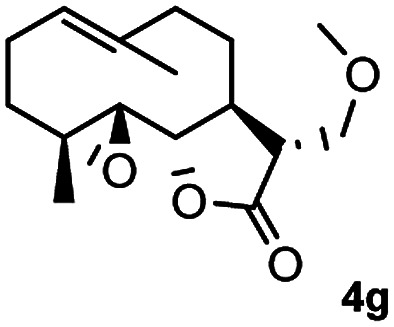	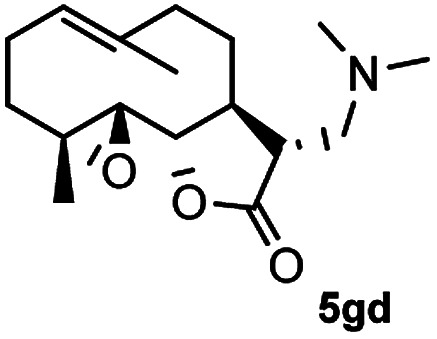	48

a Reactions were carried out with 4 (1.0 equiv.), 2d (2.0 equiv.) and LiCl (2.0 equiv.) in ^i^PrOH (0.15 M) at 120 °C for 5 h in sealed tube.

b Yields of isolated products are given.

c Reaction was conducted for 18 h.

The investigation on the mechanism of reaction was conducted by detailed control experiments as follows (Scheme 3): first, *N*-(3-methoxypropyl)aniline (6a) and 2-methoxy-*N*-phenylacetamide (7a) were prepared and subjected to the previously described standard condition respectively (Scheme 3a). In these reactions, no reaction took place, suggesting that the subunit of carbonyl β-ethers was essential for this reaction. Second, the desired product 3aa was obtained under the standard reaction conditions when the substrates bearing either 3-benzyloxy or 3-phenoxyl groups were used as the starting materials (Scheme 3b). Thus, these results supported a mechanism that there would undergo an intermediate in common. Moreover, the expected product 3aa was not observed when the reaction of 1a without LiCl was examined (Table 1, entry 7), verifying that LiCl also essential in this elimination process. Finally, the reaction of 3-methoxy-*N*-phenylacetamide (1a) under the standard reaction condition was examined in absence of amine, only trace of eliminate product (9a) was observed (Scheme 3c). Subsequently, when 2 equiv. of 1-methylpiperidine (2j) was added to the reaction above, both α,β-unsaturated amide 9a and the 3-isopropyl substituted product 10a were isolated in 27% and 21% yield respectively. Then the reaction of eliminate product 9a and piperidine 2a was examined, and the desired product 3aa was afforded in 68% yield, which indicated that elimination and addition process would be involved in this procedure. These experiments provided evidence that the amine 2 not only reacted as the substrate, but also exhibited the basicity in favor of the formation of the α,β-unsaturated product.

**Scheme 3:**
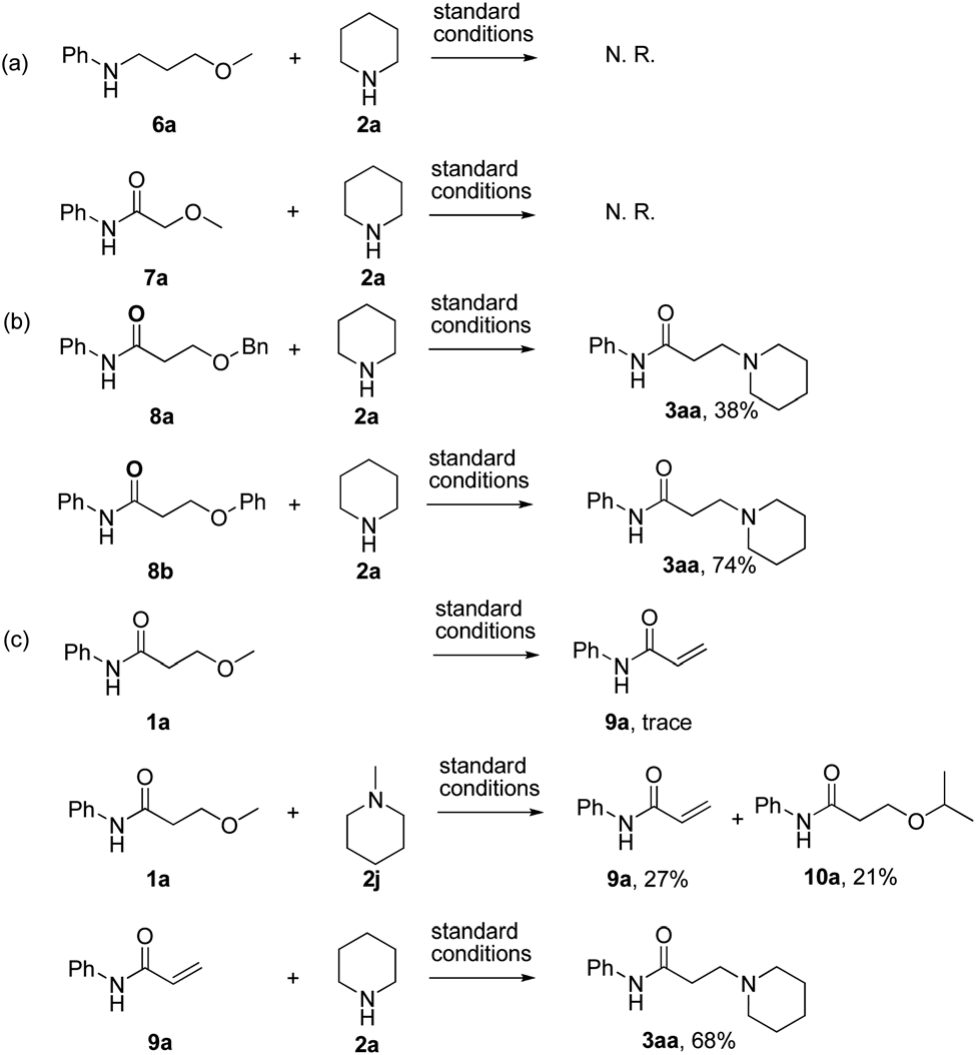
Control experiments. ^*a*^Reactions were carried out with 6a, 7a, 8a, 8b and 1a (1.0 equiv.), 2a and 2j (2.0 equiv.) and LiCl (2.0 equiv.) in ^i^PrOH (0.15 M) at 120 °C for 12 h in sealed tube. Yields of isolated products are given.

On the basis of the aforementioned mechanistic studies, a tentative pathways was proposed in Scheme 4: (1) the chelation between Li cation and oxygen atoms gives the intermediate I, which would accelerate the following elimination reaction step; (2) the elimination of MeOH leads to the α,β-unsaturated amide 9a; (3) the Michael addition of an amine to 9a affords the corresponding enolate II; (4) the tautomerization of II generates the product 3a.

**Scheme 4:**
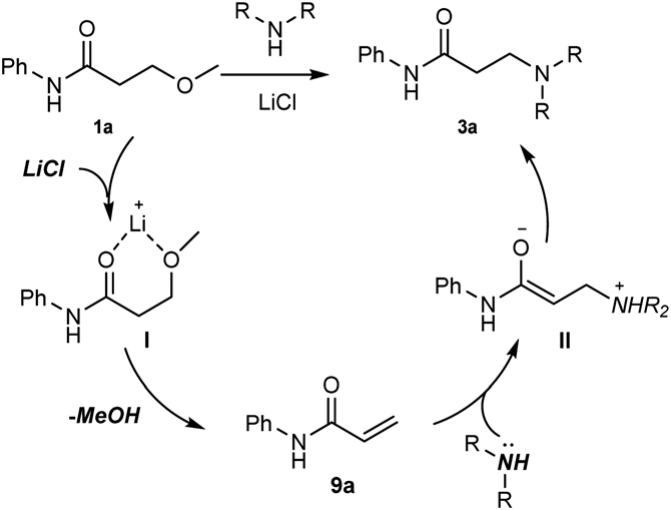
Tentative pathways of the reaction.

In conclusion, we reported a novel strategy for the synthesis of the β-amino amides (γ-lactones). The reaction shows a broad substrate scope for β-methoxy amides (γ-lactones) and a wide range of natural product derivatives including michelolide, costunolide and parthenolide derivatives. Moreover, this amination reaction provides an alternative β-position hydrophilic modification route of γ-lactones in medicinal chemistry, which would proceeds through two steps, which includes the initial formation of the α,β-unsaturated amide by the elimination of MeOH followed by the Michael addition with amines. Further investigation on detailed applications is currently underway.

## Conflicts of interest

There are no conflicts to declare.

## Supplementary Material

RA-010-D0RA07170F-s001

RA-010-D0RA07170F-s002
