# Supplementary material for: LiCl-promoted amination of β-methoxy amides (γ-lactones)
Source: RSC Adv. 2020 Sep 21;10(57):34938–42. doi: 10.1039/d0ra07170f (PMC9056935; doi:10.1039/d0ra07170f)
Supplement: RA-010-D0RA07170F-s001 [file RA-010-D0RA07170F-s001.pdf]

Electronic Supplementary Material (ESI) for RSC Advances.
This journal is © The Royal Society of Chemistry 2020

Supporting Information

LiCl-Promoted Amination of β-Methoxy amides (*γ*-Lactones)

Ru Zhao[1], Binglin Zeng[1], Wenqiang Jia[1], Hongyi Zhao[1], Longying Shen[1], Xiaojian Wang[1]*, Xiandao Pan[1,2]*.

[1] State Key Laboratory of Bioactive Substances and Functions of Natural Medicines, Institute of Materia Medica, Peking Union Medical College and Chinese Academy of Medical Sciences. Beijing 100050, China

[2]School of Pharmacy, Anhui University of Chinese Medicine, Hefei 230012, China

Supporting Information

Table of Contents

1. General remarks S2

2. Synthesis S3

3. NMR spectra S26

1. General remarks

Unless otherwise noted, all reagents and solvents were purchased from Innochem Co., Ltd. and Aldrich Inc. Flash column chromatography was performed with silica gel (200-300 mesh, 300-400 mesh, Qingdao Haiyang Chemical Co., Ltd.). Melting points (not corrected) were measured with RY-2. Optical rotations were measured with a Perkin-Elmer 240. NMR spectra were taken on MERCURY-400M or BRUKER-500M spectrometer. HRMS (ESI) experiments were carried out on a LC-ESI-JMS T100CS.

2. Synthesis

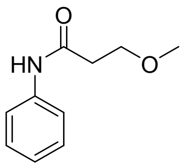

3-Methoxy-*N*-phenylpropanamide (1a). To a suspension of aniline (1.02 g, 1.0 eq) in dichloromethane (DCM, 15 mL) was added 3-methoxypropanoic acid (1.25 g, 1.1 eq), *N*1-((Ethylimino)methylene)-*N*3,*N*3-dimethylpropane-1,3-diamine hydrochloride (EDCI, 2.3 g, 1.1 eq)，and triethylamine (4.5 mL, 3.0 eq), subsequently. After stirring at room temperature for 12 hours, the reaction mixture was added diluted with EA and washed with 5% HCl aqueous solution, water and brine, dried over $MgSO_4$ and concentrated under reduce pressure. The residue was purified by column chromatography on silica gel to afford **1a** (1.50 g, 69%) as a yellow oil. ^1H NMR (400 MHz, $CDCl_3$) δ 8.32 (s, 1H), 7.51 (d, J = 8.0 Hz, 2H), 7.37 – 7.22 (m, 2H), 7.09 (d, J = 7.4 Hz, 1H), 3.73 (m, 2H), 3.54 – 3.36 (s, 3H), 2.71 – 2.52 (m, 2H).

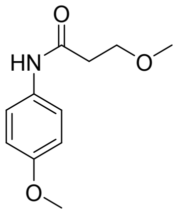

3-Methoxy-*N*-(4-methoxyphenyl) propenamide (1b). According to the general procedure analogous to that described for **1a**, **1b** (1.59 g, 76%) was obtained from *p*-Anisidin (1.23g, 10 mmol) as a gray solid. Mp: 56-58 ^oC; ^1H NMR (400 MHz, $CDCl_3$) δ 8.16 (s, 1H), 7.49 – 7.35 (m, 2H), 6.95 – 6.72 (m, 2H), 3.78 (s, 3H), 3.72 (t, J = 5.7 Hz, 2H), 3.43 (s, 3H), 2.61 (t, J = 5.7 Hz, 2H); ^{13}C NMR (100 MHz, $CDCl_3$) δ 169.6, 156.3, 131.2, 121.7, 114.1, 68.7, 58.9, 55.5, 37.8; HRMS (ESI) m/z calcd. for $C_{11}H_{16}O_3N$ $[M+H]^+$ 210.10519, found 210.11269.

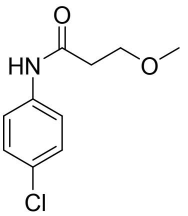

***N*-(4-Chlorophenyl)-3-methoxypropanamide(1c).** According to the general procedure analogous to that described for **1a**, **1c** (1.50 g, 70%) was obtained from 4-chloroaniline (1.28g, 10 mmol) as a white solid. Mp: 60-62 ^{o}C; ^{1}H NMR (400 MHz, $CDCl_3$) δ 8.27 (s, 1H), 7.54 – 7.38 (m, 2H), 7.30 – 7.23 (m, 2H), 3.72 (t, J=5.6Hz, 2H), 3.45 (s, 3H), 2.63 (t, J=5.6Hz, 2H); ^{13}C NMR (100 MHz, $CDCl_3$) δ 169.8, 136.6, 129.0, 128.9, 121.1, 68.5, 59.0, 37.9; HRMS (ESI) m/z calcd. for $C_{10}H_{13}O_2NCl$ $[M+H]^+$ 214.05566, found 214.06340.

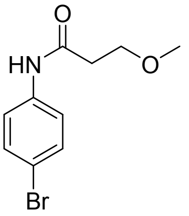

***N*-(4-Bromophenyl)-3-methoxypropanamide (1d).** According to the general procedure analogous to that described for **1a**, **1d** (1.89g, 73%) was obtained from 4-chloroaniline (1.72g, 10 mmol) as a white solid. Mp: 58-60 ^{o}C; ^{1}H NMR (400 MHz, $CDCl_3$) δ 8.31 (s, 1H), 7.41 (s, 4H), 3.72 (t, J = 5.6 Hz, 2H), 3.45 (s, 3H), 2.62 (t, J = 5.6 Hz, 2H).

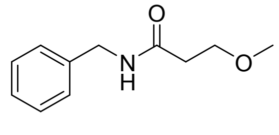

***N*-Benzyl-3-methoxypropanamide (1e).** To a suspension of benzylamine (547 μL, 5 mmol) in DMF (8 mL) was added 3-methoxypropanoic acid (500 mg, 5 mmol), EDCI (1.5 g, 7.5 mmol)， 1-Hydroxybenzotriazole (675 mg, 5 mmol) and triethylamine (1.4 mL, 10 mmol), subsequently. After stirring at room temperature for 12 hours, the reaction mixture was diluted with EA (30 mL) and washed with saturated $NaHCO_3$ aqueous solution (30 mL), NH_4Cl aqueous solution (30 mL), water (30 mL) and brine (30 mL), dried over $MgSO_4$ and concentrated under reduce pressure. The residue

was purified by column chromatography on silica gel to afford **1e** (314 mg, 33%) as a yellow solid. Mp: 48-50 °C; ^{1}H NMR (400 MHz, $CDCl_3$) δ 7.34 – 7.22 (m, 5H), 4.44 (d, J = 5.7 Hz, 2H), 3.71 – 3.55 (m, 2H), 3.38 – 3.24 (s, 3H), 2.49 (t, J = 5.7 Hz, 2H).

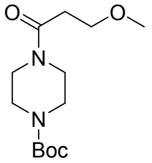

tert-Butyl 4-(3-methoxypropanoyl) piperazine-1-carboxylate (1f). To a suspension of 1-boc-piperazine (745 mg, 4 mmol) in DMF (8 mL) was added 3-methoxypropanoic acid (500 mg, 5 mmol), EDCI (1.5 g, 7.5 mmol)， and 1-Hydroxybenzotriazole (675 mg, 5 mmol). After stirring at room temperature for 12 hours, the reaction mixture was added EA (30 mL) and was washed with $NaHCO_3$ aqueous solution (30 mL), NH_4Cl aqueous solution (30 mL), H_2O (30 mL) and NaCl aqueous solution (30 mL), dried over $MgSO_4$ and concentrated. The residue was purified by column chromatography on silica gel to afford **1f** (676mg, 62%) as a white solid. Mp: 68-70 °C; ^{1}H NMR (500 MHz, $CDCl_3$) δ 3.75 (t, J = 6.1 Hz, 2H), 3.65 (s, 2H), 3.48 (d, J = 14.0 Hz, 6H), 3.40 (s, 3H), 2.66 (t, J = 6.1 Hz, 2H), 1.52 (s, 9H).

General procedure for the amination of carbonyl *β*-methoxy group in the presence of LiCl.

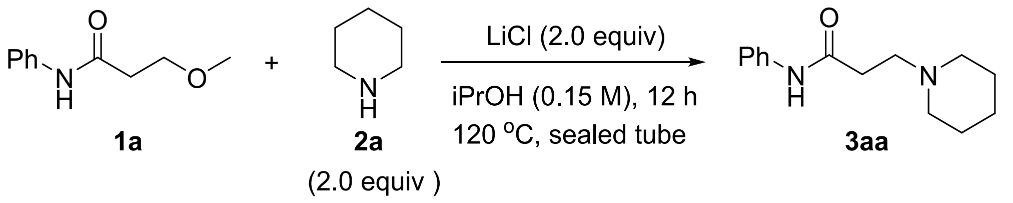

***N*-Phenyl-3-(piperidin-1-yl) propenamide (3aa).** To a mixture of **1a** (150 mg, 0.8 mmol) and **2a** (136 mg, 1.6 mmol) in *i*-PrOH (5.3 mL) was added LiCl (67mg, 1.6 mmol). The mixture was stirred at 120 °C for 12 hours in a sealed tube. After cooling down to room temperature, the reaction mixture was added diluted with DCM (10 mL) was washed with saturated $NaHCO_3$ aqueous solution (10 mL), water (10 mL) and brine (10 mL),

dried over $MgSO_4$ and concentrated under reduce pressure. The residue was purified by column chromatography on silica gel to afford **3aa** (130 mg, 70%) as a yellow oil. ^{1}H NMR (400 MHz, $CDCl_3$) δ 11.21 (s, 1H), 7.59 – 7.50 (m, 2H), 7.35 – 7.28 (m, 2H), 7.11 – 7.02 (m, 1H), 2.76 – 2.68 (m, 2H), 2.65 – 2.50 (m, 6H), 1.77 – 1.67 (m, 4H), 1.56 (s, 2H); ^{13}C NMR (100 MHz, $CDCl_3$) δ 170.7, 138.9, 129.0, 123.6, 119.5, 54.4, 53.7, 32.6, 26.1, 24.1; HRMS (ESI) m/z calcd. for $C_{14}H_{21}ON_2$ $[M+H]^+$ 233.15756, found 233.16441.

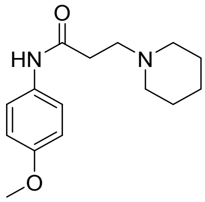

***N*-(4-Methoxyphenyl)-3-(piperidin-1-yl) propenamide (3ba**). According to the general procedure analogous to that described for **3aa**, **3ba** (132 mg, 72%) was obtained from **1b** (150 mg, 0.7 mmol) as a white solid. Mp: 104-106 °C; ^{1}H NMR (400 MHz, $CDCl_3$) δ 11.14 (s, 1H), 7.54 – 7.38 (m, 2H), 7.00 – 6.74 (m, 2H), 3.78 (s, 3H), 2.70 – 2.63 (m, 2H), 2.51 (m, 6H), 1.74 – 1.63 (m, 4H), 1.54 (s, 2H); ^{13}C NMR (100 MHz, $CDCl_3$) δ 170.5, 155.8, 132.3, 120.9, 114.2, 55.5, 54.4, 53.7, 32.44, 26.2, 24.2; HRMS (ESI) m/z calcd. for $C_{15}H_{23}O_2N_2$ $[M+H]^+$ 263.16813, found 263.17493.

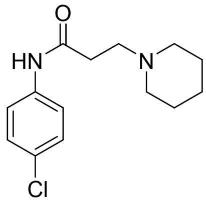

***N*-(4-Chlorophenyl)-3-(piperidin-1-yl) propenamide (3ca**). According to the general procedure analogous to that described for **3aa**, **3ca** (134 mg, 72%) was obtained from **1c** (150 mg, 0.7 mmol) as a yellow solid. Mp: 70-72 °C; ^{1}H NMR (400 MHz, $CDCl_3$) δ 11.44 (s, 1H), 7.53 – 7.46 (m, 2H), 7.27 – 7.23 (m, 2H), 2.71 – 2.64 (m, 2H), 2.52 (m, 6H), 1.75 – 1.62 (m, 4H), 1.56 (m, 2H); ^{13}C NMR (100 MHz, $CDCl_3$) δ 170.8, 137.6, 129.0, 128.3, 120.6, 54.3, 53.6, 32.5, 26.2, 24.2; HRMS (ESI) m/z calcd. for $C_{14}H_{20}ON_2Cl$ $[M+H]^+$ 267.11859, found 267.12555.

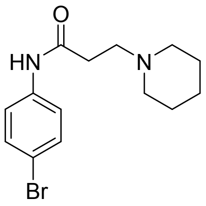

***N*-(4-Bromophenyl)-3-(piperidin-1-yl) propenamide (3da).** According to the general procedure analogous to that described for **3aa**, **3da** (143 mg, 77%) was obtained from **1d** (150 mg, 0.6 mmol) as a yellow solid. Mp: 74-76 ^{o}C; ^{1}H NMR (400 MHz, $CDCl_3$) *δ* 11.44 (s, 1H), 7.47 – 7.42 (m, 2H), 7.42 – 7.37 (m, 2H), 2.67 (m, 2H), 2.63 – 2.47 (m, 6H), 1.74 – 1.64 (m, 4H), 1.56 (m, 2H);^{13}C NMR (100 MHz, $CDCl_3$) *δ* 170.9, 138.1, 131.9, 121.0, 115.9, 54.2, 53.6, 32.5, 26.2, 24.1; HRMS (ESI) m/z calcd. for $C_{14}H_{20}ON_2Br$ $[M+H]^+$ 311.06808, found 311.07364.

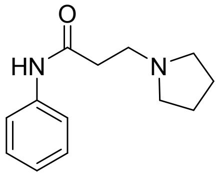

***N*-Phenyl-3-(pyrrolidin-1-yl) propenamide (3ab).** According to the general procedure analogous to that described for **3aa**, **3ab** (127 mg, 73%) was obtained from **1a** (150 mg, 0.8 mmol) as a yellow oil. ^{1}H NMR (400 MHz, $CDCl_3$) *δ* 11.13 (s, 1H), 7.49 (m, 2H), 7.28 (m, 2H), 7.04 (t, *J* = 7.4 Hz, 1H), 2.83 (t, *J* = 6.0 Hz, 2H), 2.65 (s, 4H), 2.56 – 2.47 (m, 2H), 1.88 (s, 4H). ^{13}C NMR (100 MHz, $CDCl_3$) *δ* 170.9, 138.9, 128.9, 123.6, 119.7, 53.2, 51.4, 34.7, 23.7; HRMS (ESI) m/z calcd. for $C_{13}H_{19}ON_2$ $[M+H]^+$ 219.14191, found 219.14943.

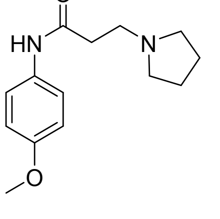

***N*-(4-Methoxyphenyl)-3-(pyrrolidin-1-yl) propenamide (3bb).** According to the general procedure analogous to that described for **3aa**, **3bb** (120 mg, 69%) was obtained from **1b** (150 mg, 0.7 mmol) as a white

solid. Mp: 76-80 °C; ^{1}H NMR (400 MHz, $CDCl_3$) δ 11.19 – 10.73 (m, 1H), 7.40 (dd, J = 9.0 Hz, 2H), 6.84 (d, J = 9.0 Hz, 2H), 3.78 (s, 3H), 2.88 – 2.80 (m, 2H), 2.67 (t, J = 8.6, 3.5 Hz, 4H), 2.56 – 2.48 (m, 2H), 1.88 (m, 4H); ^{13}C NMR (100 MHz, $CDCl_3$) δ 170.5, 155.8, 132.2, 121.2, 114.1, 55.5, 53.2, 51.5, 34.6, 23.7; HRMS (ESI) m/z calcd. for $C_{14}H_{21}O_2N_2$ $[M+H]^+$ 249.15248, found 249.16005.

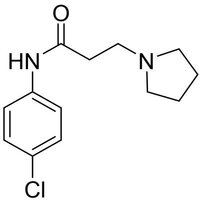

***N*-(4-Chlorophenyl)-3-(pyrrolidin-1-yl) propenamide (3cb).** According to the general procedure analogous to that described for **3aa**, **3cb** (118 mg, 67%) was obtained from **1c** (150 mg, 0.7 mmol) as a yellow solid. Mp: 88-90 °C; ^{1}H NMR (400 MHz, $CDCl_3$) δ 10.67 (s, 1H), 7.57 (d, J = 8.8 Hz, 2H), 7.22 (d, J = 8.8 Hz, 2H), 3.24 (t, J = 6.0 Hz, 2H), 3.06 (s, 4H), 2.91 (t, J = 6.0 Hz, 2H), 2.00 (m, 4H); ^{13}C NMR (100 MHz, $CDCl_3$) δ 169.1, 137.2, 128.9, 128.7, 121.0, 53.8, 51.3, 33.8, 23.5; HRMS (ESI) m/z calcd. for $C_{13}H_{18}ON_2Cl$ $[M+H]^+$ 253.10294, found 253.11082.

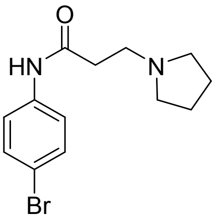

***N*-(4-Bromophenyl)-3-(pyrrolidin-1-yl) propenamide (3db).** According to the general procedure analogous to that described for **3aa**, **3db** (130 mg, 73%) was obtained from **1d** (150 mg, 0.6 mmol) as a white solid. Mp: 140-142 °C; ^{1}H NMR (400 MHz, $CDCl_3$) δ 9.85 (s, 1H), 7.61 (d, J = 8.8 Hz, 2H), 7.40 (d, J = 8.8 Hz, 2H), 3.51 (t, J = 6.6 Hz, 2H), 3.16 (t, J = 6.6 Hz, 2H), 2.12 (s, 4H), 1.74 (s, 4H); ^{13}C NMR (100 MHz, $CDCl_3$) δ 167.6, 137.3, 131.8, 121.5, 116.9, 54.4, 51.6, 33.5, 23.4; HRMS (ESI) m/z calcd. for $C_{13}H_{18}ON_2Br$ $[M+H]^+$ 297.05243, found 297.06082.

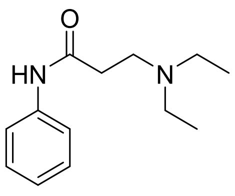

3-(Diethylamino)-*N*-phenylpropanamide (3ac). According to the general procedure analogous to that described for **3aa**, **3ac** (110 mg, 63%) was obtained from **1a** (150 mg, 0.8 mmol) as a yellow oil. ^{1}H NMR (400 MHz, $CDCl_3$) δ 10.93 (s, 1H), 7.62 – 7.53 (m, 2H), 7.33 – 7.24 (m, 2H), 7.06 (t, J = 7.4 Hz, 1H), 2.95 (t, J = 6.2 Hz, 2H), 2.80 (q, J = 7.2 Hz, 4H), 2.69 (t, J = 6.2 Hz, 2H), 1.19 (t, J = 7.2 Hz, 6H); ^{13}C NMR (100 MHz, $CDCl_3$) δ 170.1, 138.7, 128.9, 123.7, 119.7, 48.9, 46.3, 32.8, 10.7; HRMS (ESI) m/z calcd. for $C_{13}H_{21}ON_2$ $[M+H]^+$ 221.15756, found 221.16490.

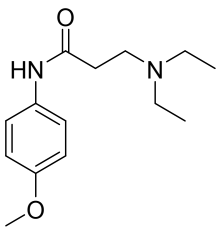

3-(Diethylamino)-*N*-(4-methoxyphenyl) propenamide (3bc). According to the general procedure analogous to that described for **3aa**, **3bc** (137 mg, 78%) was obtained from **1b** (150 mg, 0.7 mmol) as a yellow oil. ^{1}H NMR (400 MHz, $CDCl_3$) δ 10.45 (s, 1H), 7.52 (d, J = 8.8 Hz, 2H), 6.78 (d, J = 8.8 Hz, 2H), 3.73 (d, J = 1.0 Hz, 3H), 3.17 (t, J = 6.3 Hz, 2H), 2.98 – 2.86 (m, 6H), 1.25 (t, J = 7.3 Hz, 6H); ^{13}C NMR (100 MHz, $CDCl_3$) δ 167.8, 156.2, 131.6, 121.6, 114.0, 55.5, 48.7, 47.0, 31.9, 8.8; HRMS (ESI) m/z calcd. for $C_{14}H_{23}O_2N_2$ $[M+H]^+$ 251.16813, found 251.17552.

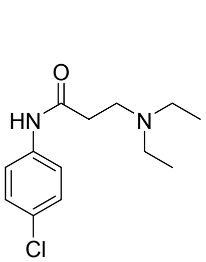

***N*-(4-Chlorophenyl)-3-(diethylamino) propenamide (3cc).** According to the general procedure analogous to that described for **3aa**, **3cc** (124 mg, 70%) was obtained from **1c** (150 mg, 0.7 mmol) as a yellow oil. ^{1}H NMR (400 MHz, $CDCl_3$) δ 11.36 (s, 1H), 7.52 – 7.47 (m, 2H), 7.28 – 7.22 (m,

2H), 2.83 – 2.76 (m, 2H), 2.69 (q, J = 7.2 Hz, 4H), 2.56 – 2.48 (m, 2H), 1.13 (dd, J = 7.7, 6.7 Hz, 6H); ^{13}C NMR (100 MHz, $CDCl_3$) δ 170.6, 137.4, 128.9, 128.4, 120.7, 48.9, 46.1, 32.9, 11.2; HRMS (ESI) m/z calcd. for $C_{13}H_{20}ON_2Cl$ $[M+H]^+$ 255.11859, found 255.12634.

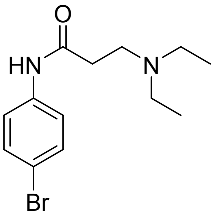

***N*-(4-Bromophenyl)-3-(diethylamino) propenamide (3dc).** According to the general procedure analogous to that described for **3aa**, **3dc** (138 mg, 77%) was obtained from **1d** (150 mg, 0.6 mmol) as a yellow oil. ^{1}H NMR (400 MHz, $CDCl_3$) δ 10.24 (s, 1H), 7.50 – 7.43 (m, 2H), 7.41 – 7.34 (m, 2H), 3.15 (t, J = 6.4 Hz, 2H), 2.97 (q, J = 7.2 Hz, 4H), 2.81 (t, J = 6.4 Hz, 2H), 1.25 (t, J = 7.2 Hz, 6H); ^{13}C NMR (100 MHz, $CDCl_3$) δ 169.8, 137.2, 131.9, 121.6, 116.8, 48.9, 46.8, 31.4, 9.9; HRMS (ESI) m/z calcd. for $C_{13}H_{20}ON_2Br$ $[M+H]^+$ 299.06808, found 299.07632.

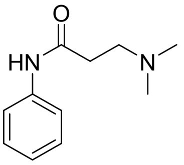

3-(Dimethylamino)-*N*-phenylpropanamide (3ad). According to the general procedure analogous to that described for **3aa**, **3ad** (98 mg, 64%) was obtained from **1a** (150 mg, 0.8 mmol) as a yellow solid. Mp: 52-54 ^{o}C; ^{1}H NMR (400 MHz, $CDCl_3$) δ 10.78 (s, 1H), 7.56 (dd, J = 8.5, 0.9 Hz, 2H), 7.33 (t, J = 7.9 Hz, 2H), 7.10 (t, J = 7.4 Hz, 1H), 2.78 – 2.71 (m, 2H), 2.62 – 2.54 (m, 2H), 2.43 (s, 6H); ^{13}C NMR (100 MHz, $CDCl_3$) δ 170.5, 138.7, 128.9, 123.7, 119.9, 55.0, 44.3, 33.5; HRMS (ESI) m/z calcd. for $C_{11}H_{17}ON_2$ $[M+H]^+$ 193.12626, found 193.13383.

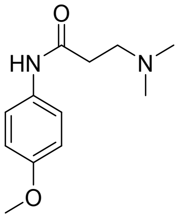

3-(Dimethylamino)-*N*-(4-methoxyphenyl) propenamide (3bd). According to the general procedure analogous to that described for **3aa**, **3bd** (113 mg, 73%) was obtained from **1b** (150 mg, 0.7 mmol) as a yellow solid. Mp: 50-52 °C; ^{1}H NMR (400 MHz, $CDCl_3$) δ 10.70 (s, 1H), 7.54 – 7.36 (m, 2H), 6.93 – 6.78 (m, 2H), 3.80 (d, J = 1.2 Hz, 3H), 2.72 – 2.63 (m, 2H), 2.51 (m, 2H), 2.38 (s, 6H); ^{13}C NMR (100 MHz, $CDCl_3$) δ 170.5, 155.9, 132.0, 121.5, 114.1, 55.5, 55.2, 44.5, 33.4; HRMS (ESI) m/z calcd. for $C_{12}H_{19}O_2N_2$ [M+H]$^+$ 223.13683, found 223.14442.

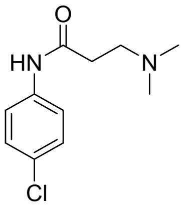

***N*-(4-Chlorophenyl)-3-(dimethylamino) propenamide (3cd).** According to the general procedure analogous to that described for **3aa**, **3cd** (106 mg, 67%) was obtained from **1c** (150 mg, 0.7 mmol) as a white olid. Mp: 56-62 °C; ^{1}H NMR (400 MHz, $CDCl_3$) δ 11.05 (s, 1H), 7.53 – 7.45 (m, 2H), 7.33 – 7.23 (m, 2H), 2.72 – 2.64 (m, 2H), 2.56 – 2.49 (m, 2H), 2.40 (s, 6H); ^{13}C NMR (100 MHz, $CDCl_3$) δ 170.8, 137.3, 128.9, 128.4, 121.1, 55.0, 44.4, 33.3; HRMS (ESI) m/z calcd. for $C_{11}H_{16}ON_2Cl$ [M+H]$^+$ 227.08729, found 227.09523.

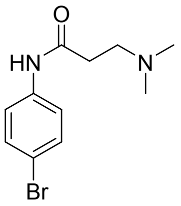

***N*-(4-Bromophenyl)-3-(dimethylamino) propenamide (3dd).** According to the general procedure analogous to that described for **3aa**, **3dd** (104 mg, 64%) was obtained from **1d** (150 mg, 0.6 mmol) as a yellow solid. Mp: 52-56 °C; ^{1}H NMR (400 MHz, $CDCl_3$) δ 11.23 – 10.77 (m, 1H), 7.45 – 7.33

(m, 4H), 2.63 (t, $J = 6.6, 5.0$ Hz, 2H), 2.47 (t, $J = 6.6, 4.9$ Hz, 2H), 2.35 (s, 6H); ^{13}C NMR (100 MHz, $CDCl_3$) δ 170.8, 137.9, 131.8, 121.4, 116.0, 55.1, 44.5, 33.4; HRMS (ESI) m/z calcd. for $C_{11}H_{16}ON_2Br$ $[M+H]^+$ 271.03678, found 271.04501.

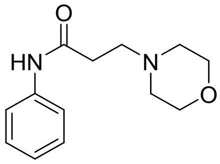

3-Morpholino-*N*-phenylpropanamide (3ae). According to the general procedure analogous to that described for **3aa**, **3ae** (120 mg, 64%) was obtained from **1a** (150 mg, 0.8 mmol) as a white solid. Mp: 62-64 ^oC; ^1H NMR (400 MHz, $CDCl_3$) δ 10.71 (s, 1H), 7.53 (dd, $J = 8.5, 1.0$ Hz, 2H), 7.37 – 7.28 (m, 2H), 7.13 – 7.04 (m, 1H), 3.82 (t, $J = 4.6$ Hz, 4H), 2.79 – 2.70 (m, 2H), 2.62 (s, 4H), 2.57 – 2.50 (m, 2H); ^{13}C NMR (100 MHz, $CDCl_3$) δ 170.3, 138.6, 129.1, 123.8, 119.5, 67.1, 54.2, 52.9, 32.3; HRMS (ESI) m/z calcd. for $C_{13}H_{19}O_2N_2$ $[M+H]^+$ 235.13683, found 235.14270.

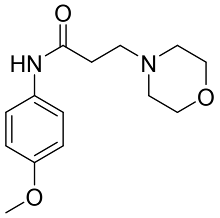

***N*-(4-Methoxyphenyl)-3-morpholinopropanamide (3be).** According to the general procedure analogous to that described for **3aa**, **3be** (122 mg, 66%) was obtained from **1b** (150 mg, 0.7 mmol) as a yellow solid. Mp: 110-112 ^oC; ^1H NMR (400 MHz, $CDCl_3$) δ 10.48 (s, 1H), 7.41 (d, $J = 8.9$ Hz, 2H), 6.92 – 6.73 (m, 2H), 3.90 – 3.66 (m, 7H), 2.69 (t, $J = 5.9$ Hz, 2H), 2.56 (s, 4H), 2.49 (t, $J = 5.9$ Hz, 2H); ^{13}C NMR (100 MHz, $CDCl_3$) δ 170.1, 156.0, 131.9, 121.1, 114.2, 67.1, 55.5, 54.3, 52.9, 32.3; HRMS (ESI) m/z calcd. for $C_{14}H_{21}O_3N_2$ $[M+H]^+$ 265.14739, found 265.15491.

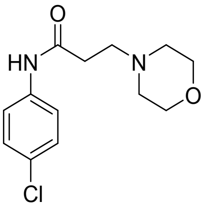

***N*-(4-Chlorophenyl)-3-morpholinopropanamide (3ce).** According to the general procedure analogous to that described for **3aa**, **3ce** (120 mg, 64%) was obtained from **1c** (150 mg, 0.7 mmol) as a white solid. Mp: 94-96 °C; ^{1}H NMR (400 MHz, $CDCl_3$) δ 10.84 (s, 1H), 7.53 – 7.45 (m, 2H), 7.28 (dd, J = 7.4, 4.0 Hz, 2H), 3.82 (t, J = 4.5 Hz, 4H), 2.79 – 2.70 (m, 2H), 2.62 (s, 4H), 2.57 – 2.50 (m, 2H); ^{13}C NMR (100 MHz, $CDCl_3$) δ 170.3, 137.2, 129.0, 128.6, 120.7, 67.1, 54.1, 52.8, 32.2; HRMS (ESI) m/z calcd. for $C_{13}H_{18}O_2N_2Cl$ $[M+H]^+$ 269.09786, found 269.10410.

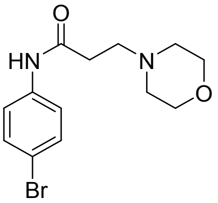

***N*-(4-Bromophenyl)-3-morpholinopropanamide (3de).** According to the general procedure analogous to that described for **3aa**, **3de** (140 mg, 75%) was obtained from **1d** (150 mg, 0.6 mmol) as a white solid. Mp: 82-84 °C; ^{1}H NMR (400 MHz, $CDCl_3$) δ 10.82 (s, 1H), 7.50 – 7.34 (m, 4H), 3.83 – 3.76 (m, 4H), 2.76 – 2.68 (m, 2H), 2.60 (s, 4H), 2.55 – 2.48 (m, 2H); ^{13}C NMR (100 MHz, $CDCl_3$) δ 170.4, 137.7, 132.0, 121.1, 116.2, 67.1, 54.1, 52.8, 32.2; HRMS (ESI) m/z calcd. for $C_{13}H_{18}O_2N_2Br$ $[M+H]^+$ 313.04734, found 313.05566.

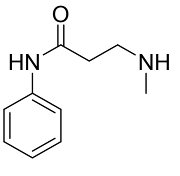

3-(methylamino)-N-phenylpropanamide (3af). According to the general procedure analogous to that described for **3aa**, **3af** (22 mg, 15%) was obtained from **1d** (150 mg, 0.8 mmol) as a colorless oil. ^{1}H NMR (400 MHz, CD_3OD) δ 7.55 (d, J = 7.7 Hz, 2H), 7.29 (t, J = 7.9 Hz, 2H), 7.07 (t, J = 7.4 Hz, 1H), 2.88 (t, J = 6.7 Hz, 2H), 2.57 (t, J = 6.8 Hz, 2H), 2.40 (s,

3H); ^{13}C NMR (100 MHz, CD_3OD) δ 172.6, 139.8, 129.8, 125.1, 121.2, 48.2, 36.8, 35.8. HRMS (ESI) m/z calcd. for $C_{10}H_{15}N_2O$ $[M+H]^+$ 179.11789, found 179.11823.

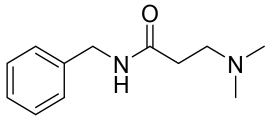

***N*-Benzyl-3-(dimethylamino) propenamide (3ed).** According to the general procedure analogous to that described for **3aa**, **3ed** (170 mg, 83%) was obtained from **1e** (200 mg, 1 mmol) as a colorless oil. ^{1}H NMR (500 MHz, $CDCl_3$) δ 8.67 (s, 1H), 7.31 (m, 2H), 7.28 – 7.21 (m, 3H), 4.45 (d, *J* = 5.4 Hz, 2H), 2.55 (t, *J* = 5.8 Hz, 2H), 2.41 (t, *J* = 5.8 Hz, 2H), 2.22 (s, 6H); ^{13}C NMR (100 MHz, $CDCl_3$) δ 172.6, 139.0, 128.6, 127.3, 127.1, 55.3, 44.6, 43.0, 33.0; HRMS (ESI) m/z calcd. for $C_{12}H_{19}O_2N$ $[M+H]^+$ 207.14191, found 207.14923.

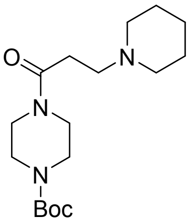

***tert*-Butyl 4-(3-(piperidin-1-yl) propanoyl) piperazine-1-carboxylate (3fa).** According to the general procedure analogous to that described for **3aa**, **3fa** (93 mg, 52%) was obtained from **1f** (150 mg, 0.55 mmol) as a white solid. Mp: 96-98 °C; ^{1}H NMR (400 MHz, $CDCl_3$) δ 3.64 – 3.52 (m, 2H), 3.50 – 3.30 (m, 6H), 2.76 – 2.65 (m, 2H), 2.58 (m, 2H), 2.45 (s, 4H), 1.69 – 1.54 (m, 4H), 1.45 (m, 11H); ^{13}C NMR (100 MHz, $CDCl_3$) δ 170.5, 154.6, 80.3, 54.8, 54.7, 45.4, 41.4, 31.1, 28.4, 25.8, 24.2; HRMS (ESI) m/z calcd. for $C_{17}H_{32}O_3N_3$ $[M+H]^+$ 326.23654, found 326.24277.

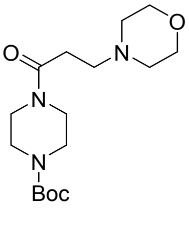

***tert*-Butyl 4-(3-morpholinopropanoyl) piperazine-1-carboxylate**

(**3fe**) **.** According to the general procedure analogous to that described for **3aa**, **3fe** (140 mg, 78%) was obtained from **1f** (150 mg, 0.55 mmol) as a white solid. Mp: 80-82 °C; ^{1}H NMR (400 MHz, $CDCl_3$) δ 3.65 (Brs, 4H), 3.54 (s, 2H), 3.38 (d, J = 17.9 Hz, 6H), 2.75 – 2.61 (m, 2H), 2.57 – 2.36 (m, 6H), 1.48 – 1.34 (m, 9H); ^{13}C NMR (100 MHz, $CDCl_3$) δ 170.2, 154.5, 80.4, 66.9, 54.4, 53.7, 45.4, 41.4, 30.8, 28.4; HRMS (ESI) m/z calcd. for $C_{16}H_{30}O_4N_3$ $[M+H]^+$ 328.21581, found 328.22339.

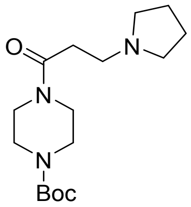

***tert*-Butyl 4-(3-(pyrrolidin-1-yl) propanoyl) piperazine-1-carboxylate (3fb).** According to the general procedure analogous to that described for **3aa**, **3fb** (121 mg, 71%) was obtained from **1f** (150 mg, 0.55 mmol) as a yellow solid. Mp: 88-90 °C; ^{1}H NMR (400 MHz, $CDCl_3$) δ 3.51 (Brs, 2H), 3.48 – 3.32 (m, 8H), 3.25 (s, 4H), 2.99 (t, J = 7.0 Hz, 2H), 2.06 (s, 4H), 1.41 (s, 9H); ^{13}C NMR (100 MHz, $CDCl_3$) δ 167.7, 154.4, 80.4, 54.0, 51.0, 45.2, 41.6, 29.6, 28.3, 23.2; HRMS (ESI) m/z calcd. for $C_{16}H_{30}O_3N_3$ $[M+H]^+$ 312.22089, found 312.22852.

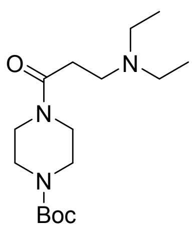

***tert*-Butyl 4-(3-(diethylamino) propanoyl) piperazine-1-carboxylat (3fc).** According to the general procedure analogous to that described for **3aa**, **3fc** (124 mg, 72%) was obtained from **1f** (150 mg, 0.55 mmol) as a yellow oil. ^{1}H NMR (400 MHz, $CDCl_3$) δ 3.60 – 3.53 (m, 2H), 3.43 (s, 4H), 3.41 – 3.35 (m, 2H), 2.93 – 2.78 (m, 2H), 2.57 (m, 6H), 1.45 (s, 9H), 1.06 (t, J = 7.2 Hz, 6H); ^{13}C NMR (100 MHz, $CDCl_3$) δ 170.5, 154.6, 80.3, 48.8, 47.0, 45.4, 41.4, 31.2, 28.4, 11.4; HRMS (ESI) m/z calcd. for $C_{16}H_{32}O_3N_3$ $[M+H]^+$ 314.23654, found 314.24405.

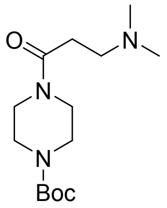

***tert*-Butyl 4-(3-(dimethylamino) propanoyl) piperazine-1-carboxylate (3fd).** According to the general procedure analogous to that described for **3aa**, **3fd** (110 mg, 70%) was obtained from **1f** (150 mg, 0.55 mmol) as a yellow solid. Mp: 78-80 °C; ^{1}H NMR (500 MHz, $CDCl_3$) δ 3.59 (Brs, 2H), 3.42 (m, 6H), 2.64 (t, J = 7.3 Hz, 2H), 2.51 (t, J = 7.4 Hz, 2H), 2.26 (s, 6H), 1.47 (s, 9H); ^{13}C NMR (100 MHz, $CDCl_3$) δ 170.4, 154.6, 80.3, 55.2, 45.6, 45.4, 41.4, 31.8, 28.4; HRMS (ESI) m/z calcd. for $C_{14}H_{28}O_3N_3$ $[M+H]^+$ 286.20524, found 286.21231.

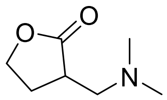

3-((Dimethylamino)methyl) dihydrofuran-2(3*H*)-one (5ad). According to the general procedure analogous to that described for **3aa**, except that the reaction was allowed for 5 hours. **5ad** (133 mg, 85%) was obtained from **4a** (142 mg, 1 mmol) as a colorless oil. ^{1}H NMR (400 MHz, $CDCl_3$) δ 4.32 (m, 1H), 4.16 (m, 1H), 2.74 – 2.61 (m, 2H), 2.49 – 2.34 (m, 2H), 2.22 (s, 6H), 2.12 – 2.02 (m, 1H); ^{13}C NMR (100 MHz, $CDCl_3$) δ 177.4, 65.8, 58.8, 44.5, 37.4, 27.0; HRMS (ESI) m/z calcd. for $C_7H_{14}O_2N$ $[M+H]^+$ 144.09463, found 144.10182.

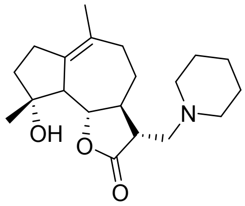

(3*R*,3a*S*,9*R*,9b*S*)-9-Hydroxy-6,9-dimethyl-3-(piperidin-1-ylmethyl)-3a,4,5,7,8,9,9a,9b-octahydroazuleno[4,5-b] furan-2(3*H*)-one (5ba). According to the general procedure analogous to that described for **3aa**, except that the reaction was allowed for 10 hours. **5ba** (111 mg, 93%) was obtained from **4b** (100 mg, 0.3 mmol) as a yellow oil. $[\alpha]^{20}_D$= +16.2 (*c* 0.47, $CHCl_3$); ^{1}H NMR (500 MHz, $CDCl_3$) δ 3.82 (s, 1H), 2.77 (s, 1H),

2.63 (d, J = 15.3 Hz, 3H), 2.52 – 2.31 (m, 5H), 2.17 (s, 3H), 2.02 (d, J = 16.5 Hz, 1H), 1.78 (dd, J = 19.1, 8.5 Hz, 2H), 1.67 (s, 3H), 1.56 (s, 4H), 1.42 (s, 2H), 1.27 (d, J = 17.4 Hz, 5H); ^{13}C NMR (100 MHz, $CDCl_3$) δ 176.4, 130.8, 130.4, 83.1, 79.3, 57.4, 56.7, 54.0, 50.1, 43.2, 37.4, 34.4, 29.0, 26.4, 24.9, 23.2, 22.8, 21.8; HRMS (ESI) m/z calcd. for $C_{20}H_{32}O_3N$ $[M+H]^+$ 334.23039, found 334.23825.

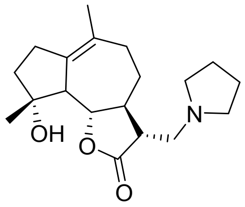

(3*R*,3a*S*,9*R*,9b*S*)-9-Hydroxy-6,9-dimethyl-3-(pyrrolidin-1-ylmethyl)-3a,4,5,7,8,9,9a,9b-octahydroazuleno[4,5-b] furan-2(3*H*)-one (5bb). According to the general procedure analogous to that described for **3aa**, except that the reaction was allowed for 5 hours. **5bb** (78 mg, 63%) was obtained from **4b** (100 mg, 0.3 mmol) as a yellow solid. Mp: 142-144 ^{o}C; $[\alpha]^{20}_{D}$= +20.2 (*c* 0.50, $CHCl_3$); ^{1}H NMR (500 MHz, $CDCl_3$) δ 3.80 (t, J = 10.3 Hz, 1H), 2.91 – 2.78 (m, 2H), 2.69 – 2.58 (m, 2H), 2.51 (d, J = 13.2 Hz, 4H), 2.38 (m, 2H), 2.26 – 2.02 (m, 4H), 1.84 – 1.70 (m, 6H), 1.67 (s, 3H), 1.28 (m, 4H); ^{13}C NMR (100 MHz, $CDCl_3$) δ 177.2, 131.8, 131.4, 84.2, 80.4, 58.4, 54.7, 54.3, 50.7, 45.7, 38.4, 35.5, 30.0, 27.4, 23.8, 23.7, 22.9; HRMS (ESI) m/z calcd. for $C_{19}H_{30}O_3N$ $[M+H]^+$ 320.21474, found 320.22211.

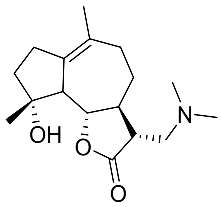

(3*R*,3a*S*,9*R*,9b*S*)-3-((Dimethylamino)methyl)-9-hydroxy-6,9-dimethyl-3a,4,5,7,8,9,9a,9b-octahydroazuleno[4,5-b] furan-2(3*H*)-one (5bd). According to the general procedure analogous to that described for **3aa**, except that the reaction was allowed for 5 hours. **5bd** (107 mg, 92%) was obtained from **4b** (120 mg, 0.4 mmol) as a yellow solid. Mp: 118-120 ^{o}C; $[\alpha]^{20}_{D}$= +15.6 (*c* 0.49, $CHCl_3$); ^{1}H NMR (500 MHz, $CDCl_3$) δ 3.82 (t, J = 10.2 Hz, 1H), 2.72 (dd, J = 12.7, 4.1 Hz, 1H), 2.68 – 2.54 (m, 3H), 2.38

(m, 2H), 2.26 (s, 6H), 2.16 (m, 4H), 2.08 – 1.98 (m, 1H), 1.77 (m, 2H), 1.69 (s, 3H), 1.30 (s, 3H); ^{13}C NMR (125 MHz, $CDCl_3$) δ 177.1, 131.8, 131.4, 84.1, 80.3, 58.3, 58.2, 50.9, 46.0, 44.6, 38.3, 35.3, 30.0, 27.3, 23.8, 22.8; HRMS (ESI) m/z calcd. for $C_{17}H_{28}O_3N$ $[M+H]^+$ 294.19909, found 294.20700.

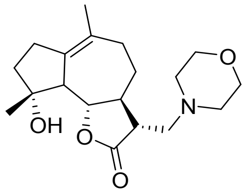

(3*R*,3a*S*,9*R*,9b*S*)-9-Hydroxy-6,9-dimethyl-3-(morpholinomethyl)-3a,4,5,7,8,9,9a,9b-octahydroazuleno[4,5-b] furan-2(3*H*)-one (5be). According to the general procedure analogous to that described for **3aa**, except that the reaction was allowed for 5 hours. **5be** (91 mg, 91%) was obtained from **4b** (100 mg, 0.3 mmol) as a yellow solid. Mp: 115-117 °C; $[\alpha]^{20}_{D}$= +20.6 (*c* 0.50, $CHCl_3$); ^{1}H NMR (400 MHz, $CDCl_3$) δ 3.82 (t, *J* = 10.2 Hz, 1H), 3.67 (s, 4H), 2.79 (d, *J* = 11.3 Hz, 1H), 2.71 – 2.56 (m, 3H), 2.54 – 2.31 (m, 6H), 2.16 (t, *J* = 12.8 Hz, 4H), 2.05 (d, *J* = 11.2 Hz, 1H), 1.78 (dd, *J* = 18.9, 8.3 Hz, 2H), 1.67 (s, 3H), 1.29 (s, 3H); ^{13}C NMR (100 MHz, $CDCl_3$) δ 176.7, 131.9, 131.4, 84.2, 80.4, 66.9, 58.4, 57.1, 54.2, 50.9, 44.2, 38.4, 35.4, 30.0, 27.4, 23.8, 22.9; HRMS (ESI) m/z calcd. for $C_{19}H_{30}O_4N$ $[M+H]^+$ 336.20966, found 336.21619.

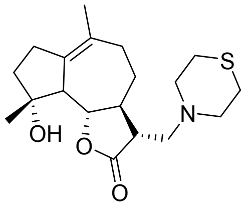

(3*R*,3a*S*,9*R*,9b*S*)-9-Hydroxy-6,9-dimethyl-3-(thiomorpholinomethyl)-3a,4,5,7,8,9,9a,9b-octahydroazuleno[4,5-b] furan-2(3*H*)-one (5bh). According to the general procedure analogous to that described for **3aa**, except that the reaction took 5 hours. **5bh** (86 mg, 82%) was obtained from **4b** (100 mg, 0.3 mmol) as a yellow solid. Mp: 96-100 °C; $[\alpha]^{20}_{D}$= +20.7 (*c* 0.52, $CHCl_3$); ^{1}H NMR (500 MHz, $CDCl_3$) δ 3.81 (t, *J* = 10.5 Hz, 1H), 2.83 (m, 1H), 2.75 (s, 4H), 2.64 (s, 5H), 2.59 (s, 1H), 2.38 (s, 2H), 2.17 (s, 3H), 2.10 (d, *J* = 14.0 Hz, 1H), 2.02 (d, *J* = 11.5 Hz, 1H), 1.84 – 1.73 (m, 2H),

1.68 (s, 3H), 1.29 (s, 5H); ^{13}C NMR (100 MHz, CDCl$_3$) δ 177.5, 132.3, 131.8, 84.6, 80.8, 58.9, 57.7, 56.1, 51.2, 45.1, 38.9, 35.9, 30.5, 28.3, 27.8, 24.3, 23.3; HRMS (ESI) m/z calcd. for $C_{19}H_{30}O_3NS$ [M+H]$^+$ 352.18681, found 352.19336.

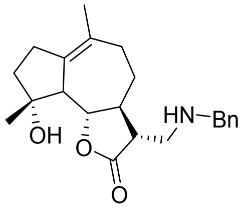

(3*R*,3a*S*,9*R*,9b*S*)-3-((Benzylamino)methyl)-9-hydroxy-6,9-dimethyl-3a,4,5,7,8,9,9a,9b-octahydroazuleno[4,5-b] furan-2(3*H*)-one (5bi). According to the general procedure analogous to that described for **3aa**, except that the reaction took 20h. **5bi** (26 mg, 36%) was obtained from **4b** (58 mg, 0.2 mmol) as a yellow oil. $[\alpha]^{20}_D$= –16.5 (*c* 0.22, CHCl$_3$); ^{1}H NMR (500 MHz, CDCl$_3$) δ 7.33 (m, 5H), 3.83 (m, 3H), 2.96 (dd, *J* = 12.2, 3.8 Hz, 1H), 2.77 (dd, *J* = 12.2, 6.7 Hz, 1H), 2.65 (d, *J* = 10.0 Hz, 1H), 2.49 – 2.33 (m, 3H), 2.25 – 2.06 (m, 3H), 1.77 (m, 3H), 1.67 (s, 3H), 1.28 (d, *J* = 10.0 Hz, 5H); ^{13}C NMR (125 MHz, CDCl$_3$) δ 177.5, 139.7, 131.9, 131.3, 128.5, 128.1, 127.1, 84.5, 80.3, 58.2, 53.9, 48.8, 46.6, 46.0, 38.4, 35.2, 30.0, 27.0, 23.8, 22.8; HRMS (ESI) m/z calcd. for $C_{22}H_{30}O_3N$ [M+H]$^+$ 356.21474, found 356.22107.

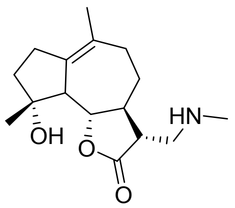

(3*R*,3a*S*,9*R*,9b*S*)-9-Hydroxy-6,9-dimethyl-3-((methylamino)methyl)-3a,4,5,7,8,9,9a,9b-octahydroazuleno[4,5-b] furan-2(3*H*)-one (5bf). According to the general procedure analogous to that described for **3aa**, except that the reaction was allowed for 15 hours. **5bf** (27 mg, 49%) was obtained from **4b** (60 mg, 0.2 mmol) as a white solid. Mp: 110-112 °C; $[\alpha]^{20}_D$= –13.8 (*c* 0.23, CHCl$_3$); ^{1}H NMR (500 MHz, CDCl$_3$) δ 3.86 (t, *J* = 10.3 Hz, 1H), 2.91 (dd, *J* = 12.0, 4.0 Hz, 1H), 2.80 (dd, *J* = 12.1, 7.2 Hz, 1H), 2.66 (d, *J* = 10.4 Hz, 1H), 2.53 – 2.45 (m, 4H), 2.37 (d, *J* = 8.9 Hz, 1H), 2.24 – 2.11 (m, 4H), 2.12 – 1.99 (m, 2H), 1.92 (dd, *J* = 13.8, 2.2 Hz,

1H), 1.79 (dd, J = 18.4, 7.7 Hz, 2H), 1.68 (s, 3H), 1.35 – 1.26 (m, 3H); ^{13}C NMR (125 MHz, $CDCl_3$) δ 177.7, 132.1, 131.5, 84.7, 80.5, 58.4, 49.5, 49.3, 46.6, 38.6, 37.0, 35.4, 30.2, 27.3, 24.0, 23.0; HRMS (ESI) m/z calcd. for $C_{16}H_{26}O_3N$ $[M+H]^+$ 280.18344, found 280.19034.

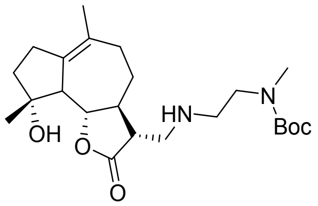

***tert*-Butyl (2-((((3*R*,3a*S*,9*R*,9b*S*)-9-Hydroxy-6,9-dimethyl-2-oxo-2,3,3a,4,5,7,8,9,9a,9b-decahydroazuleno[4,5-b]furan-3-yl)methyl)amino)ethyl)(methyl)carbamate (5bj).** According to the general procedure analogous to that described for **3aa**, except that the reaction was allowed for 15 hours. **5bj** (88 mg, 70%) was obtained from **4b** (100 mg, 0.3 mmol) as a white solid. Mp: 120-122 °C; $[\alpha]^{20}_D$= –10.0 (*c* 0.48, $CHCl_3$); ^{1}H NMR (500 MHz, $CDCl_3$) δ 3.98 – 3.78 (m, 1H), 3.37 (s, 2H), 3.09 – 2.97 (m, 1H), 2.92 (s, 3H), 2.82 (s, 2H), 2.69 (d, J = 9.7 Hz, 1H), 2.50 – 2.38 (m, 2H), 2.22 (s, 3H), 2.15 – 2.05 (m, 2H), 1.96 (d, J = 12.8 Hz, 1H), 1.81 (tt, J = 22.7, 11.3 Hz, 3H), 1.72 (s, 3H), 1.50 (s, 9H), 1.41 – 1.26 (m, 4H); ^{13}C NMR (100 MHz, $CDCl_3$) δ 177.3, 155.9, 132.0, 131.2, 84.5, 80.3, 79.5, 60.4, 58.3, 48.9, 46.8, 38.4, 35.3, 30.1, 28.5, 27.2, 23.8, 22.9, 21.1, 14.2; HRMS (ESI) m/z calcd. for $C_{23}H_{39}O_5N_2$ $[M+H]^+$ 423.27807, found 423.28516.

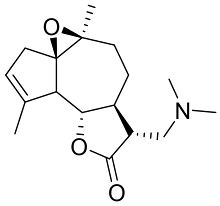

(3a*R*,4a*S*,6a*S*,7*R*,9a*S*)-7-((Dimethylamino)methyl)-1,4a-dimethyl-5,6,6a,7,9a,9b-hexahydro-3*H*-oxireno[2',3':8,8a] azuleno[4,5-b]furan-8(4a*H*)-one (5cd). According to the general procedure analogous to that described for **3aa**, except that the reaction took 5h. **5cd** (58 mg, 99%) was obtained from **4c** (60 mg, 0.2 mmol) as a white solid. Mp: 84-86 °C; $[\alpha]^{20}_D$= +72.5 (*c* 0.49, $CHCl_3$); ^{1}H NMR (500 MHz, $CDCl_3$) δ 5.55 (s, 1H), 4.02 (d, J = 9.5 Hz, 1H), 2.84 (d, J = 9.4 Hz, 1H), 2.73 (m, 2H), 2.58 (s, 1H), 2.31

(s, 1H), 2.24 (s, 5H), 2.17 – 2.04 (m, 2H), 2.02 – 1.83 (m, 6H), 1.63 (d, *J* = 10.6 Hz, 1H), 1.53 – 1.40 (m, 1H), 1.29 (d, *J* = 37.0 Hz, 3H); ^{13}C NMR (100 MHz, $CDCl_3$) *δ* 177.8, 140.7, 124.8, 82.6, 72.5, 62.7, 58.0, 52.4, 51.9, 46.1, 44.6, 39.6, 33.7, 22.9, 22.8, 18.3; HRMS (ESI) m/z calcd. for $C_{17}H_{26}O_3N$ $[M+H]^+$ 292.18344, found 292.19095.

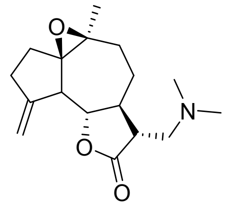

(3a*R*,4a*S*,6a*S*,7*R*,9a*S*)-7-((Dimethylamino)methyl)-4a-methyl-1-methyleneoctahydro-1H-oxireno[2',3':8,8a]azuleno[4,5-b]furan-8(4a*H*)-one (5dd). According to the general procedure analogous to that described for **3aa**, except that the reaction was allowed for 5 hours. **5dd** (40 mg, 70%) was obtained from **4d** (60 mg, 0.2 mmol) as a brown oil. $[\alpha]^{20}_D$= +40.9 (*c* 0.18, $CHCl_3$); ^1H NMR (400 MHz, $CDCl_3$) *δ* 5.40 (s, 1H), 5.12 (s, 1H), 4.00 (t, *J* = 10.1 Hz, 1H), 2.85 (d, *J* = 9.8 Hz, 1H), 2.72 (s, 1H), 2.67 – 2.53 (m, 2H), 2.51 – 2.40 (m, 1H), 2.28 (s, 6H), 2.13 (m, 2H), 2.06 – 1.98 (m, 1H), 1.93 (m, 1H), 1.78 – 1.61 (m, 3H), 1.54 – 1.40 (m, 1H), 1.40 – 1.31 (m, 3H); ^{13}C NMR (100 MHz, $CDCl_3$) *δ* 177.5, 148.3, 110.8, 82.1, 73.2, 63.3, 57.9, 50.6, 49.2, 46.1, 44.7, 34.3, 32.5, 31.2, 23.0, 23.0; HRMS (ESI) m/z calcd. for $C_{17}H_{26}O_3N$ $[M+H]^+$ 292.18344, found 292.19116.

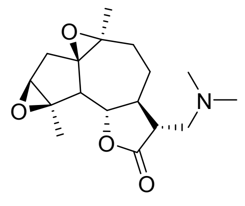

(1a*S*,2a*R*,3a*S*,5a*S*,6*R*,8a*S*,8c*R*)-6-((dimethylamino)methyl)-3a,8c-dimethyloctahydro-2*H*-bis(oxireno)[2',3':2,3;2'',3'':8,8a]azuleno[4,5-b]furan-7(3a*H*)-one (5ed). According to the general procedure analogous to that described for **3aa**, except that the reaction was allowed for 18 hours. **5ed** (46 mg, 61%) was obtained from **4e** (75 mg, 0.25 mmol) as a white solid. Mp: 162-164 °C; $[\alpha]^{20}_D$= +55.5 (*c* 0.48, $CHCl_3$); ^1H NMR (500 MHz, DMSO-*d*$_6$) *δ* 3.95 (t, *J* = 10.3 Hz, 1H), 3.32 (s, 1H), 3.29 (s, 3H), 2.60 –

2.53 (m, 2H), 2.34 (d, J = 15.3 Hz, 1H), 2.13 (d, J = 13.5 Hz, 6H), 1.93 (m, 1H), 1.83 (dd, J = 24.5, 11.7 Hz, 2H), 1.67 (m, 2H), 1.49 (s, 3H), 1.20 (s, 3H); ^{13}C NMR (126 MHz, DMSO-d_6) δ 177.9, 80.5, 69.7, 65.5, 62.3, 59.5, 58.7, 51.4, 49.2, 46.4, 44.0, 36.5, 33.2, 23.2, 22.8, 19.2; HRMS (ESI) m/z calcd. for $C_{17}H_{26}O_4N$ [M+H]$^+$ 308.17836, found 308.18463.

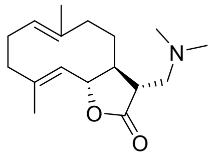

(3a*S*,6*Z*,10*Z*,11a*S*)-3-((Dimethylamino)methyl)-6,10-dimethyl-3a,4,5,8,9,11a-hexahydrocyclodeca[b]furan-2(3*H*)-one (5fd). According to the general procedure analogous to that described for **3aa**, except that the reaction was allowed for 5 hours. **5fd** (33 mg, 60%) was obtained from **4f** (60 mg, 0.2 mmol) as a yellow solid. Mp: 101-103 °C; $[\alpha]^{20}_{D}$= +0.84 (c 0.47, CHCl$_3$); ^{1}H NMR (500 MHz, CDCl$_3$) δ 4.83 (d, J = 9.9 Hz, 1H), 4.67 (d, J = 9.7 Hz, 1H), 4.56 (d, J = 9.5 Hz, 1H), 2.73 (d, J = 4.7 Hz, 1H), 2.61 (d, J = 4.5 Hz, 1H), 2.35 (d, J = 6.4 Hz, 2H), 2.26 (d, J = 13.4 Hz, 8H), 2.18 (s, 1H), 2.12 – 2.06 (m, 2H), 2.01 (m, 2H), 1.69 (s, 3H), 1.62 (d, J = 11.5 Hz, 1H), 1.41 (s, 3H); ^{13}C NMR (100 MHz, CDCl$_3$) δ 177.6, 140.5, 137.1, 127.4, 127.0, 81.4, 58.1, 51.2, 46.2, 46.1, 41.1, 39.6, 28.4, 26.2, 17.3, 16.2; HRMS (ESI) m/z calcd. for $C_{17}H_{28}O_2N$ [M+H]$^+$ 278.20418, found 278.21207.

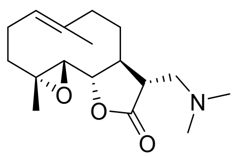

(3*R*,3a*S*,9a*R*,10a*S*,10b*S*,*E*)-3-((Dimethylamino)methyl)-6,9a-dimethyl-3a,4,5,8,9,9a,10a,10b-octahydrooxireno[2',3':9,10]cyclodeca[1,2-b]furan-2(3*H*)-one (5gd). According to the general procedure analogous to that described for **3aa**, except that the reaction was allowed for 5 hours. **5fd** (30 mg, 48%) was obtained from **4g** (60 mg, 0.2 mmol) as a yellow solid. Mp: 128-130 °C; $[\alpha]^{20}_{D}$= –21.2 (c 0.53, CHCl$_3$); ^{1}H NMR (500 MHz, CDCl$_3$) δ 5.17 (d, J = 11.5 Hz, 1H), 3.80 (t, J = 8.9 Hz, 1H), 2.76 – 2.67 (m, 2H), 2.60 (dd, J =

13.1, 4.2 Hz, 1H), 2.37 (dq, J = 10.2, 5.2 Hz, 2H), 2.21 (d, J = 19.6 Hz, 8H), 2.18 – 1.99 (m, 4H), 1.67 (s, 3H), 1.64 – 1.57 (m, 1H), 1.27 (s, 3H), 1.19 (m, 1H); ^{13}C NMR (100 MHz, $CDCl_3$) δ 176.5, 134.7, 125.0, 82.1, 66.5, 61.5, 57.7, 47.9, 46.5, 46.2, 41.1, 36.7, 29.9, 24.1, 17.2, 17.0; HRMS (ESI) m/z calcd. for $C_{17}H_{28}O_3N$ $[M+H]^+$ 294.19909, found 294.20697.

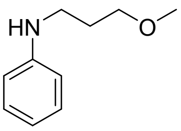

***N*-(3-Methoxypropyl) aniline (6a).** [1] **6a** was prepared according to the literature procedures as a yellow oil. ^{1}H NMR (400 MHz, Acetone-d_6) δ 7.13 – 6.98 (m, 2H), 6.68 – 6.49 (m, 3H), 3.46 (t, J = 6.1 Hz, 2H), 3.28 (s, 3H), 3.16 (t, J = 6.2 Hz, 2H), 1.84 (m, 2H).

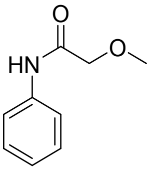

2-Methoxy-*N*-phenylacetamide (7a). yellow oil. According to the general procedure analogous to that described for **1a.** ^{1}H NMR (400 MHz, $CDCl_3$) δ 8.26 (s, 1H), 7.57 (m, 2H), 7.33 (m, 2H), 7.12 (t, J = 7.4 Hz, 1H), 4.00 (s, 2H), 3.49 (s, 3H).

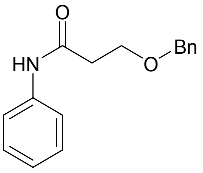

3-(Benzyloxy)-*N*-phenylpropanamide (8a). colorless oil. According to the general procedure analogous to that described for **1a**. ^{1}H NMR (400 MHz, $CDCl_3$) δ 8.31 (s, 1H), 7.44 (d, J = 7.8 Hz, 2H), 7.38 – 7.26 (m, 7H), 7.08 (t, J = 7.4 Hz, 1H), 4.60 (s, 2H), 3.84 (t, J = 5.6 Hz, 2H), 2.66 (t, J = 5.6 Hz, 2H).

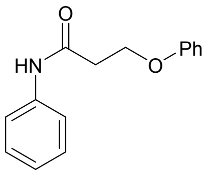

3-Phenoxy-*N*-phenylpropanamide (8b). a yellow oil. According to the general procedure analogous to that described for **1a.** ^{1}H NMR (400 MHz, DMSO-d_6) δ 10.07 (s, 1H), 7.62 (d, J = 8.4 Hz, 2H), 7.39 – 7.22 (m, 4H), 7.06 (d, J = 7.4 Hz, 1H), 6.94 (t, J = 8.1 Hz, 3H), 4.27 (t, J = 6.0 Hz, 2H), 2.80 (t, J = 6.0 Hz, 2H).

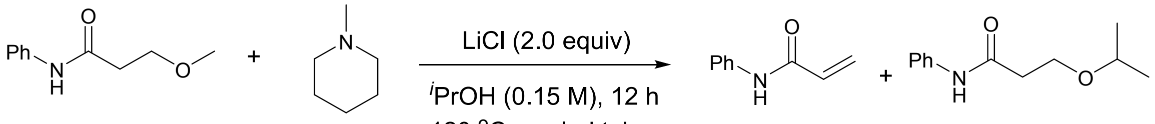

1a **2j** (2.0 equiv) **9a**, 27% **10a**, 21%

According to the general procedure analogous to that described for **3aa**, except that the reaction was allowed for 12 hours. **9a** (32 mg, 27%) and **10a** (35 mg, 21%) and raw material **1a** (39mg, 26%) was obtained from **1a** (150 mg, 0.8 mmol).

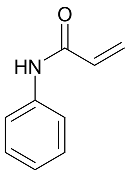

***N*-phenylacrylamide (9a).** white solid. Mp: 104-106 °C; ^{1}H NMR (400 MHz,) δ 7.58 (d, J = 7.5 Hz, 2H), 7.33 (t, J = 7.9 Hz, 2H), 7.13 (t, J = 7.3 Hz, 1H), 6.44 (d, J = 16.5 Hz, 1H), 6.26 (m, 1H), 5.77 (m, 1H). ^{13}C NMR (100 MHz, $CDCl_3$) δ 163.5, 137.7, 131.2, 129.1, 127.86, 124.6, 120.0; HRMS (ESI) m/z calcd. for $C_9H_{10}ON$ $[M+H]^+$ 148.06841, found 148.07492.

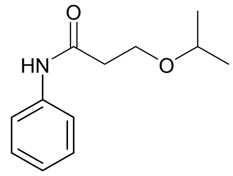

3-Isopropoxy-*N*-phenylpropanamide (10a). colorless oil. ^{1}H NMR (400 MHz, $CDCl_3$) δ 8.67 (s, 1H), 7.51 (dd, J = 8.5, 0.9 Hz, 2H), 7.35 – 7.27 (m, 2H), 7.15 – 7.01 (m, 1H), 3.78 – 3.64 (m, 3H), 2.74 – 2.50 (m, 2H), 1.24 (d, J = 6.1 Hz, 6H); ^{13}C NMR (100 MHz, $CDCl_3$) δ 170.2, 138.3, 129.0, 124.0, 119.6, 72.4, 64.1, 38.2, 22.1; HRMS (ESI) m/z calcd. for $C_{12}H_{18}O_2N$ $[M+H]^+$ 208.12593, found 208.13239.

References

1. Joel A. Bergman, Karrune Woan, Patricio Perez-Villarroel, Alejandro Villagra, Eduardo M. Sotomayor, and Alan P. Kozikowski, *J. Med. Chem.* 2012, 55, 9891−9899.

3. NMR spectra

^{1}H NMR spectra of **1a**

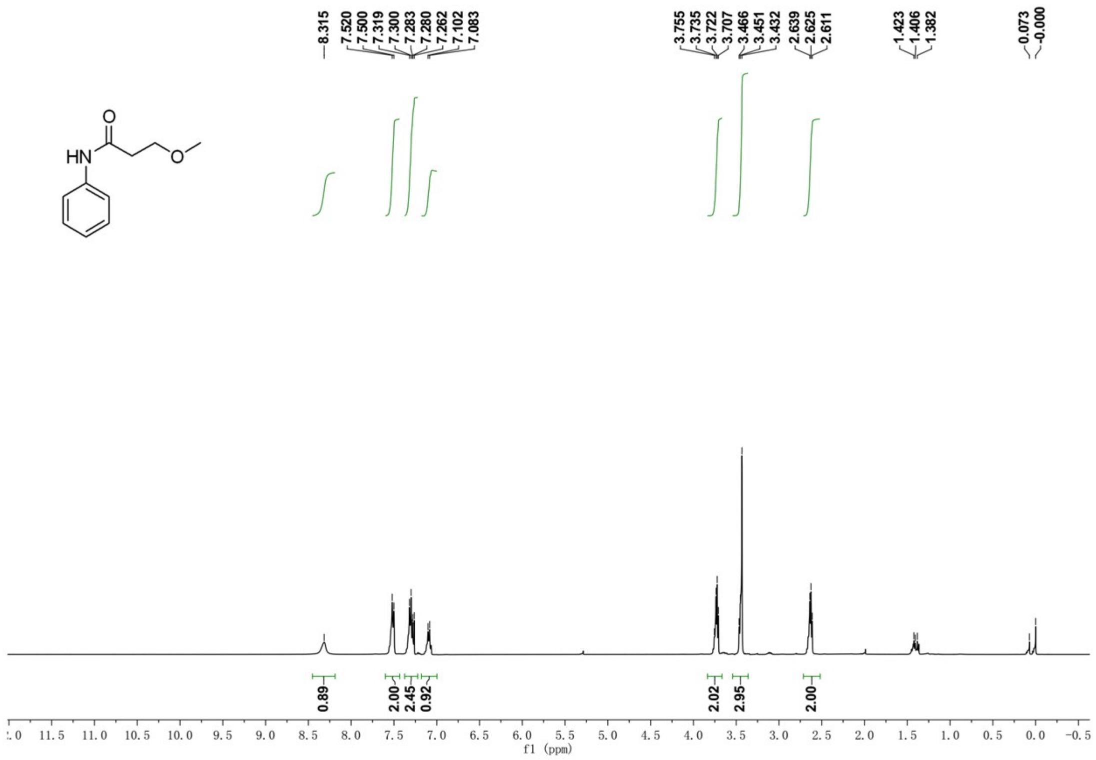

^{1}H NMR spectra of **1b**

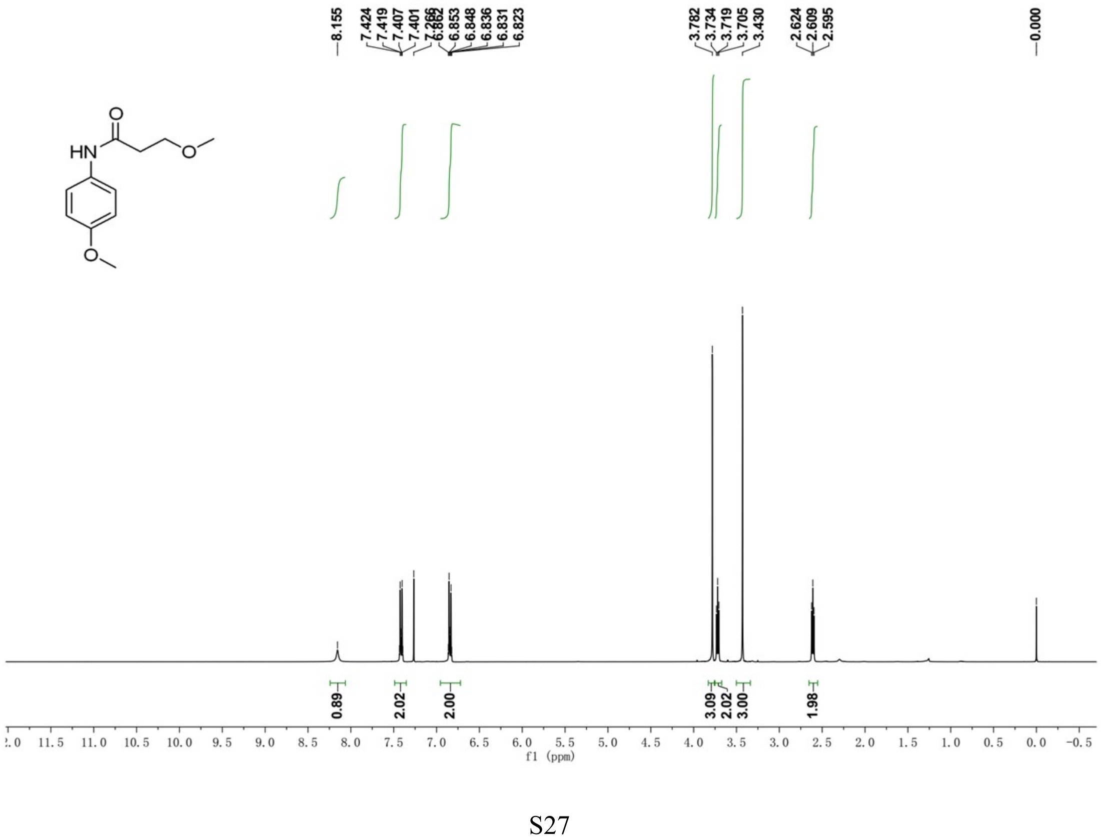

^{13}C NMR spectra of **1b**

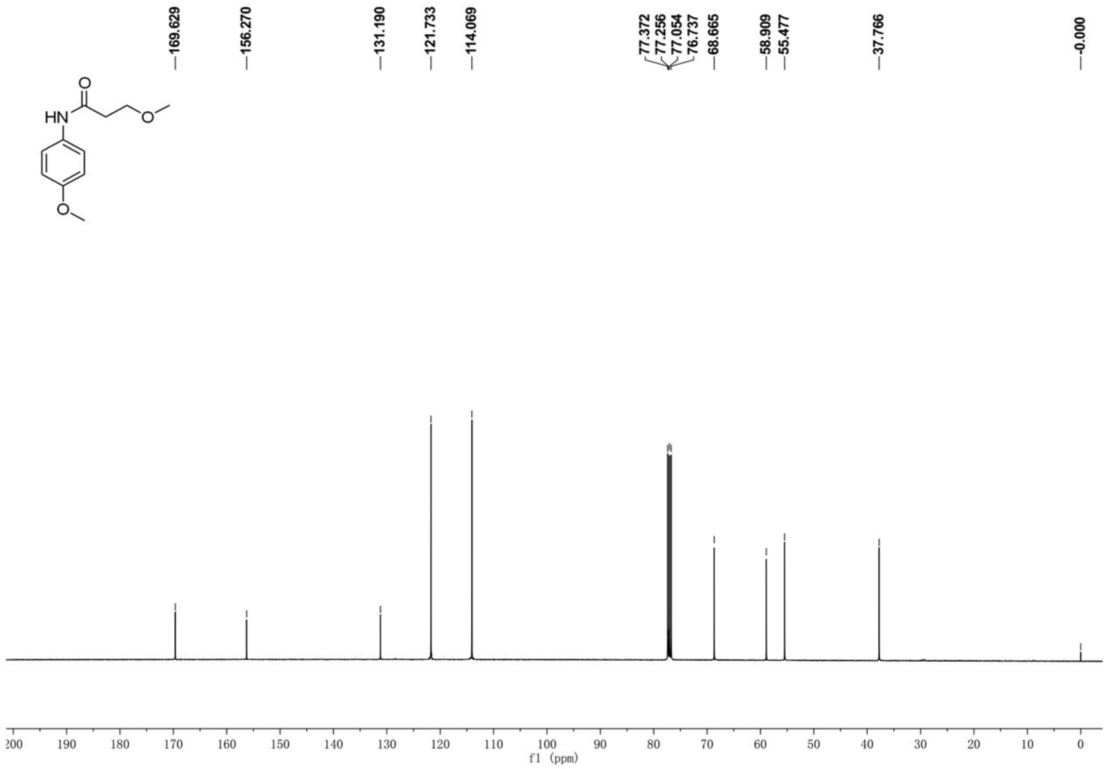

^{1}H NMR spectra of **1c**

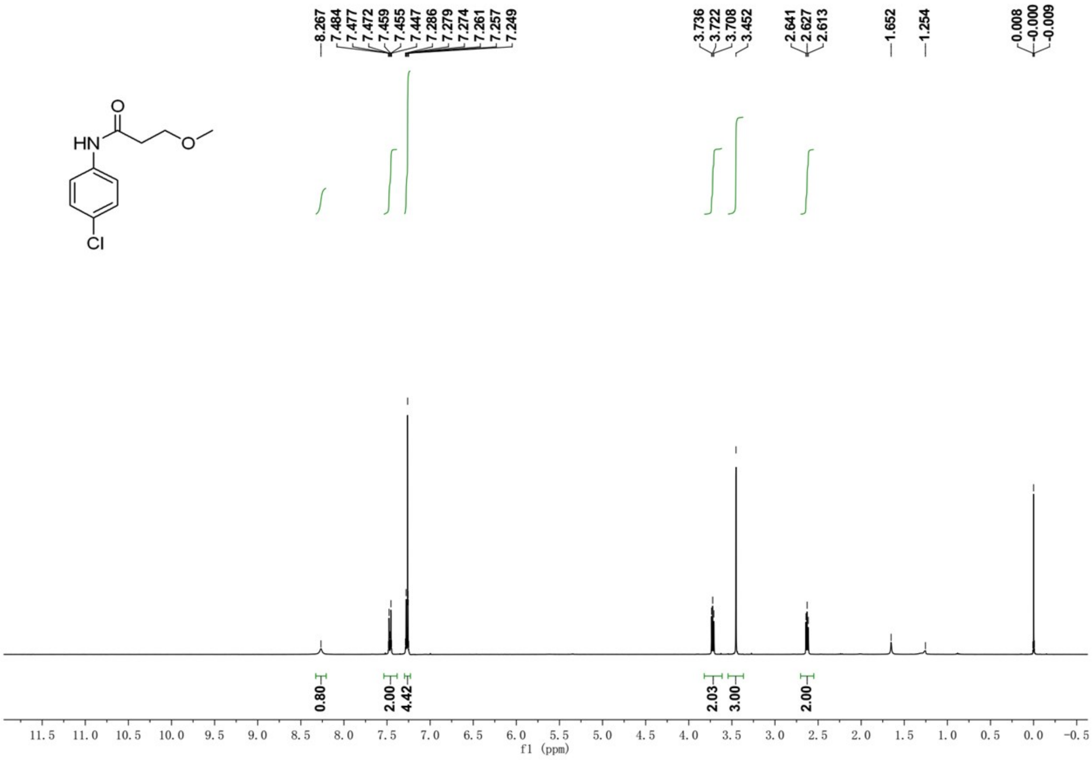

^{13}C NMR spectra of **1c**

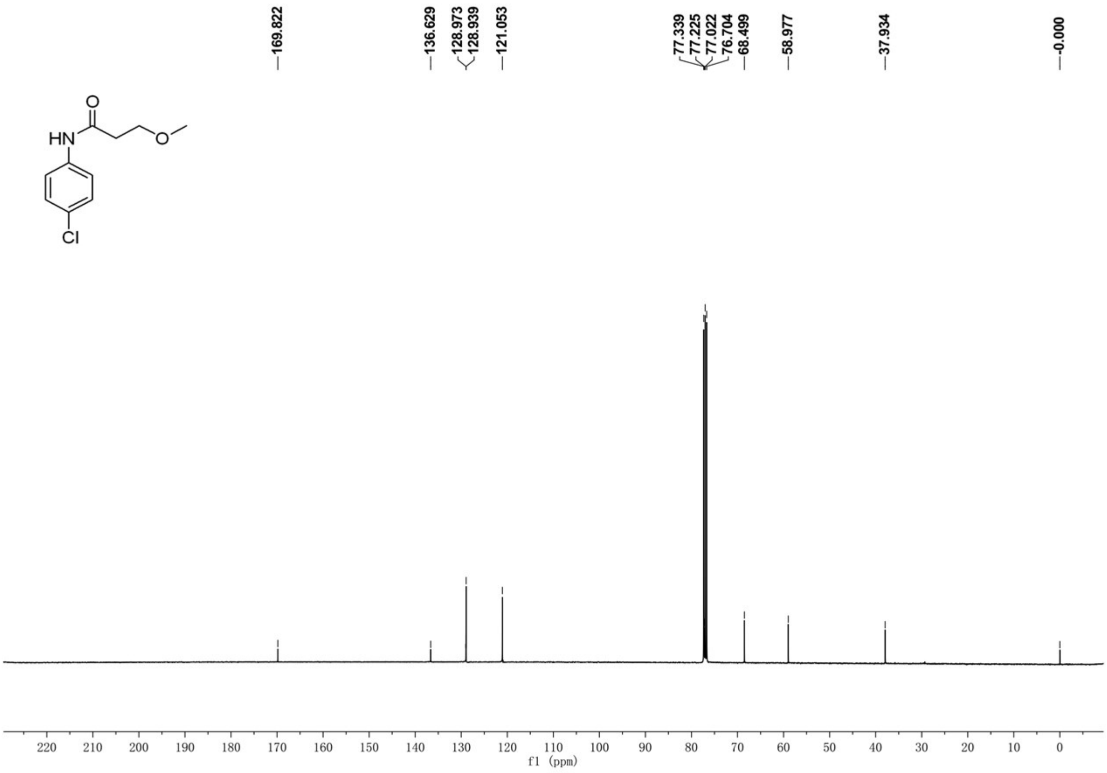

^{1}H NMR spectra of **1d**

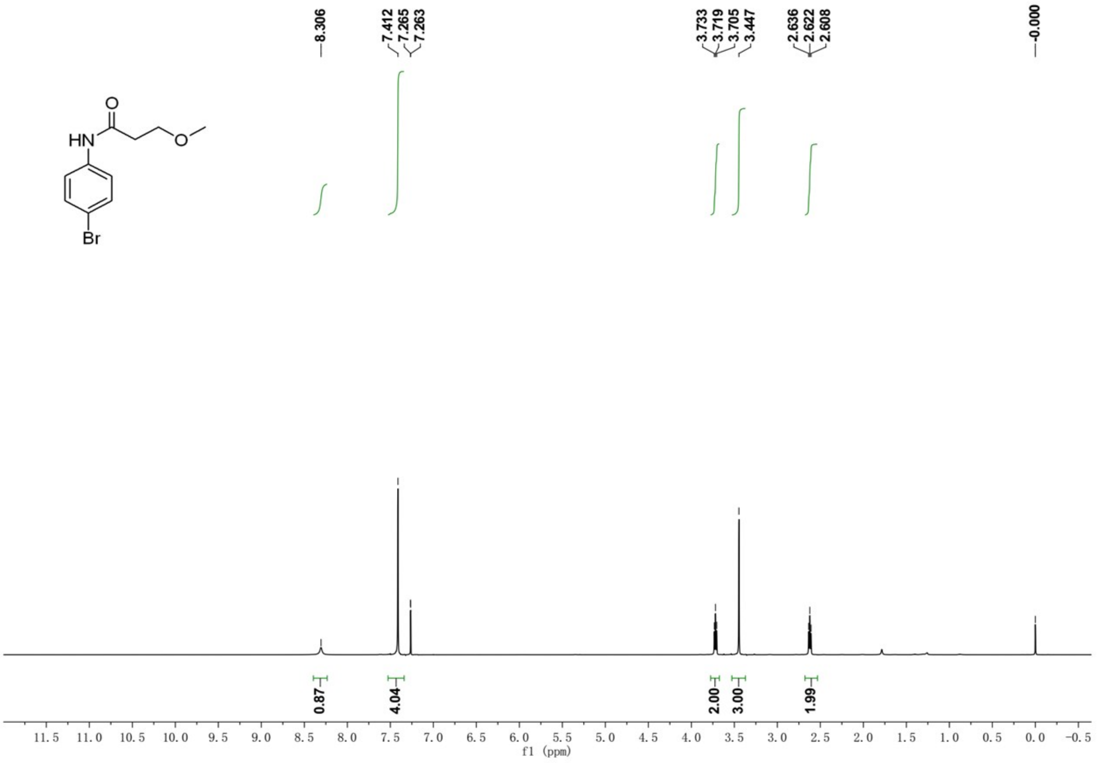

^{1}H NMR spectra of **1e**

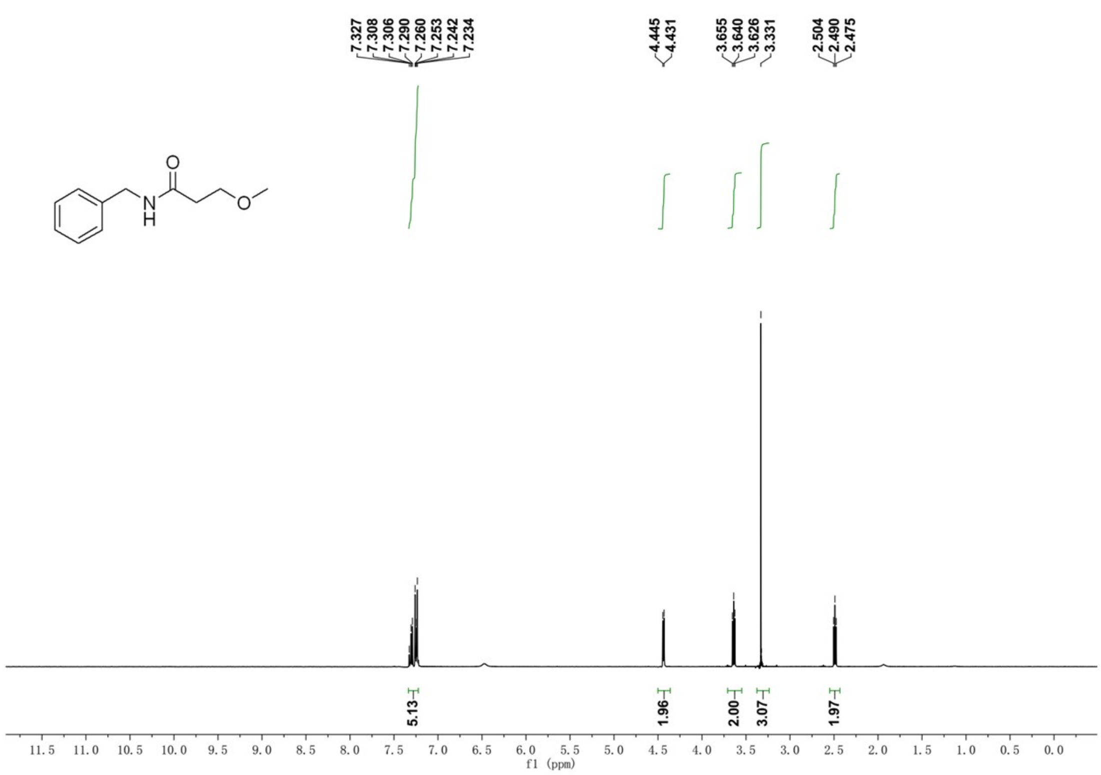

^{1}H NMR spectra of **1f**

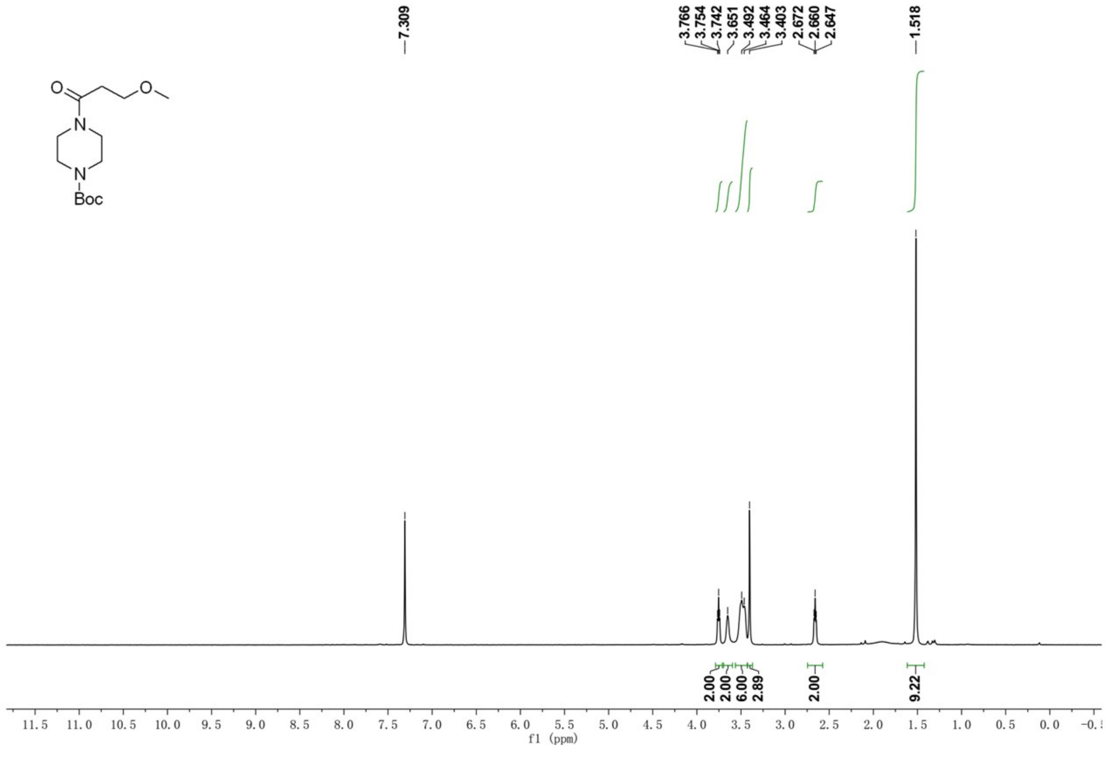

^{1}H NMR spectra of **3aa**

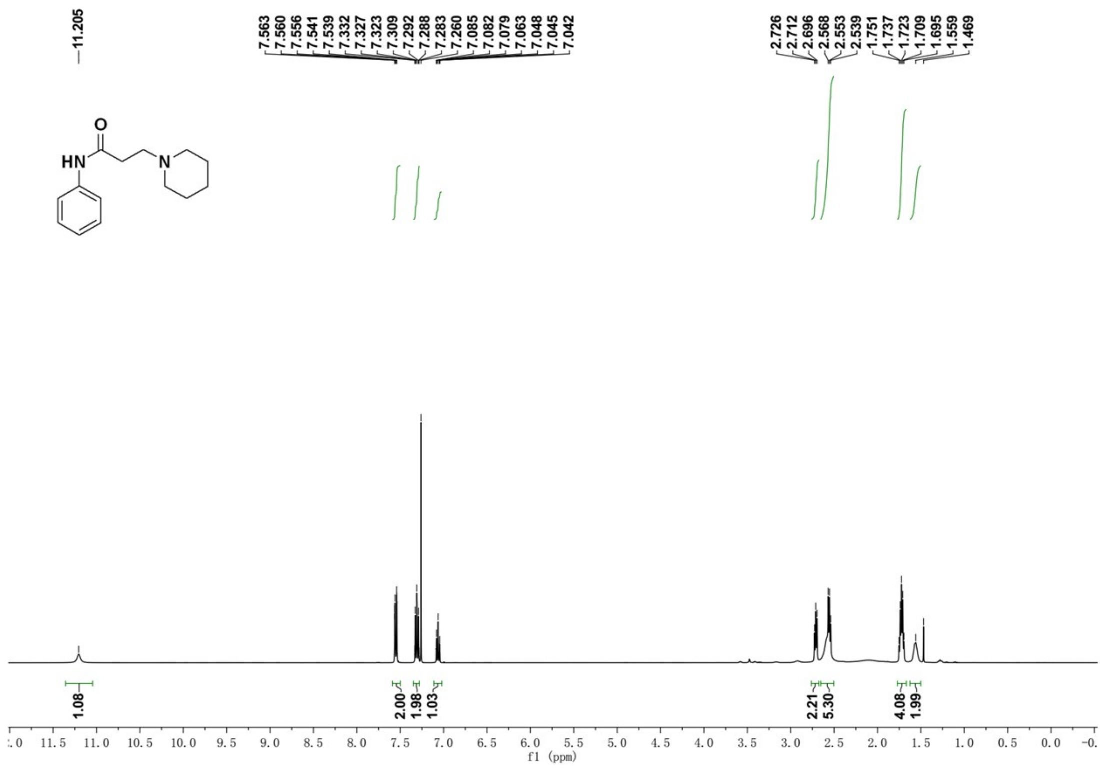

^{13}C NMR spectra of **3aa**

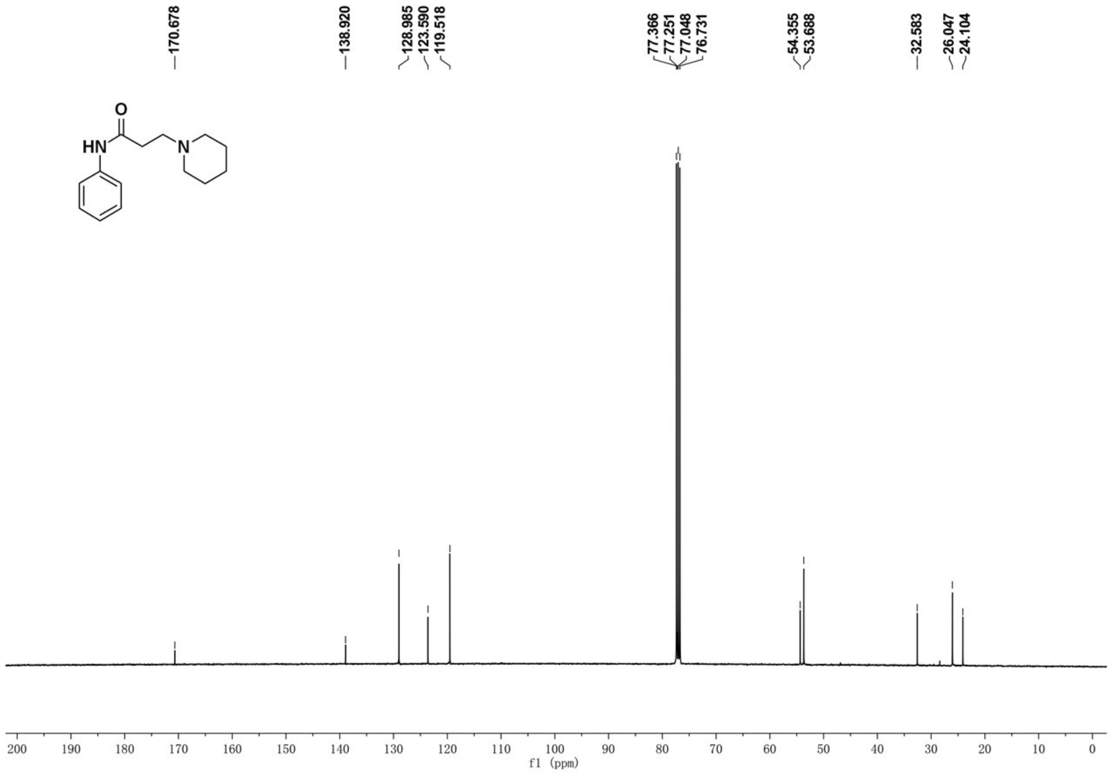

^{1}H NMR spectra of **3ba**

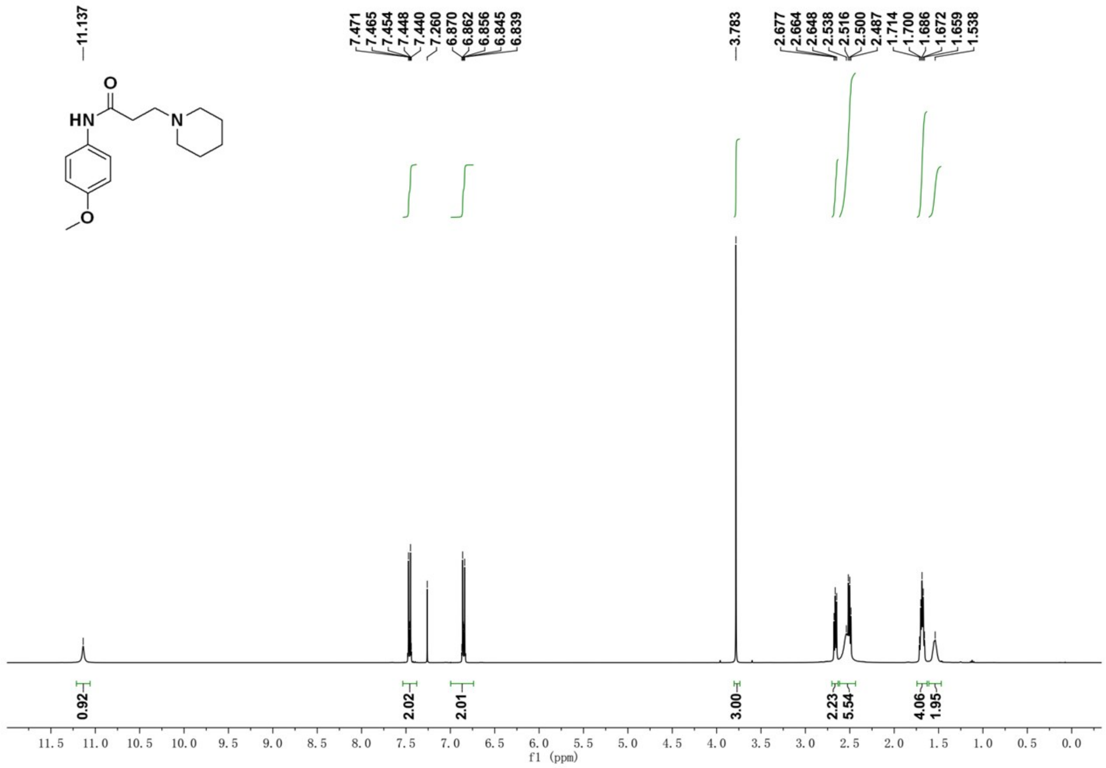

^{13}C NMR spectra of **3ba**

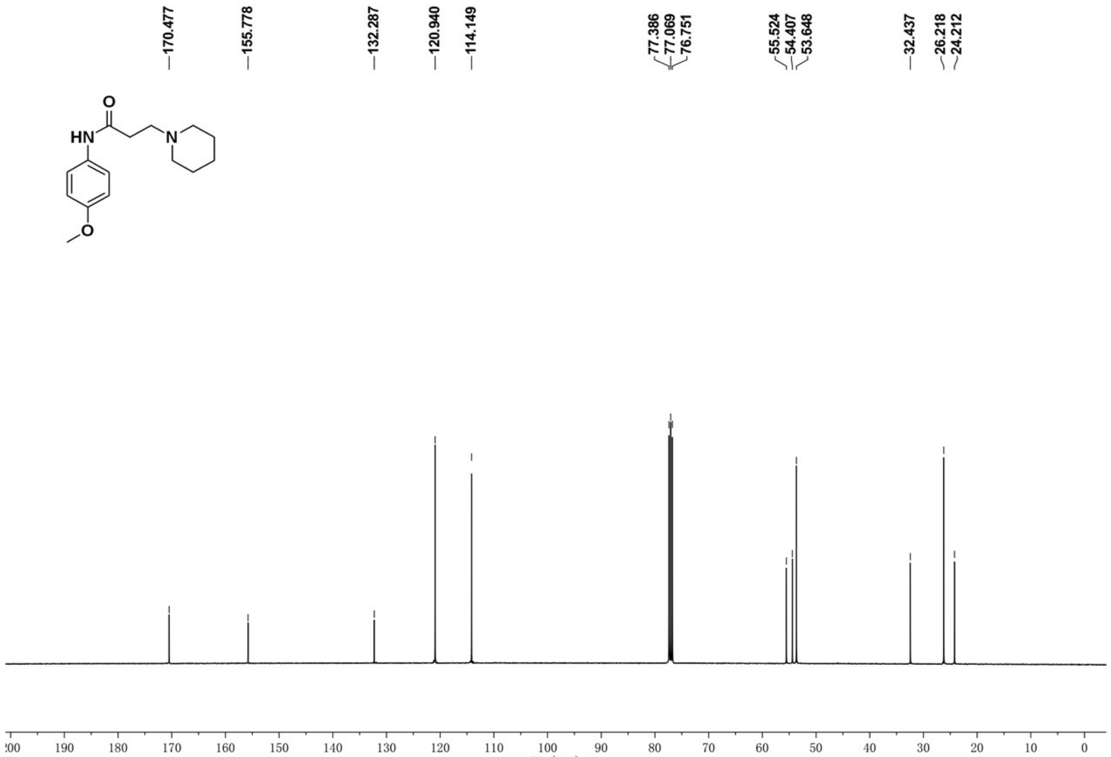

^{1}H NMR spectra of **3ca**

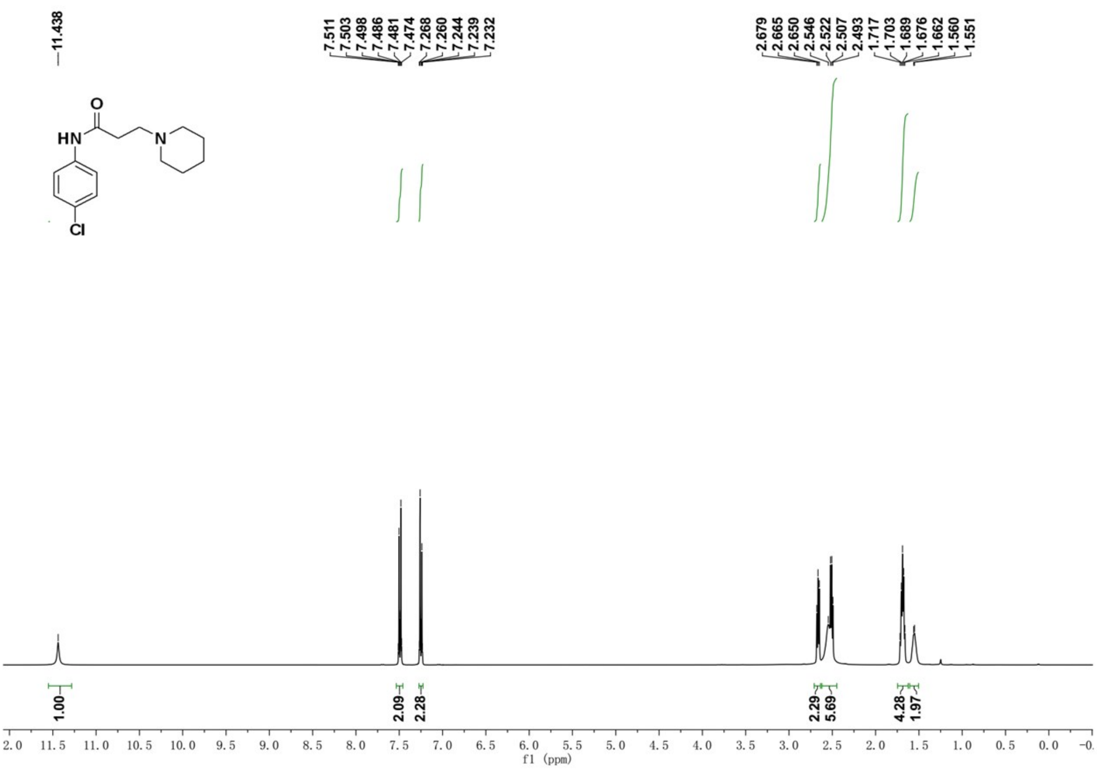

^{13}C NMR spectra of **3ca**

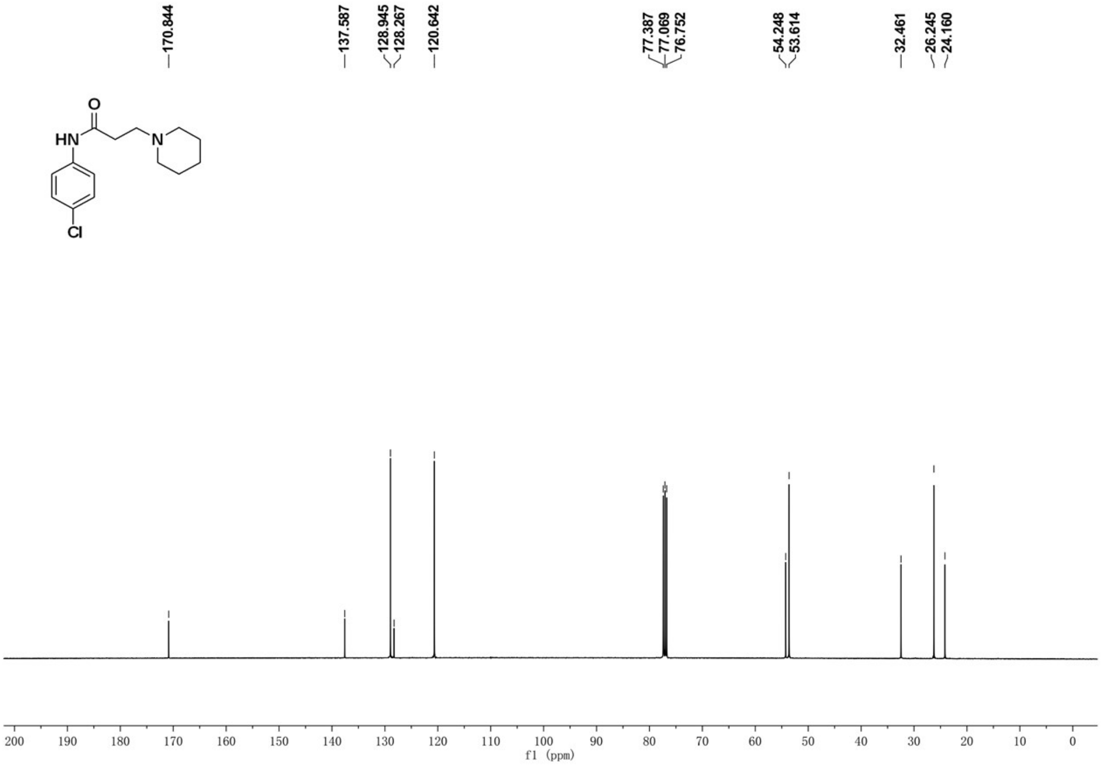

^{1}H NMR spectra of **3da**

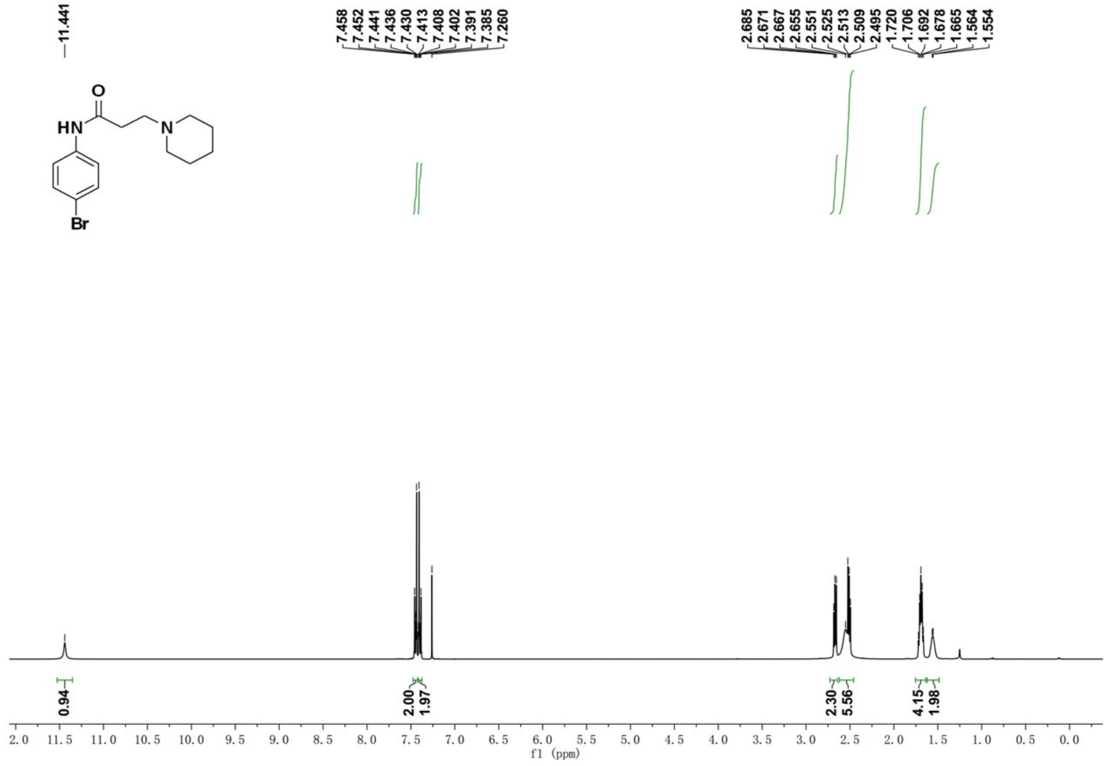

^{13}C NMR spectra of **3da**

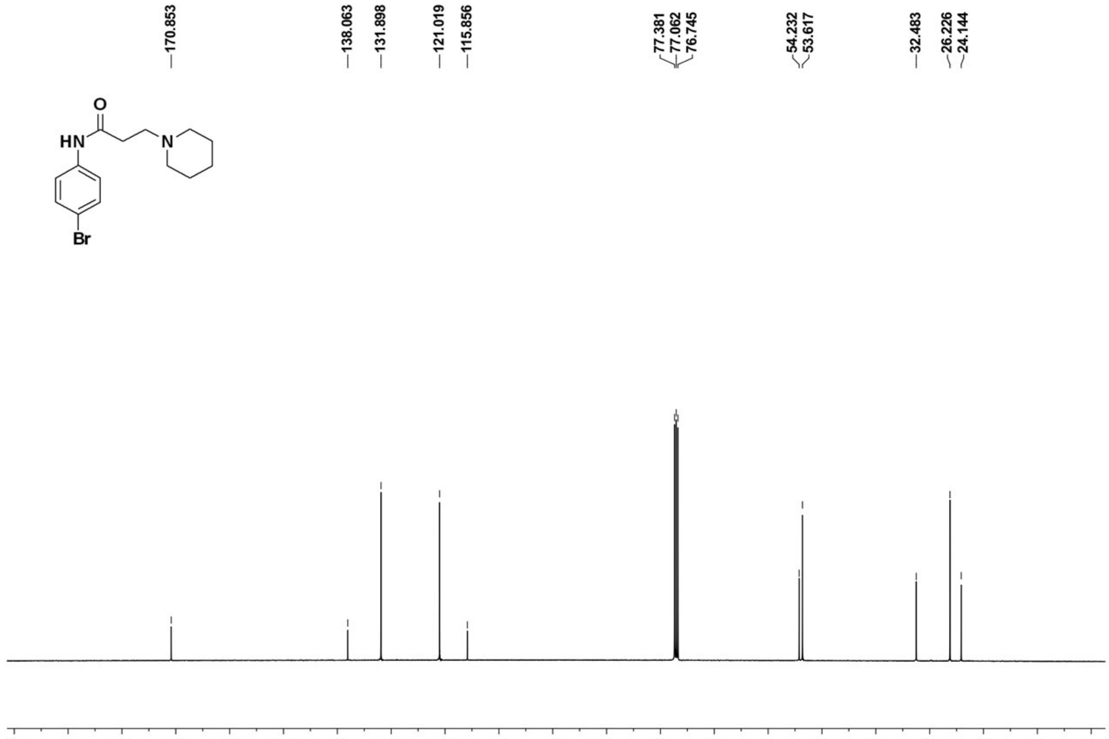

^{1}H NMR spectra of **3ab**

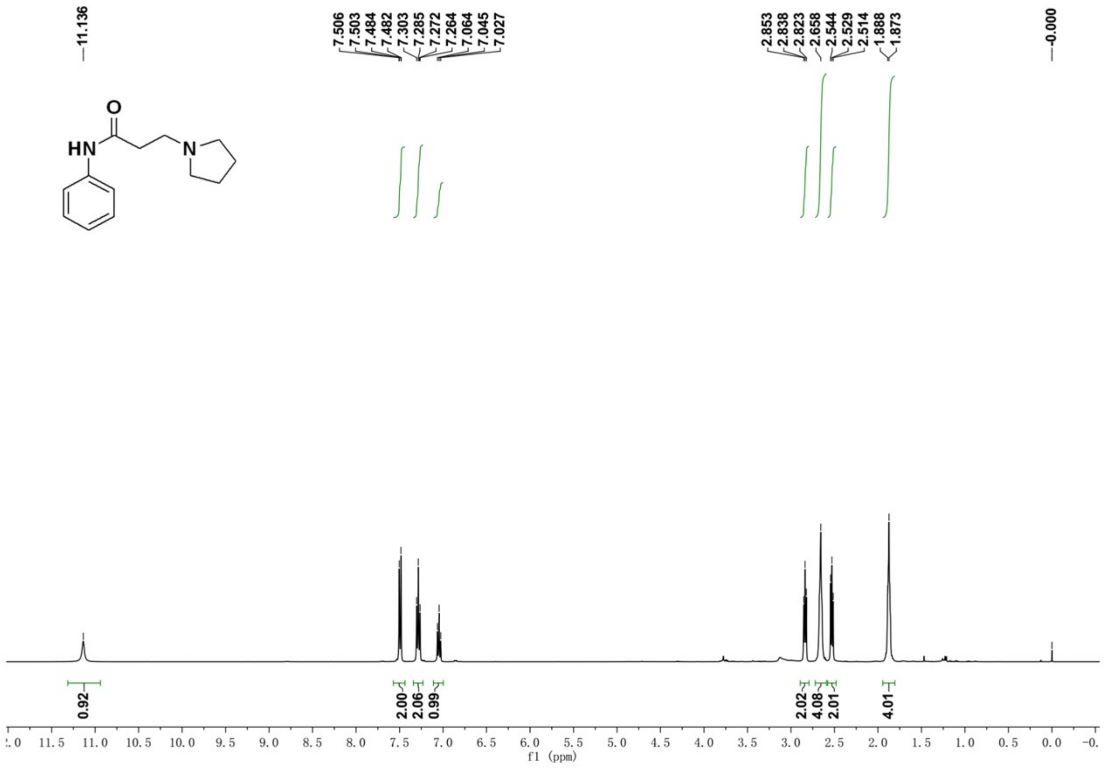

^{13}C NMR spectra of **3ab**

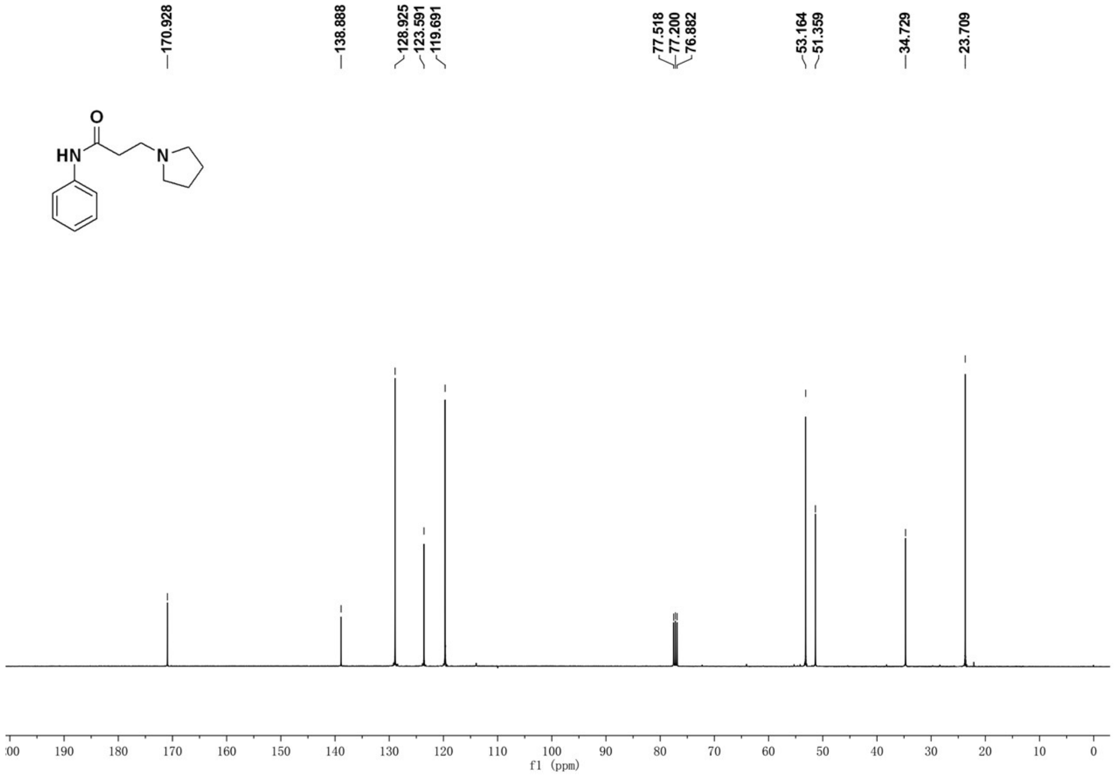

^{1}H NMR spectra of **3bb**

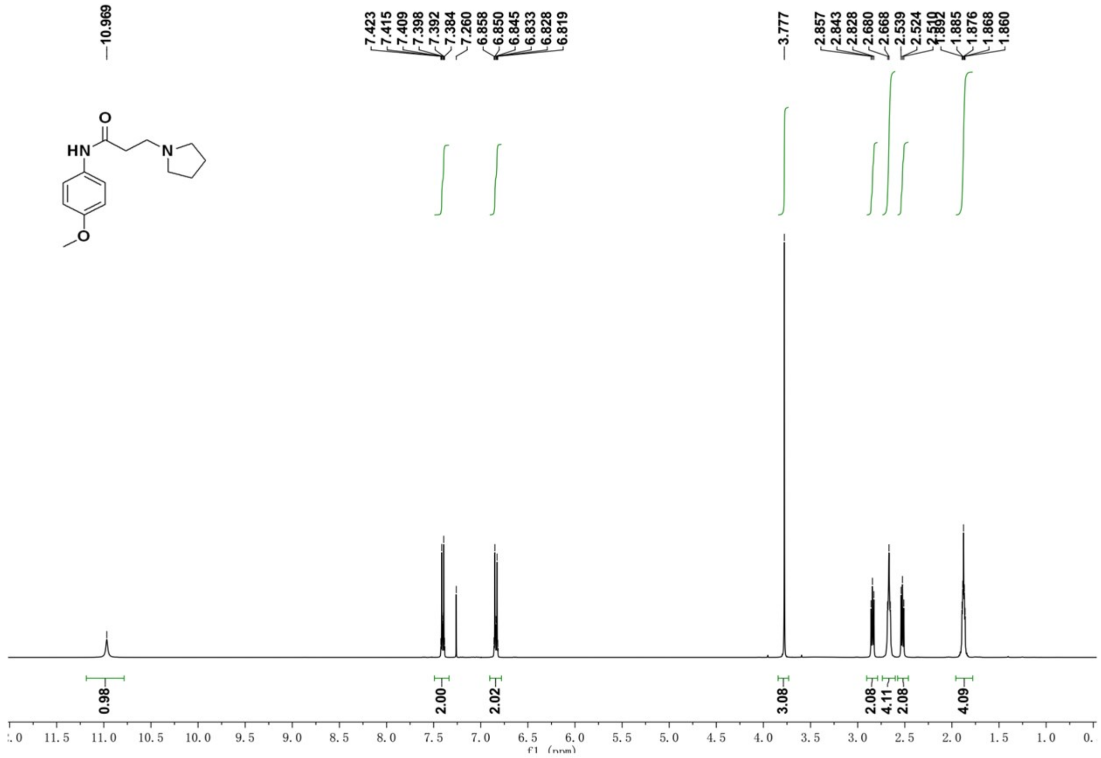

^{13}C NMR spectra of **3bb**

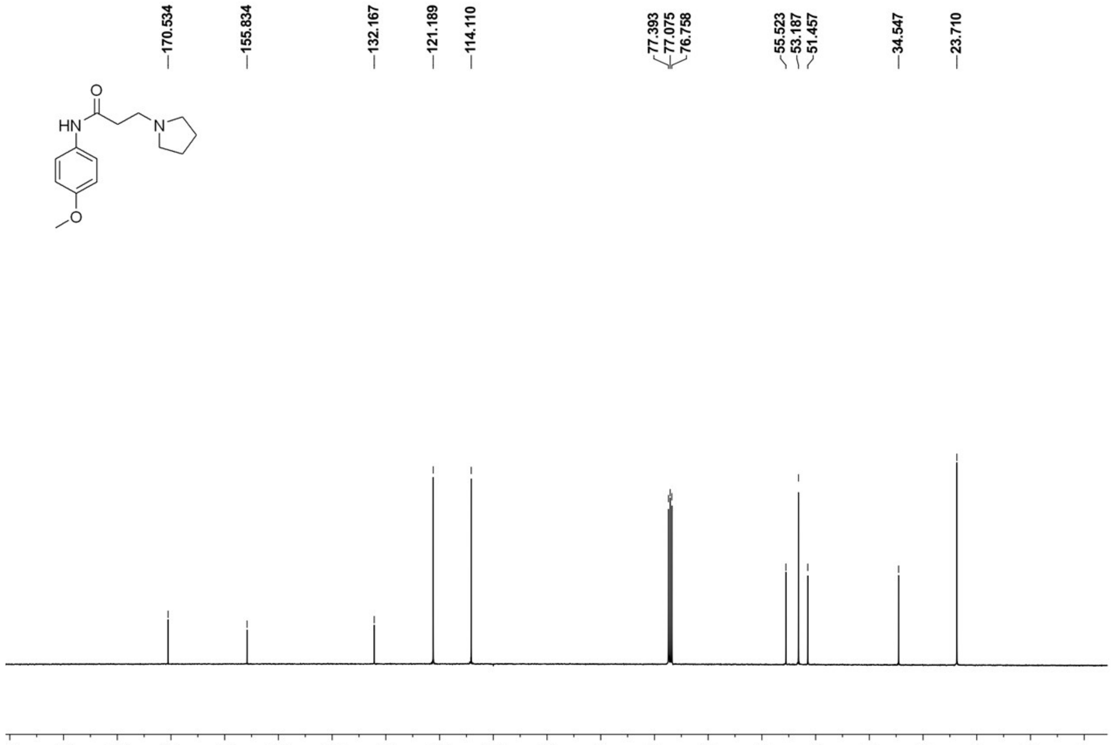

^{1}H NMR spectra of **3cb**

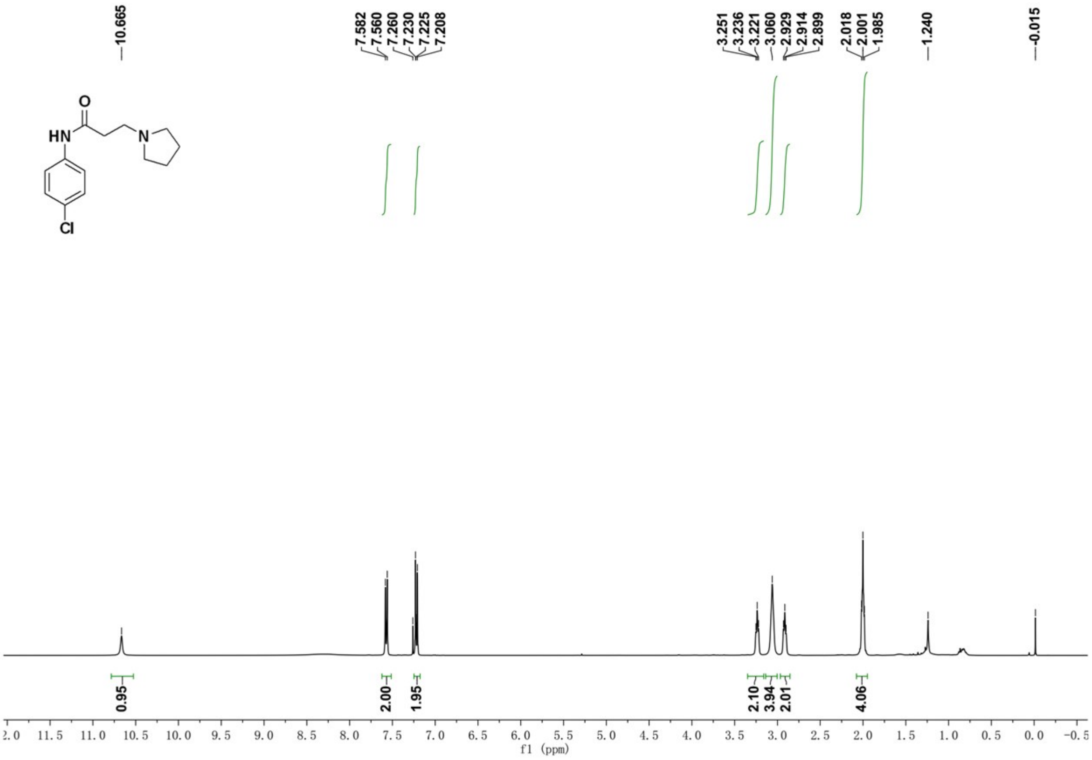

^{13}C NMR spectra of **3cb**

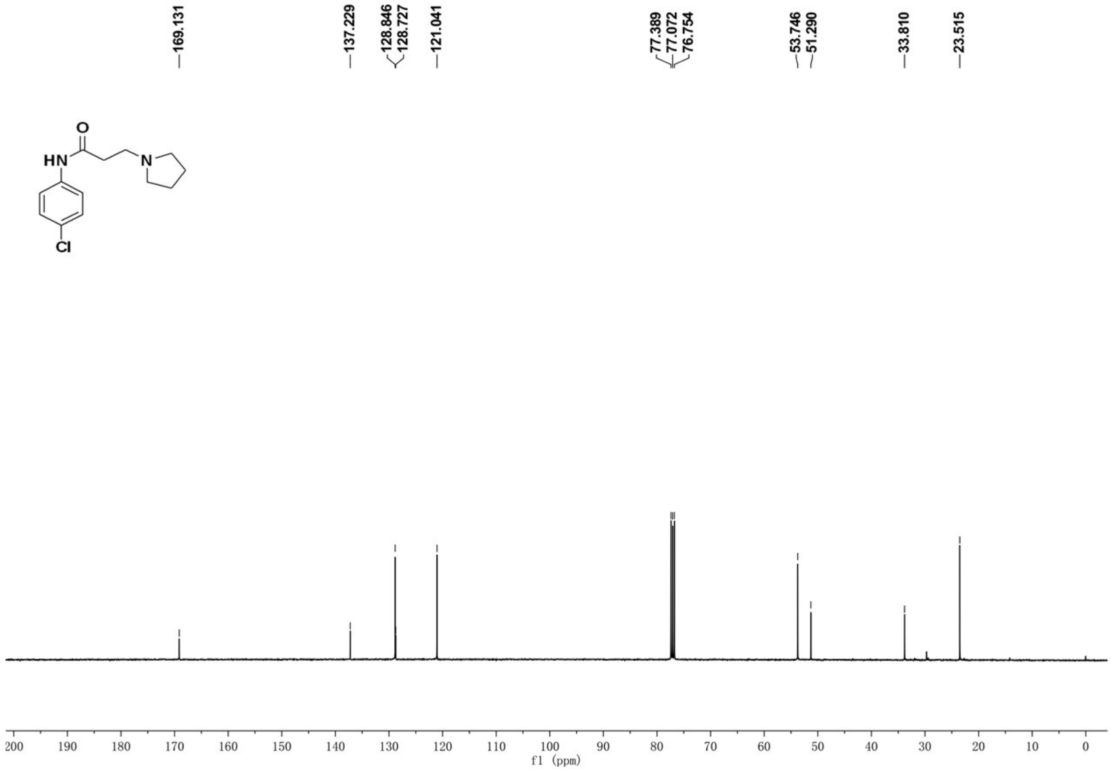

^{1}H NMR spectra of **3db**

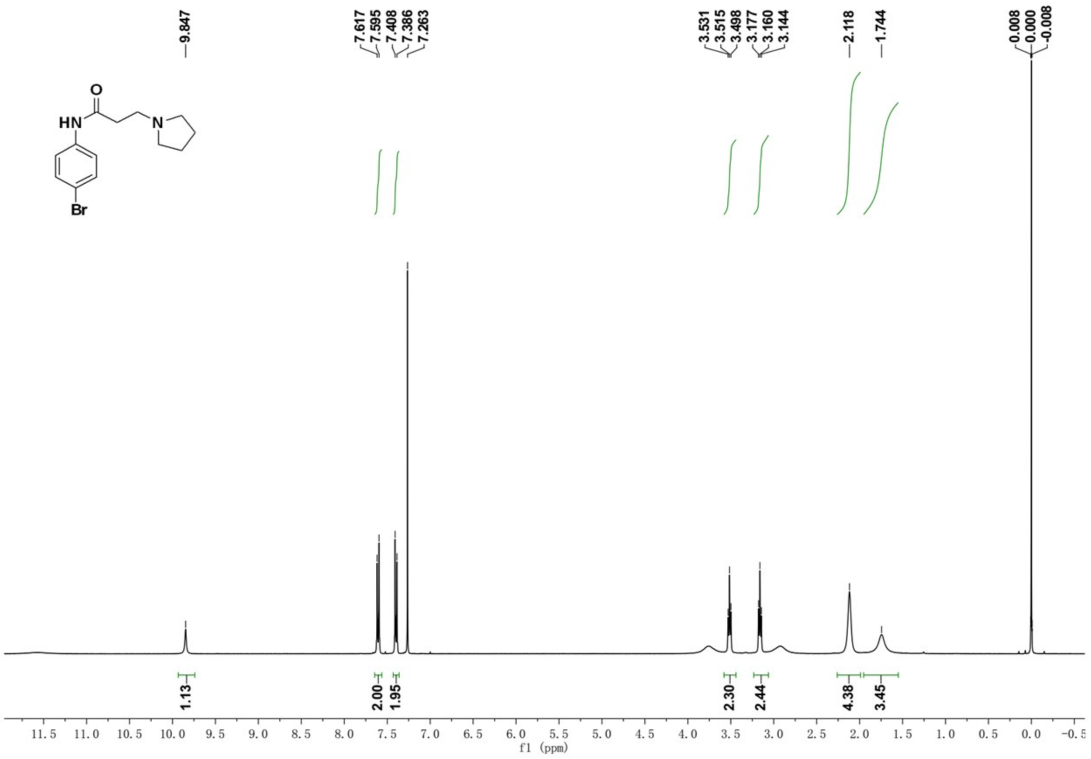

^{13}C NMR spectra of **3db**

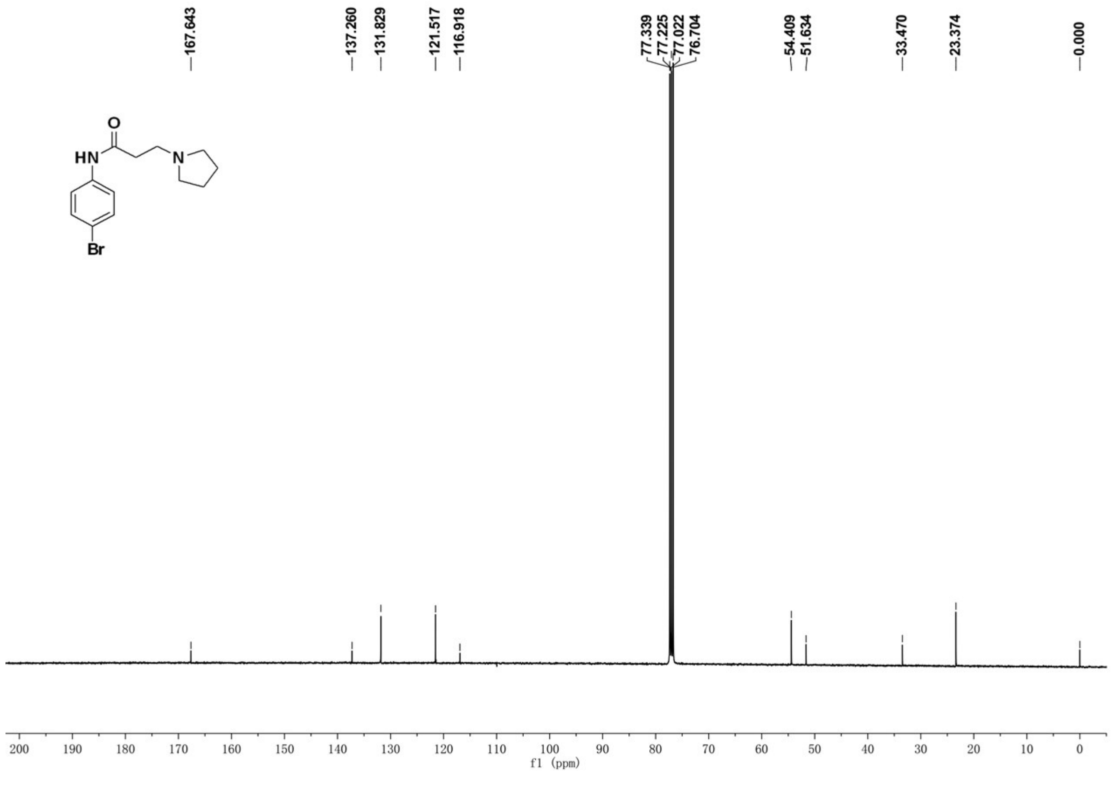

^{1}H NMR spectra of **3ac**

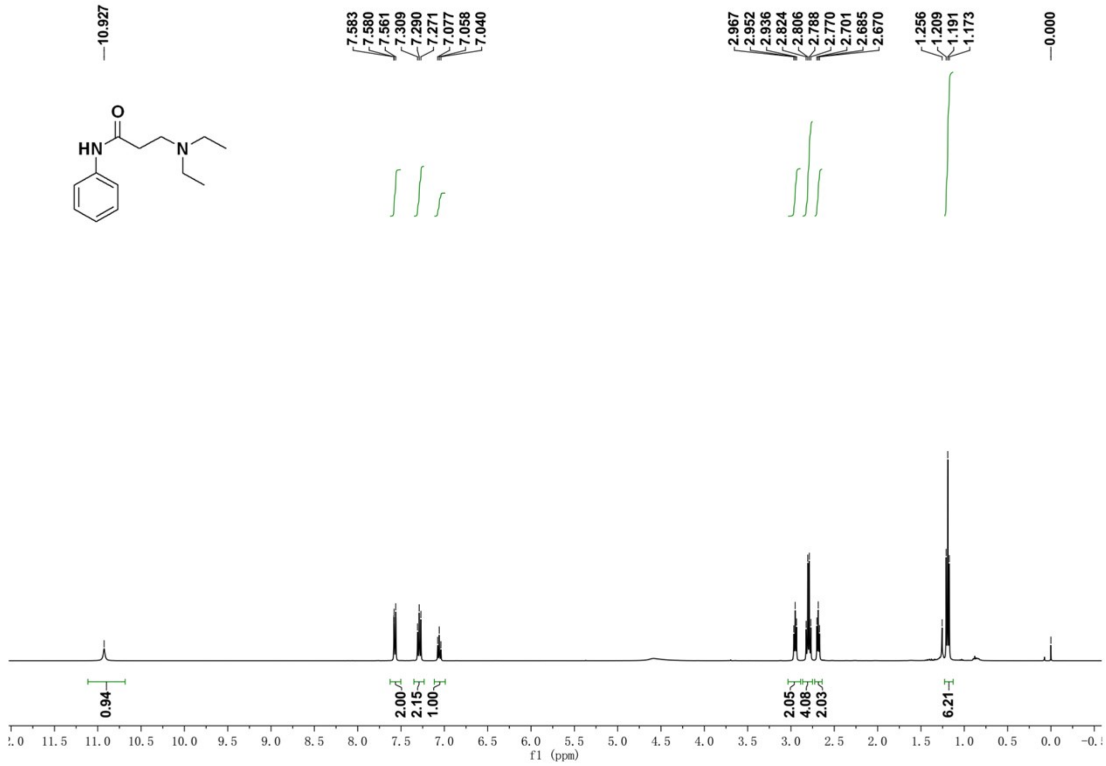

^{13}C NMR spectra of **3ac**

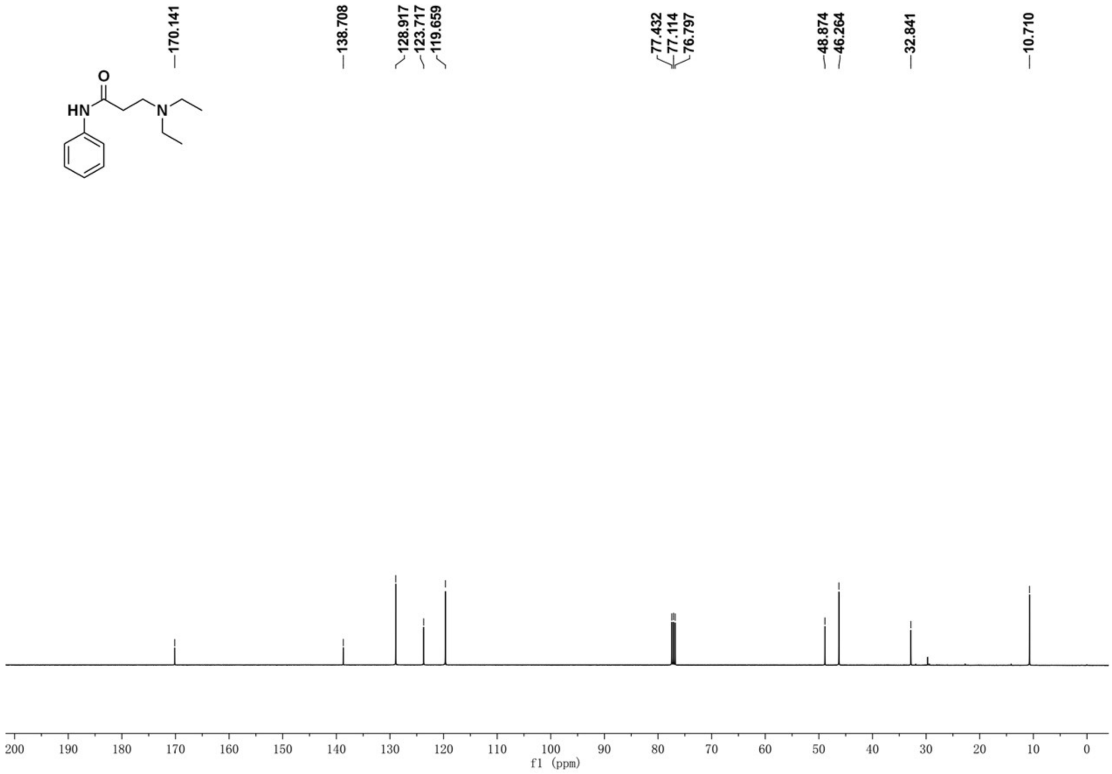

^{1}H NMR spectra of **3bc**

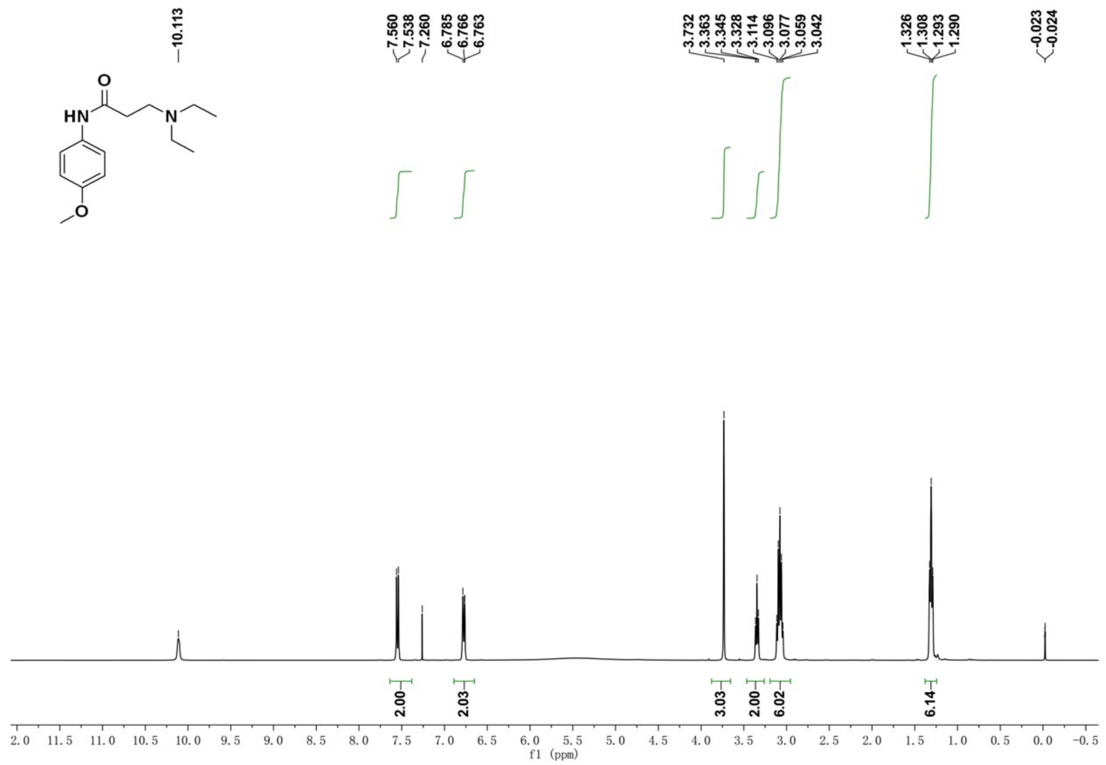

^{13}C NMR spectra of **3bc**

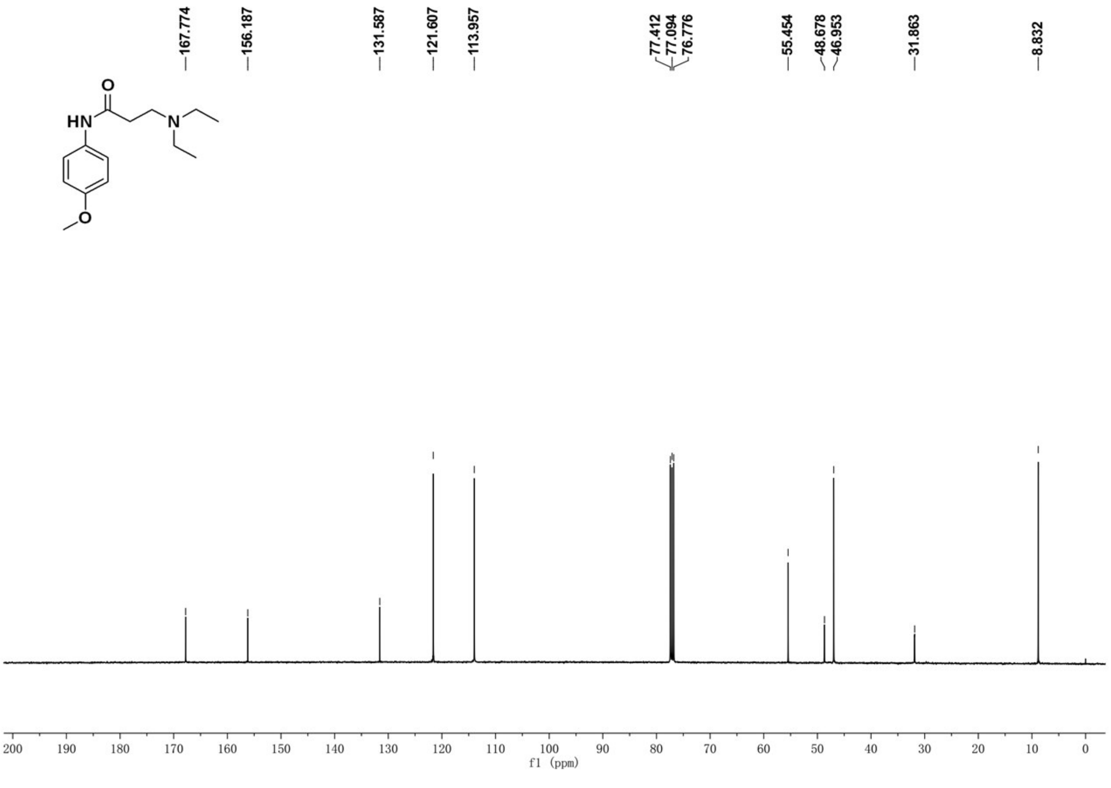

^{1}H NMR spectra of **3cc**

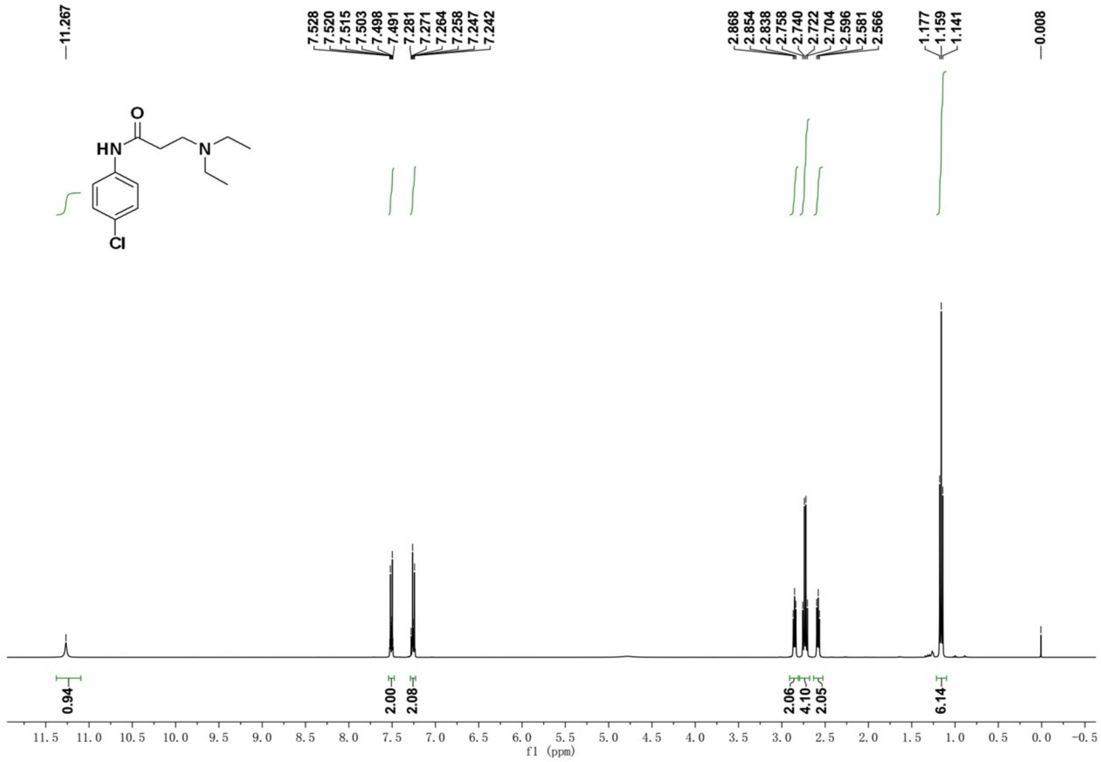

^{13}C NMR spectra of **3cc**

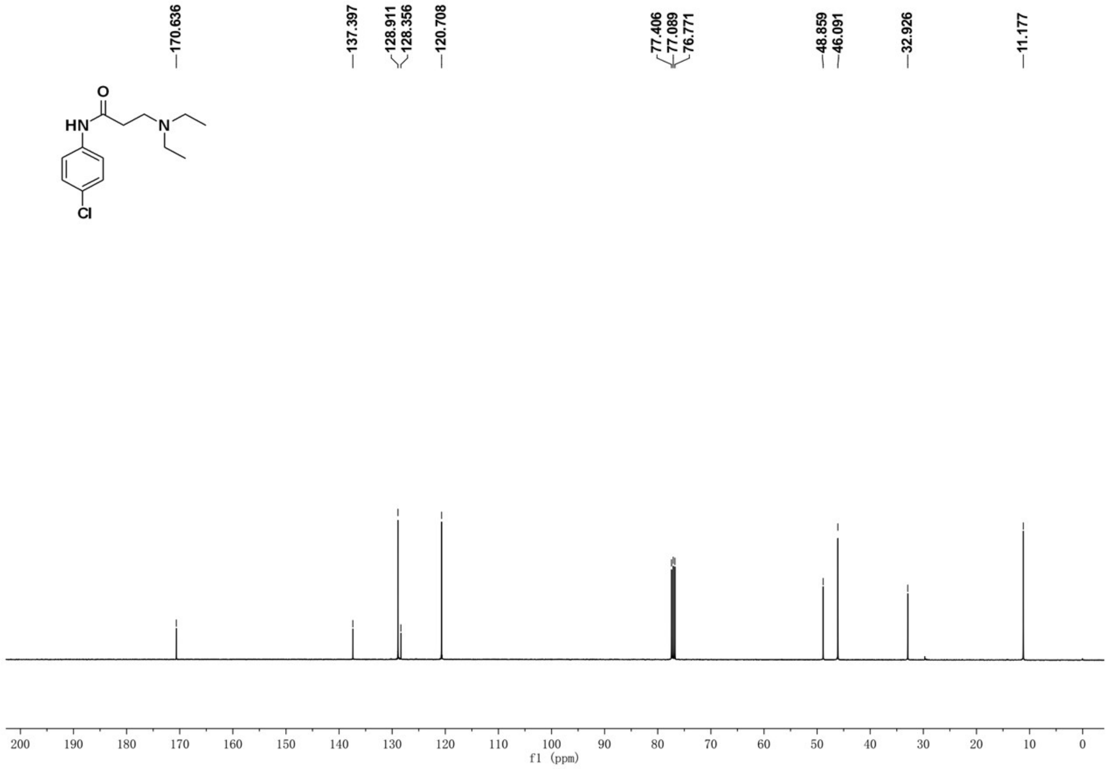

^{1}H NMR spectra of **3dc**

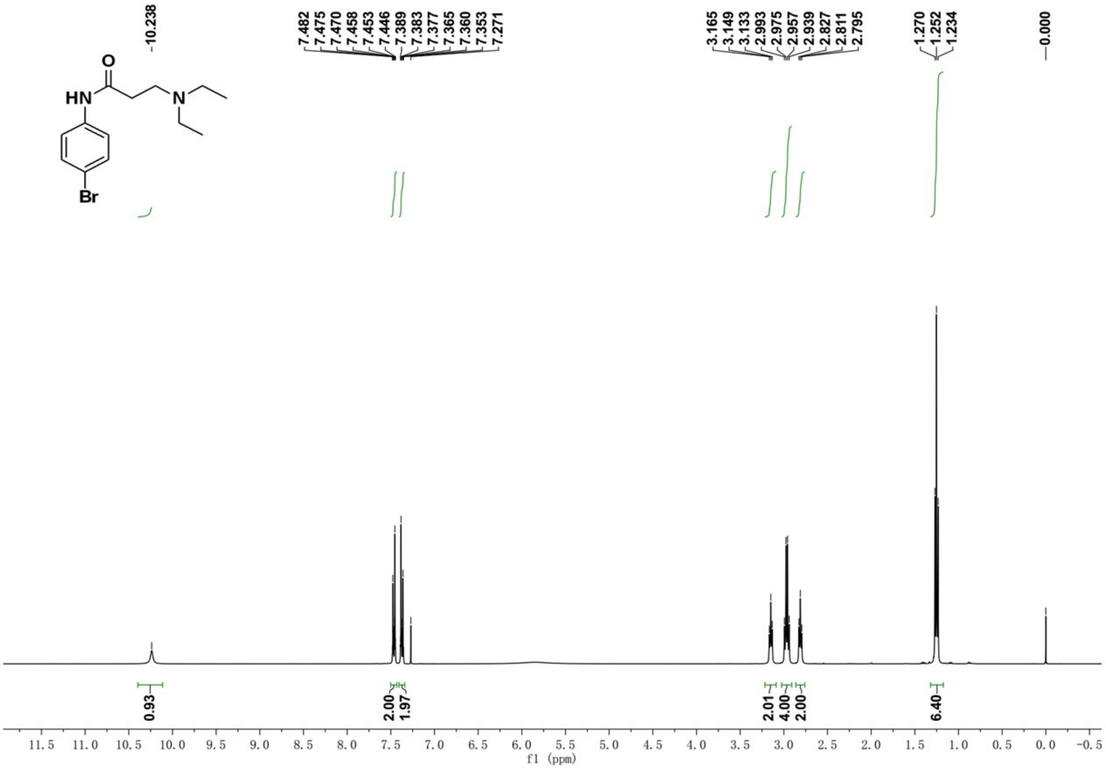

^{13}C NMR spectra of **3dc**

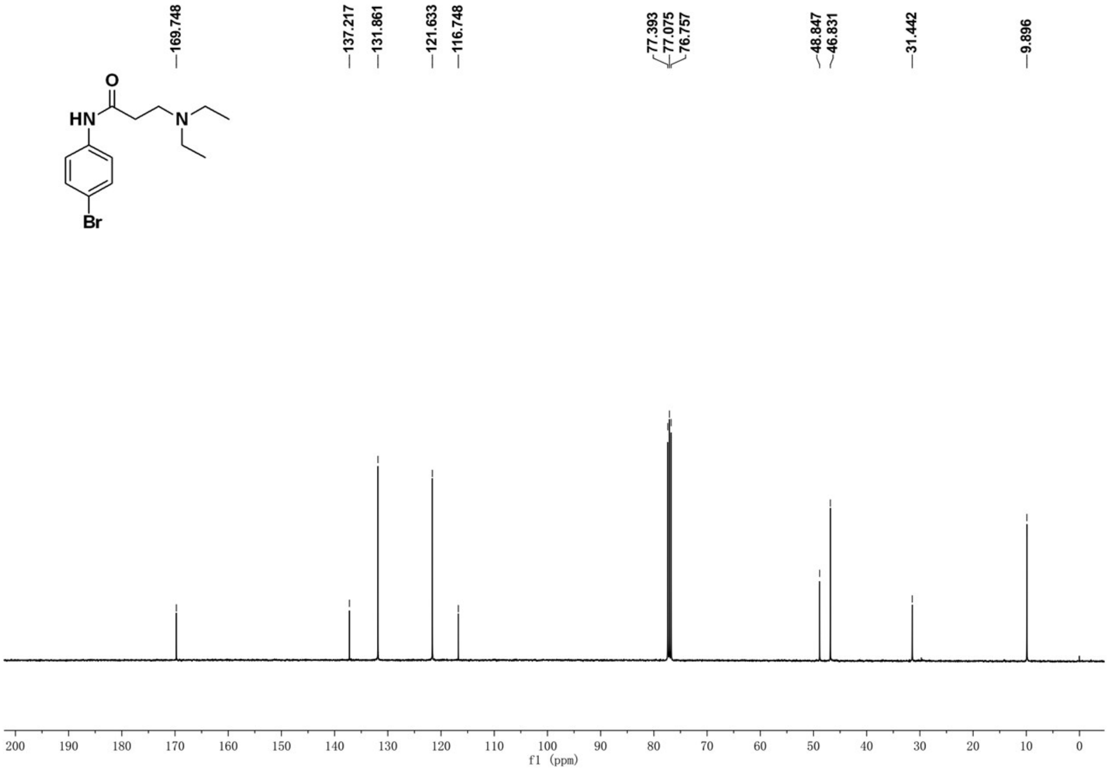

^{1}H NMR spectra of **3ad**

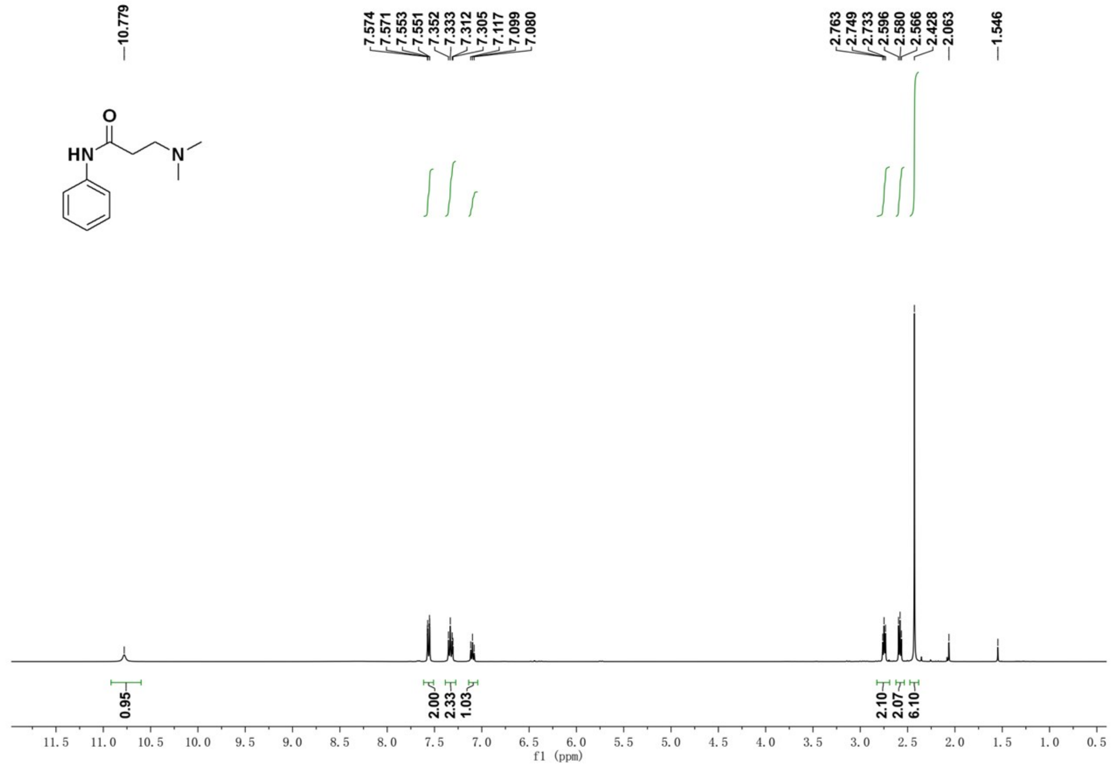

^{13}C NMR spectra of **3ad**

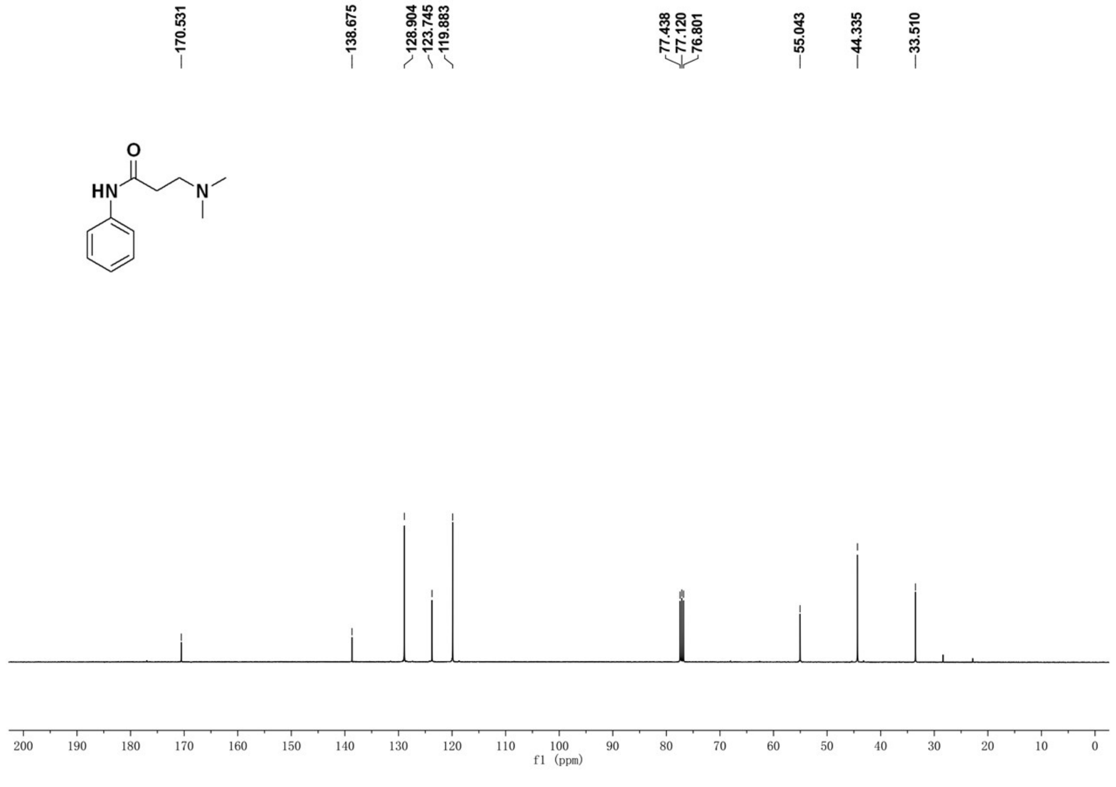

^{1}H NMR spectra of **3bd**

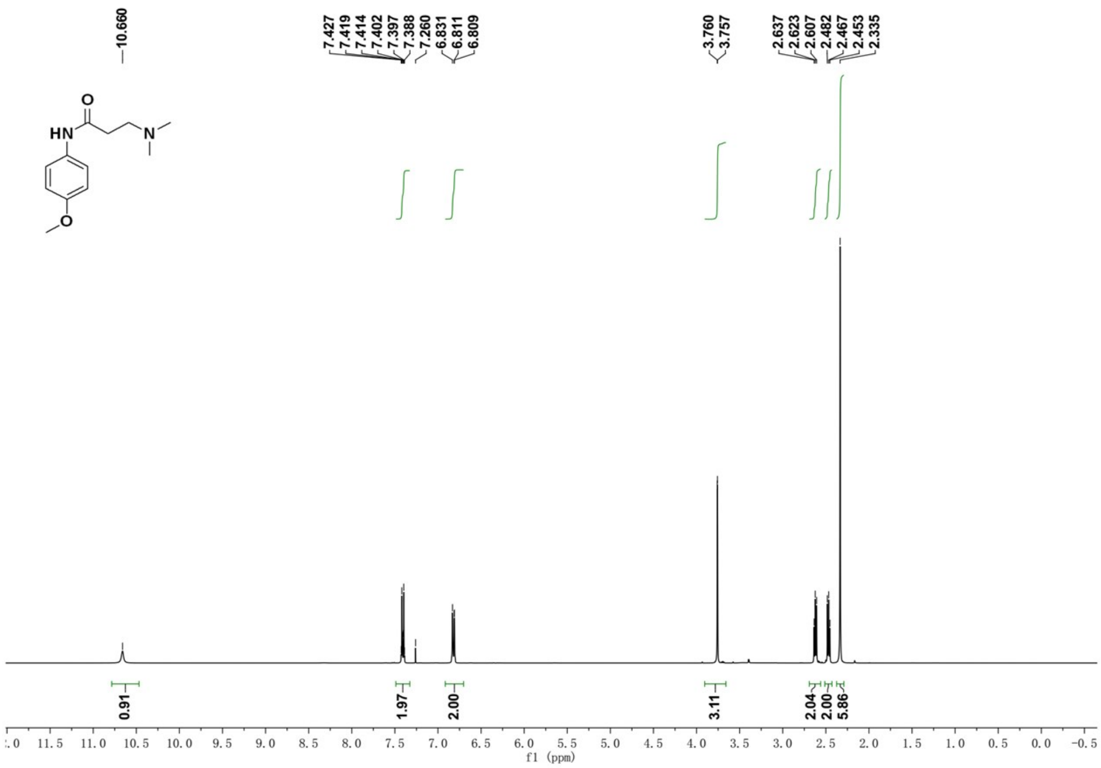

^{13}C NMR spectra of **3bd**

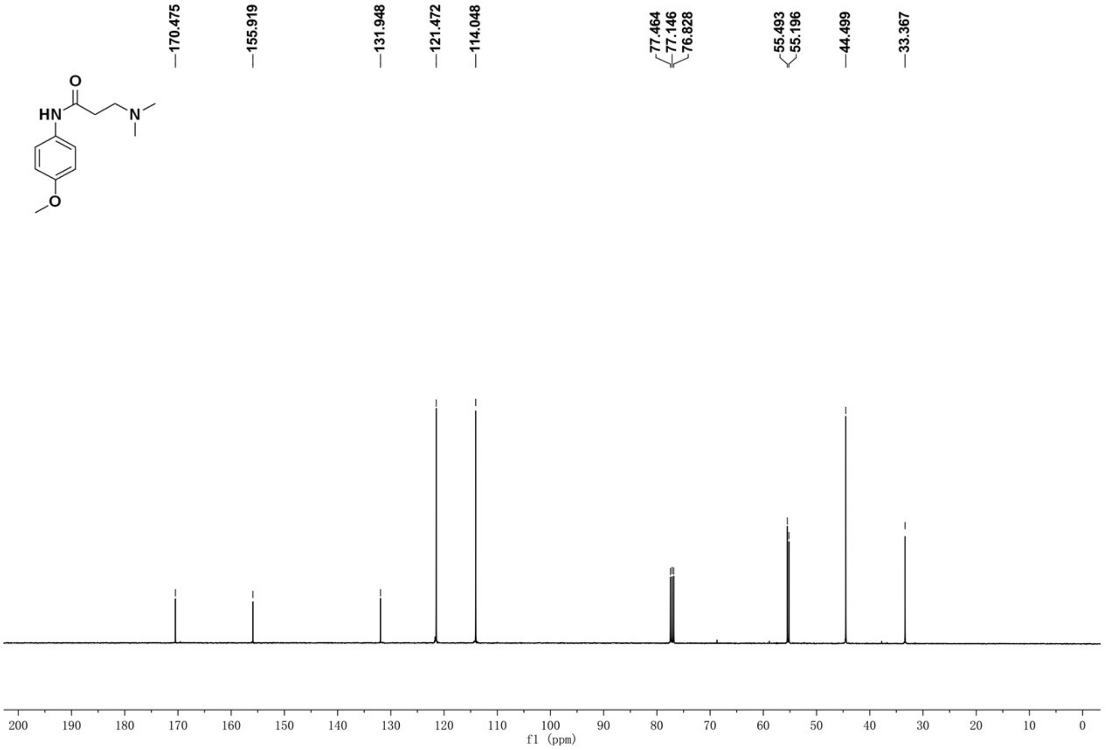

^{1}H NMR spectra of **3cd**

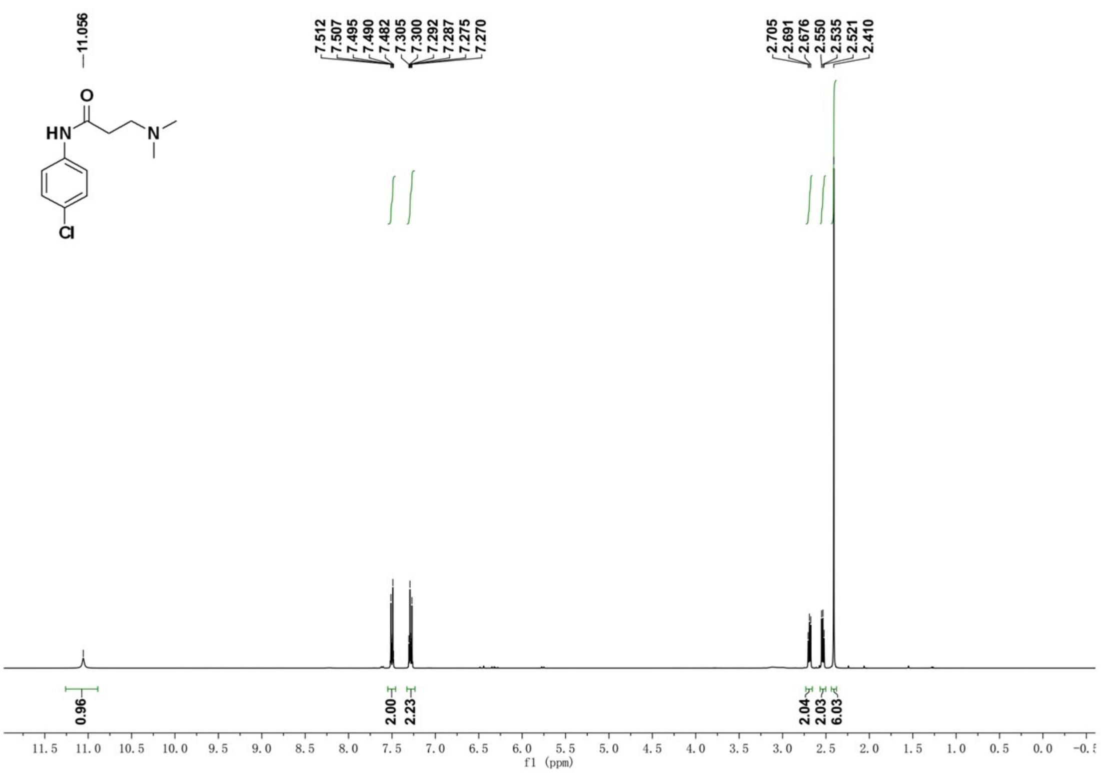

^{13}C NMR spectra of **3cd**

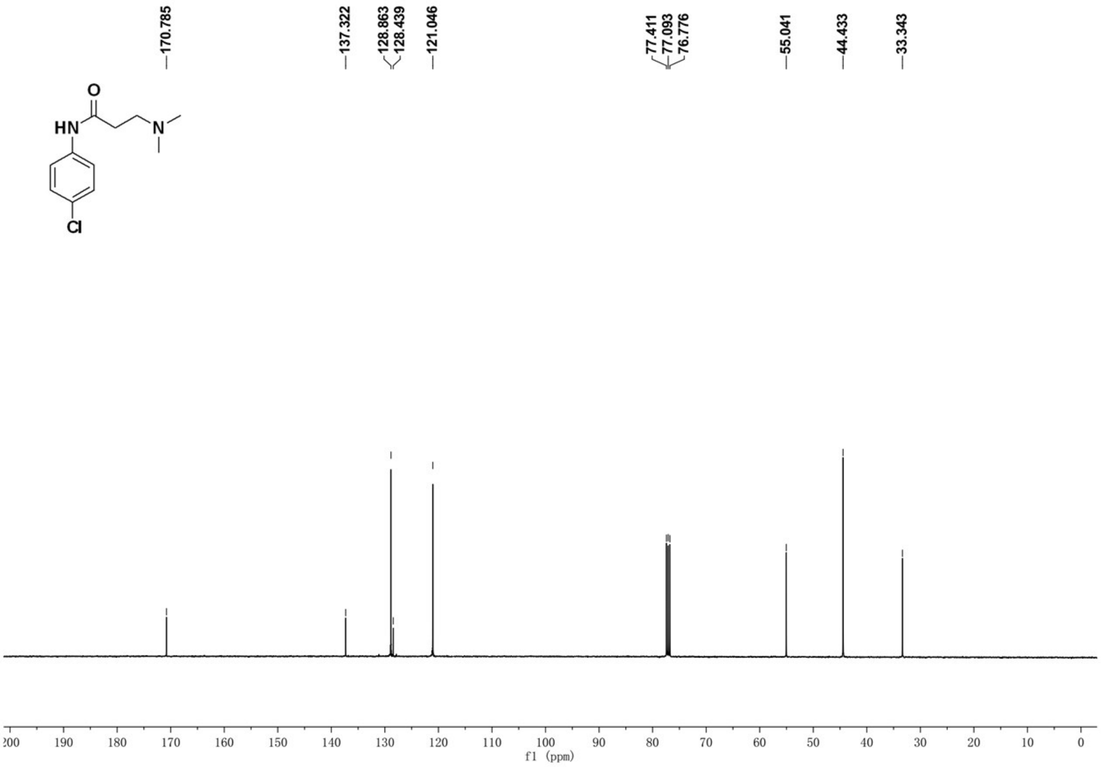

^{1}H NMR spectra of **3dd**

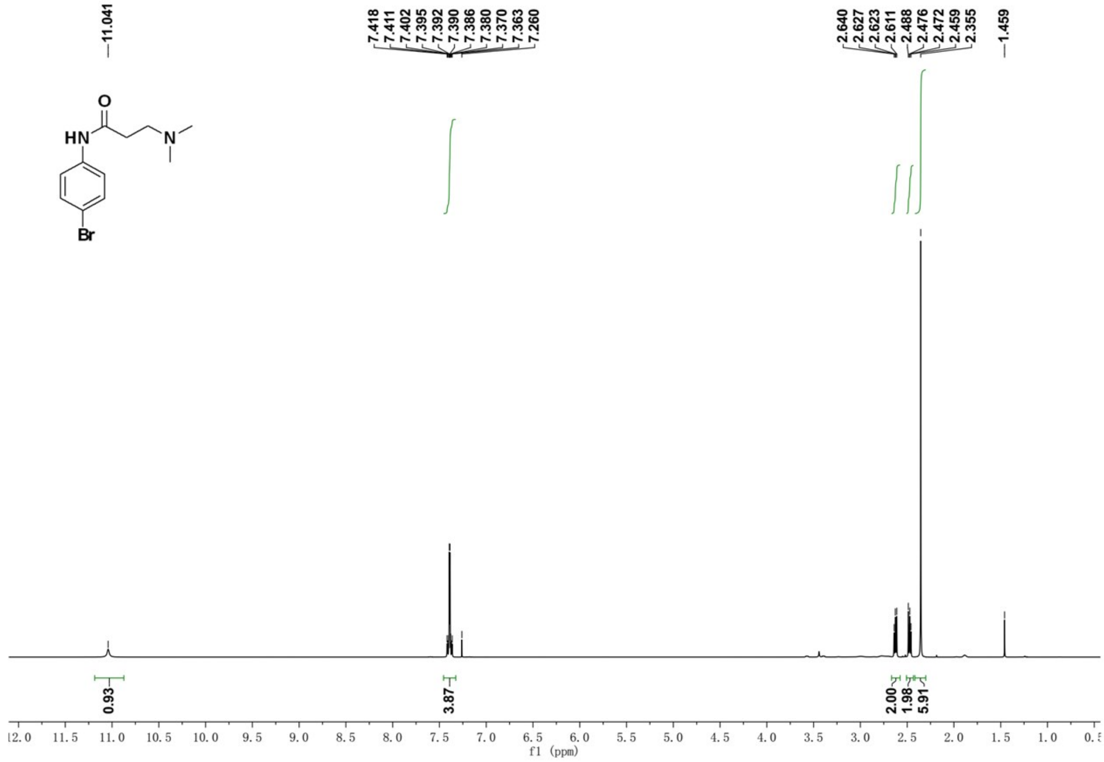

^{13}C NMR spectra of **3dd**

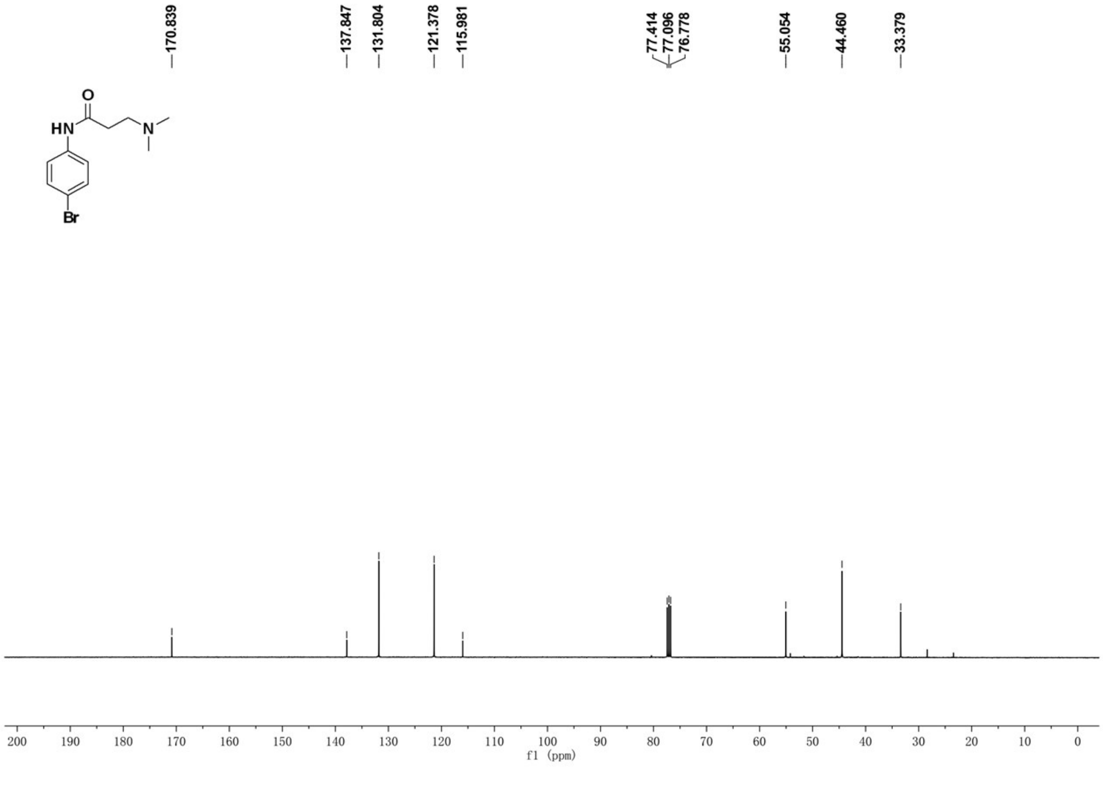

^{1}H NMR spectra of **3ae**

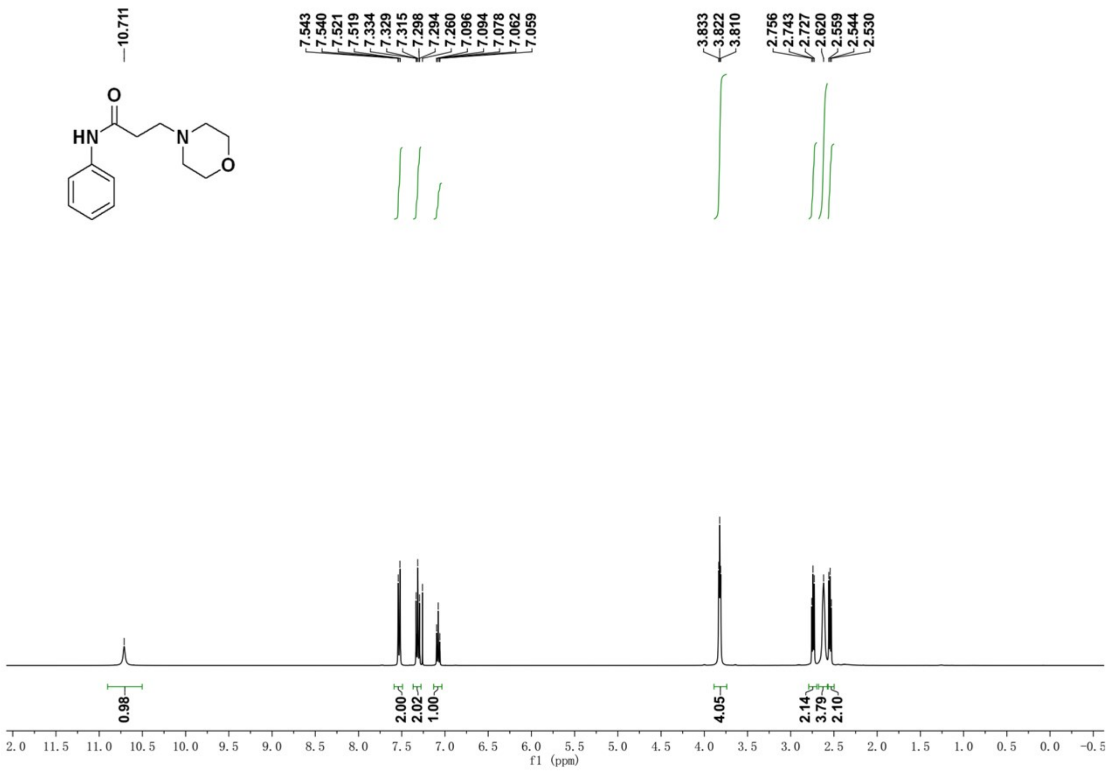

^{13}C NMR spectra of **3ae**

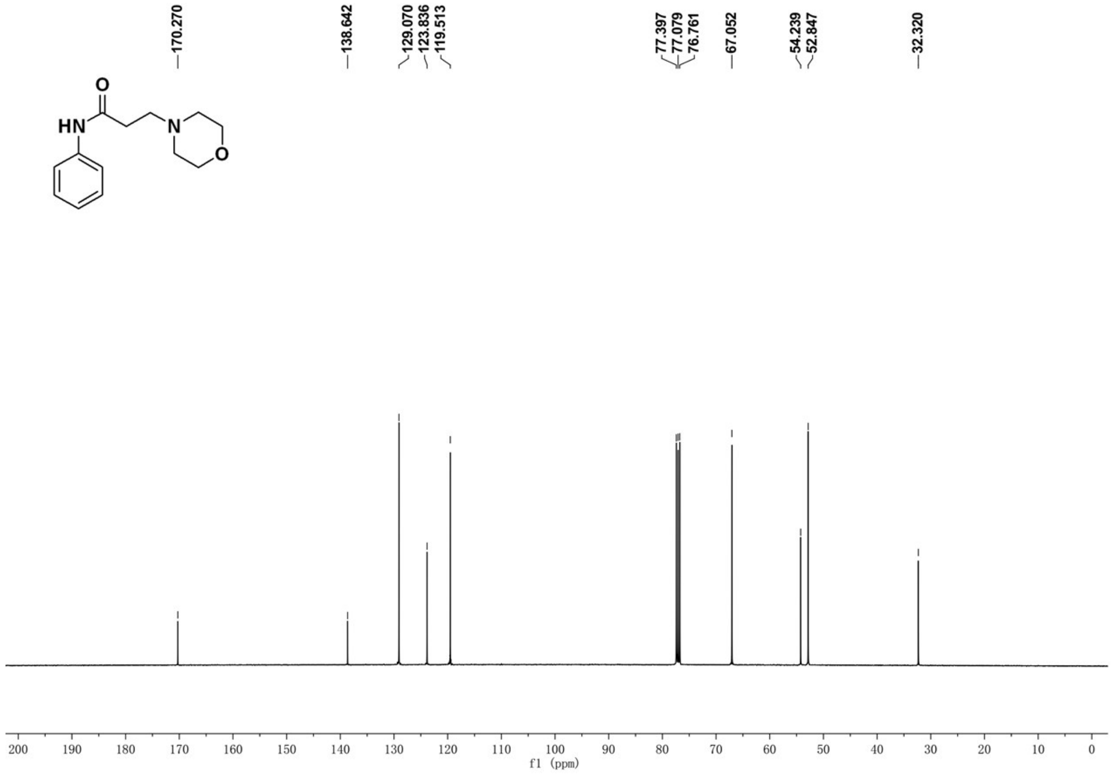

^1H NMR spectra of **3be**

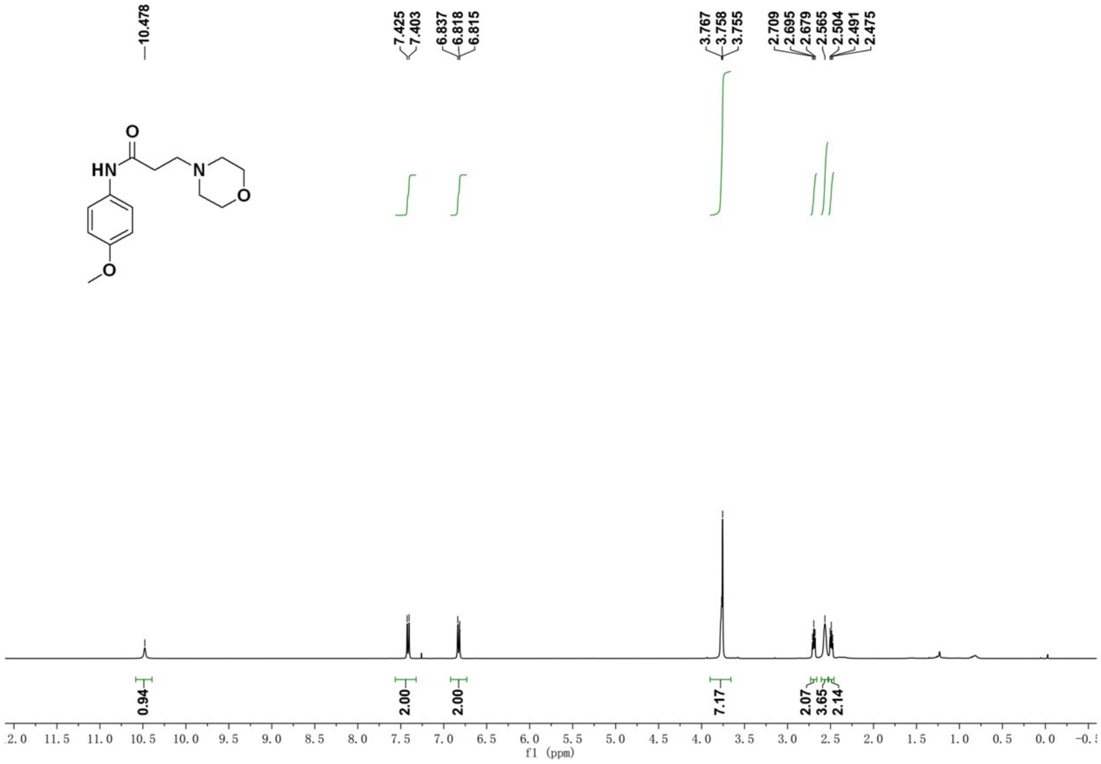

^{13}C NMR spectra of **3be**

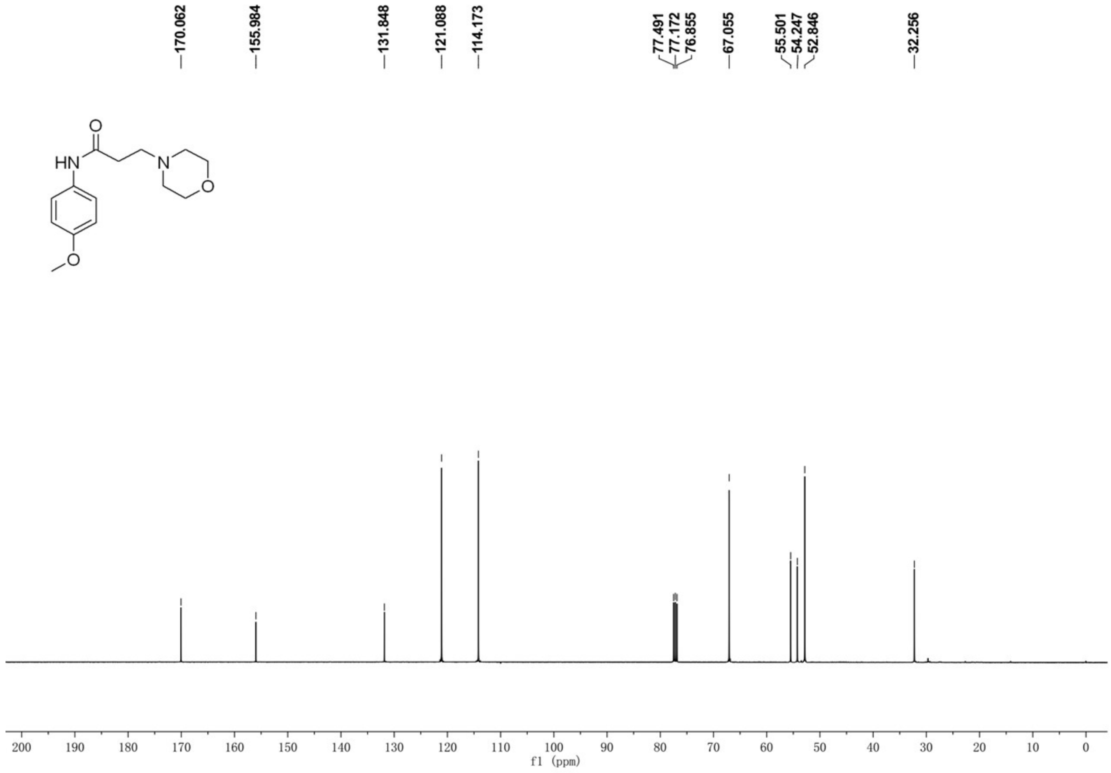

^{1}H NMR spectra of **3ce**

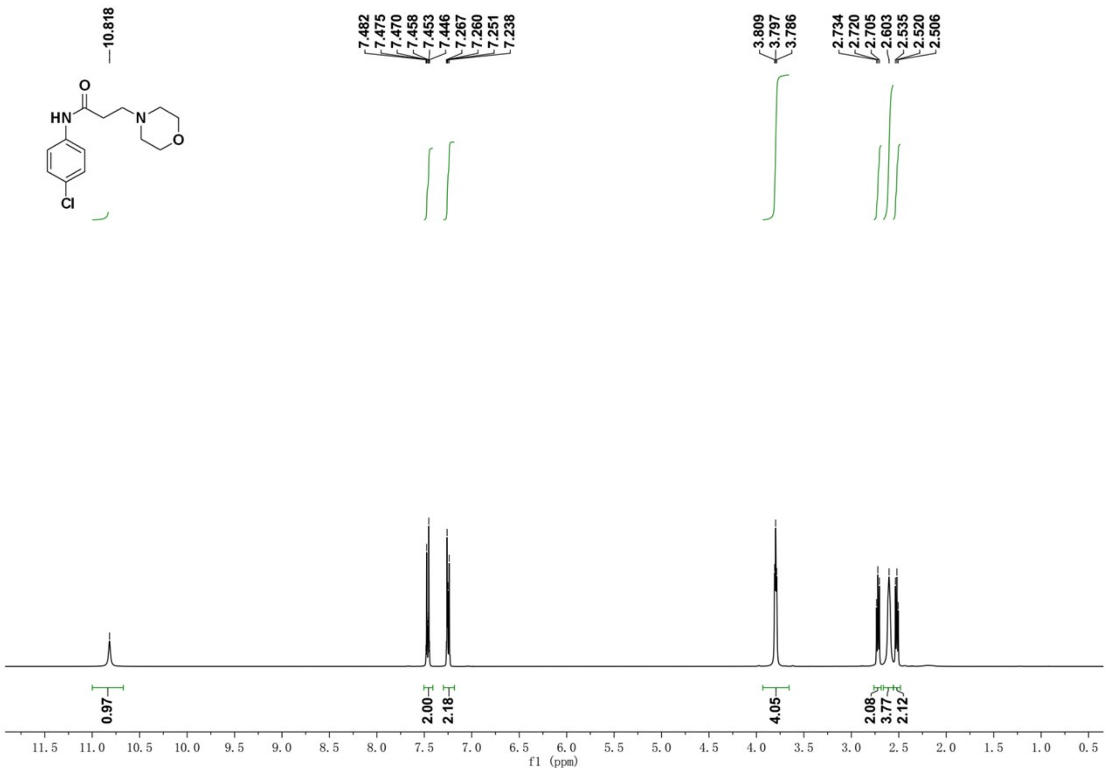

^{13}C NMR spectra of **3ce**

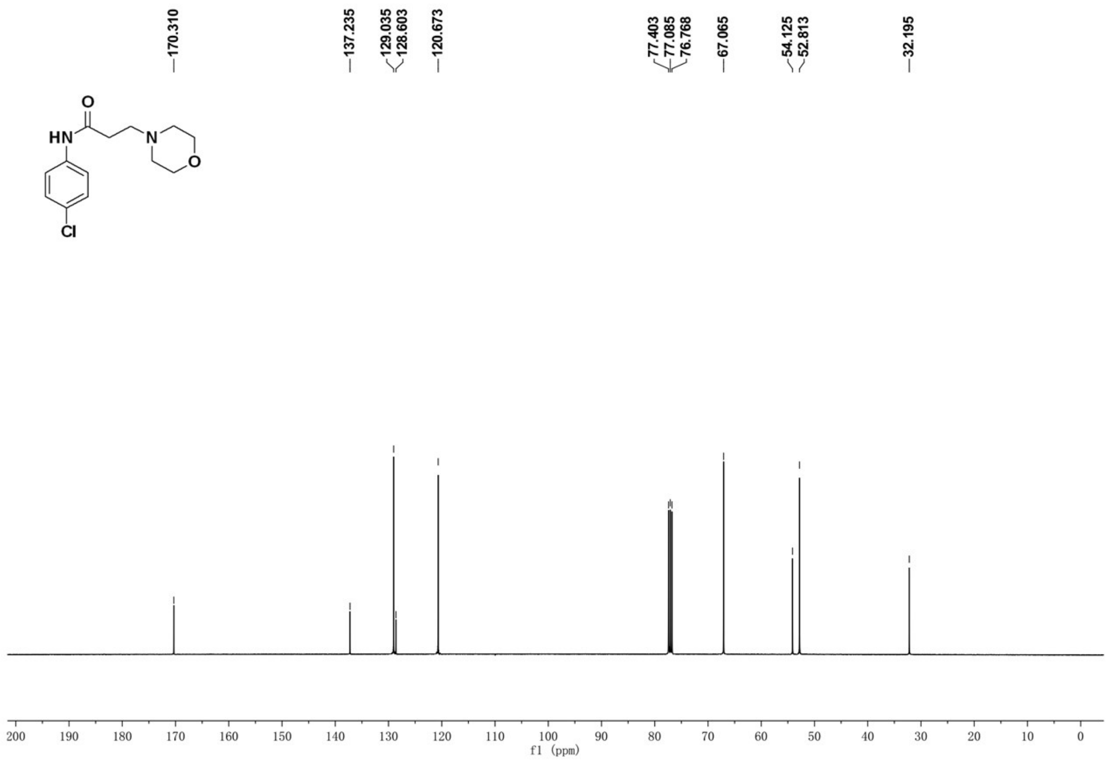

^{1}H NMR spectra of **3de**

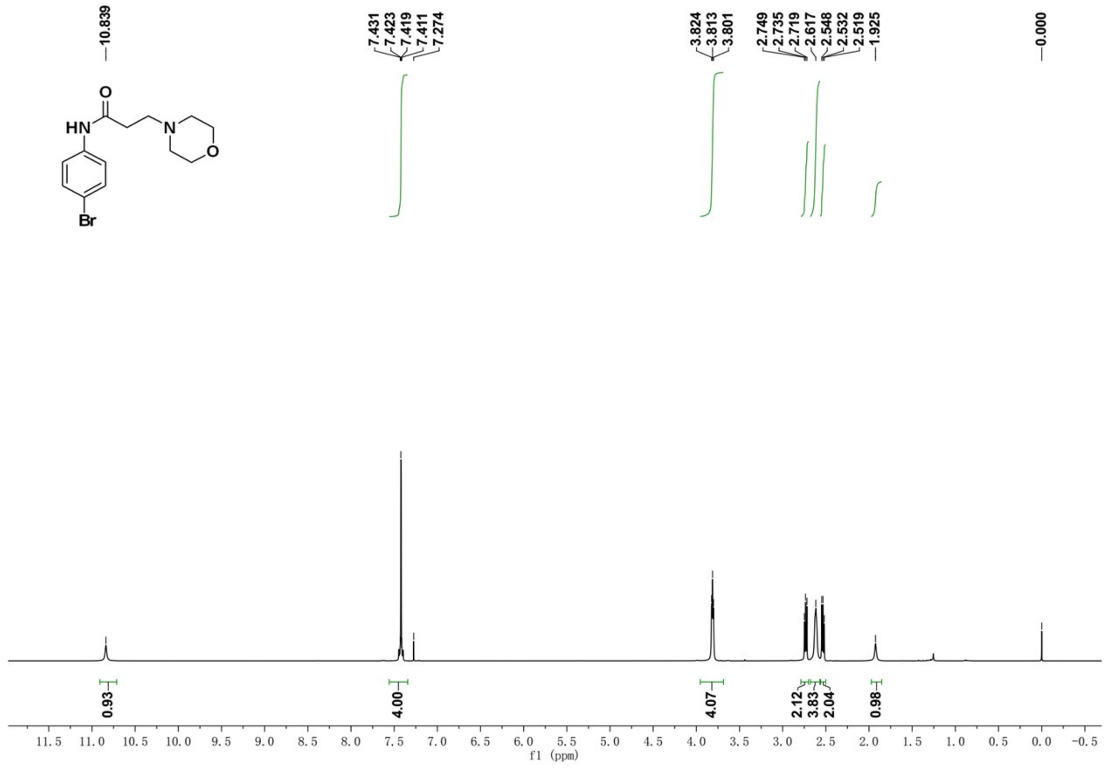

^{13}C NMR spectra of **3de**

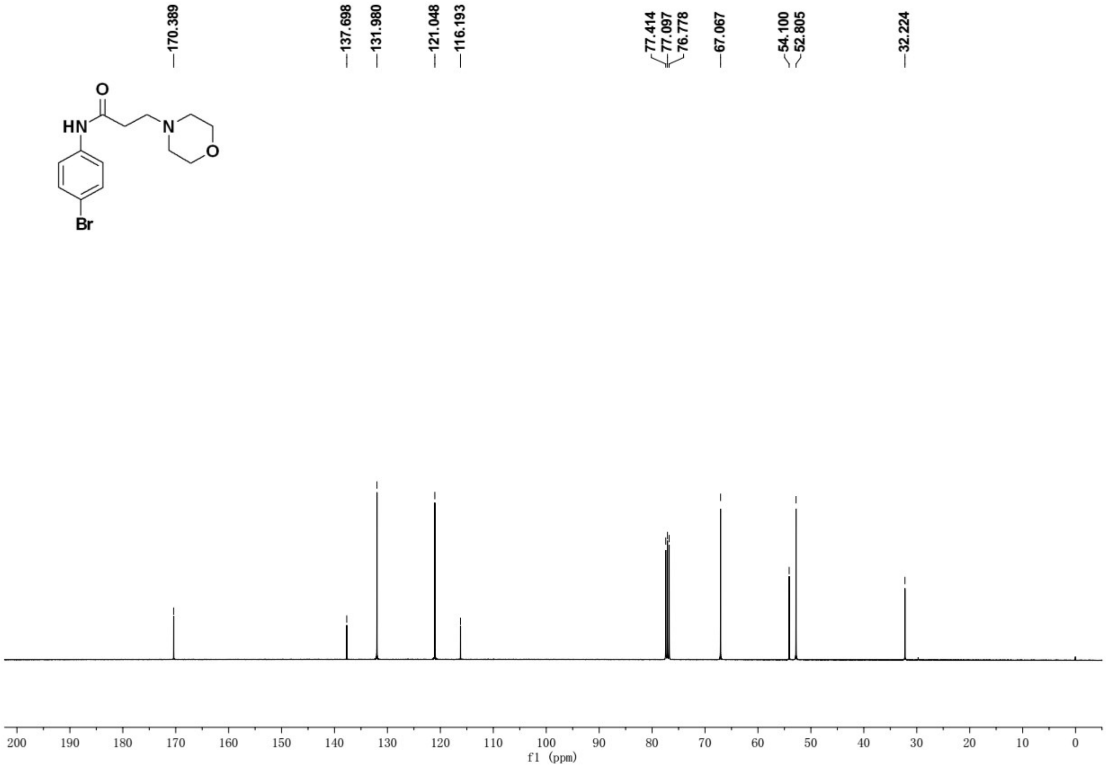

^{1}H NMR spectra of **3af**

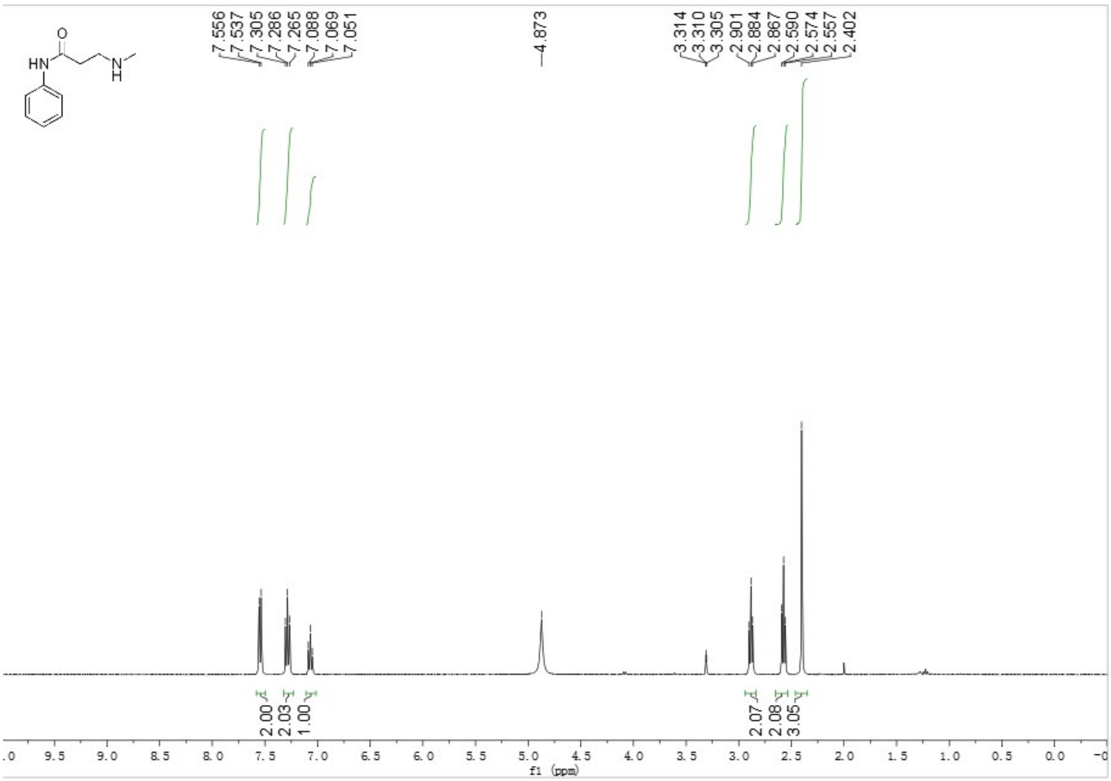

^{13}C NMR spectra of **3af**

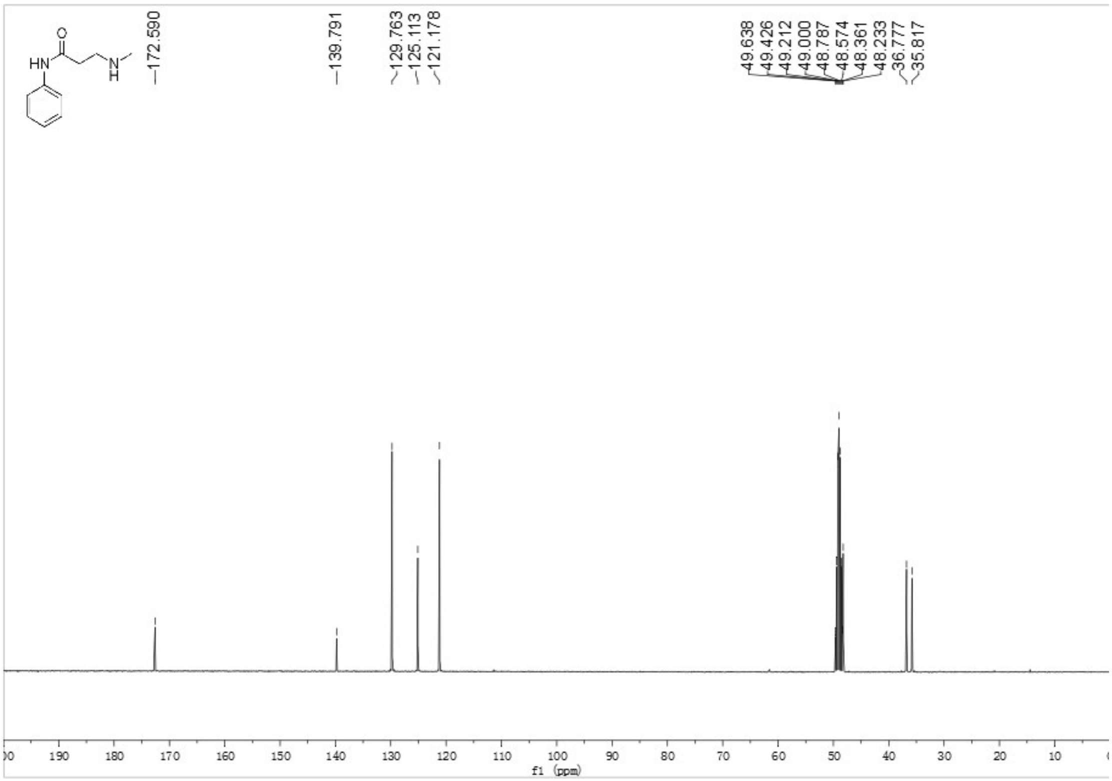

^{1}H NMR spectra of **3ed**

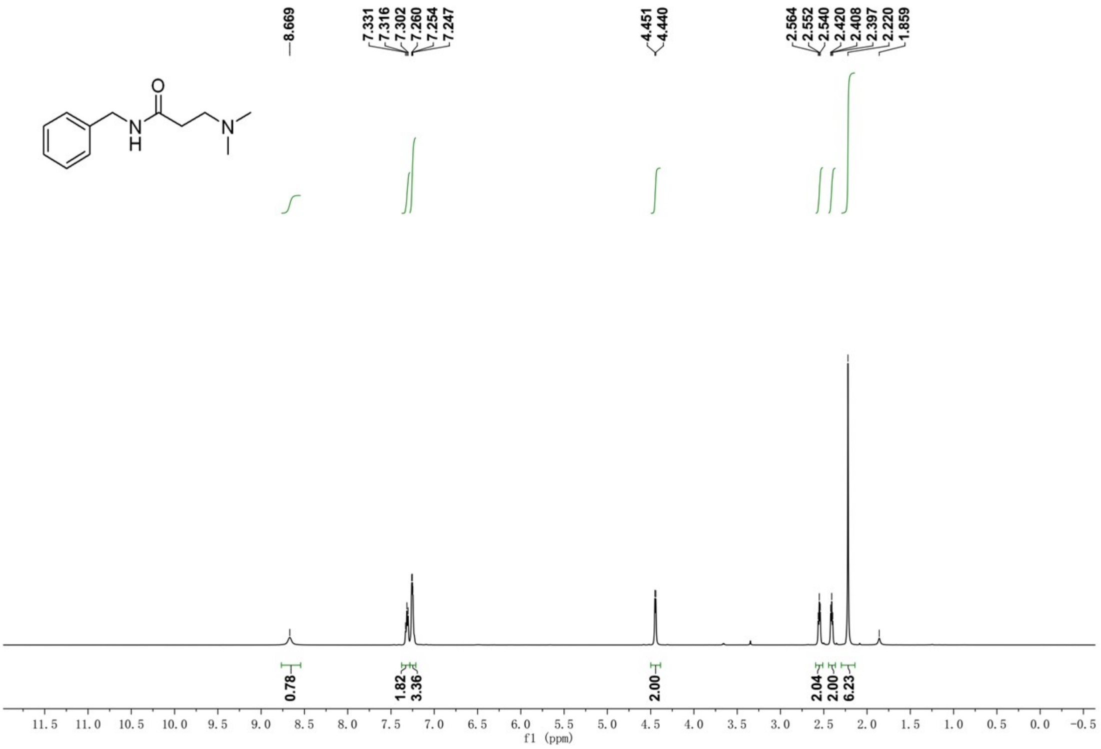

^{13}C NMR spectra of **3ed**

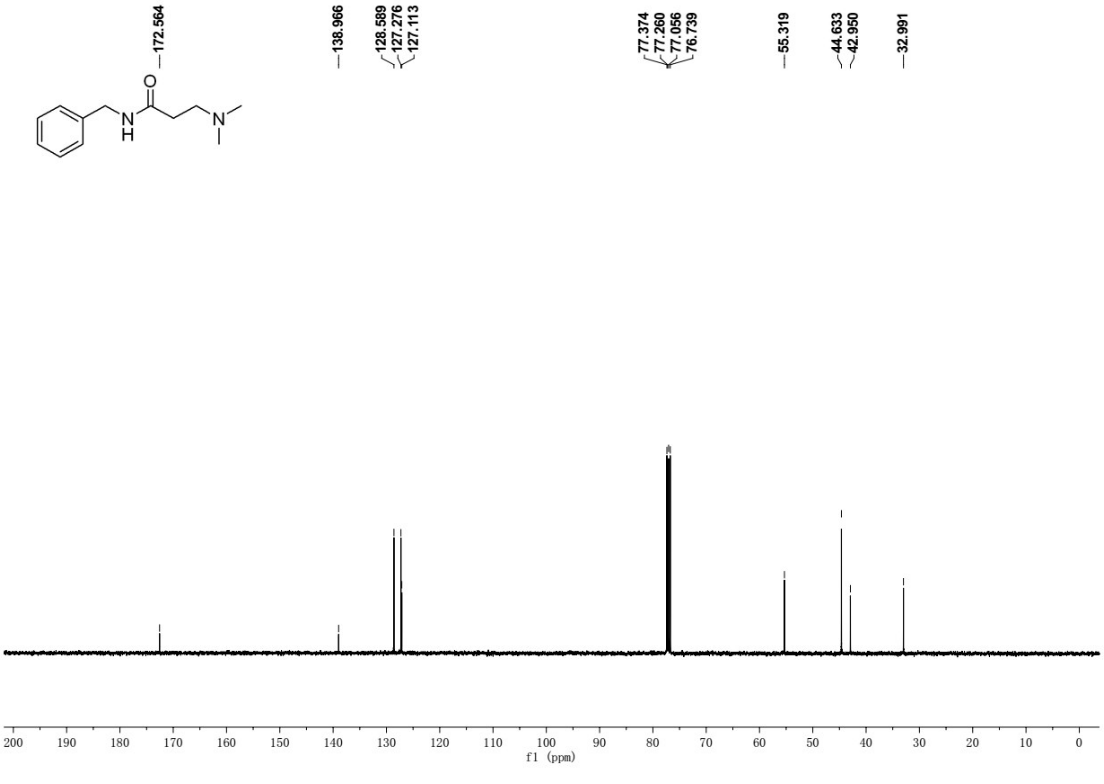

^{1}H NMR spectra of **3fa**

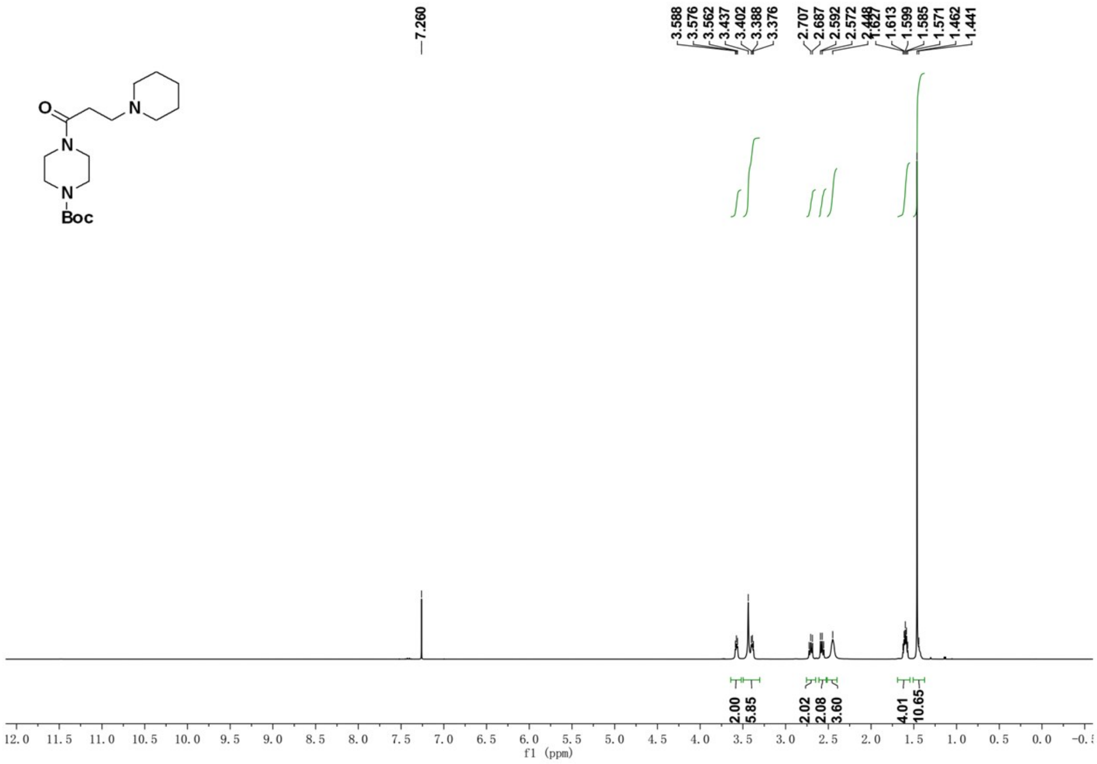

^{13}C NMR spectra of **3fa**

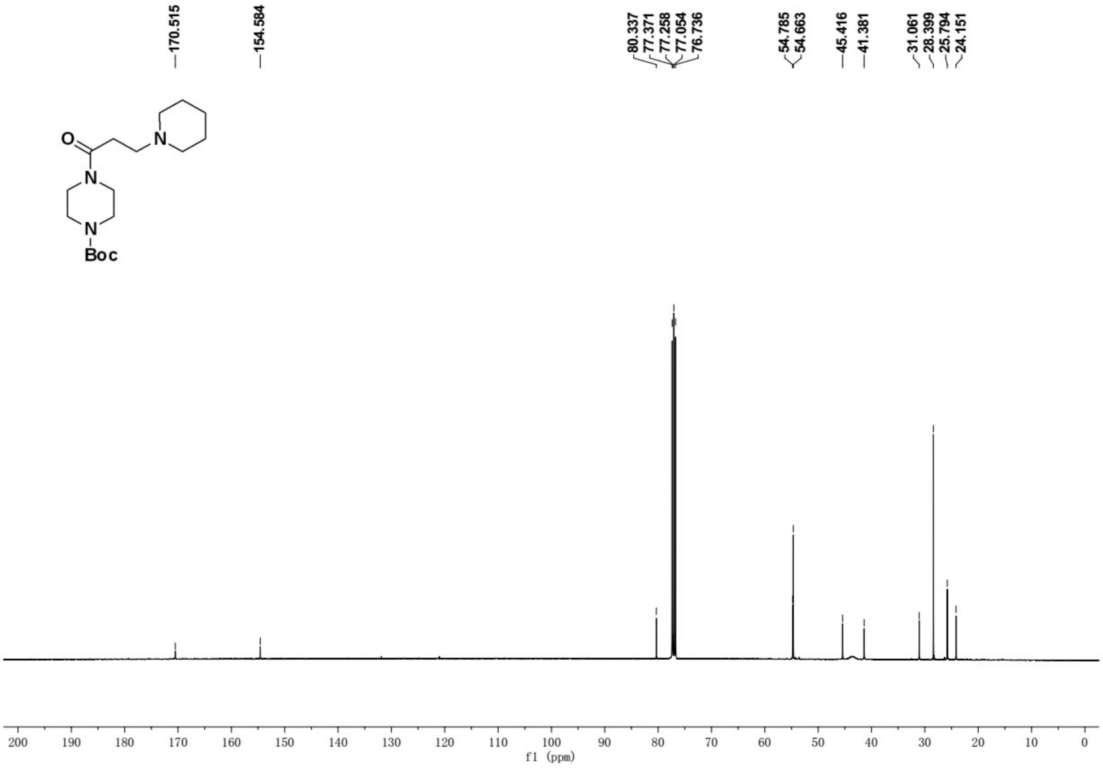

^{1}H NMR spectra of **3fe**

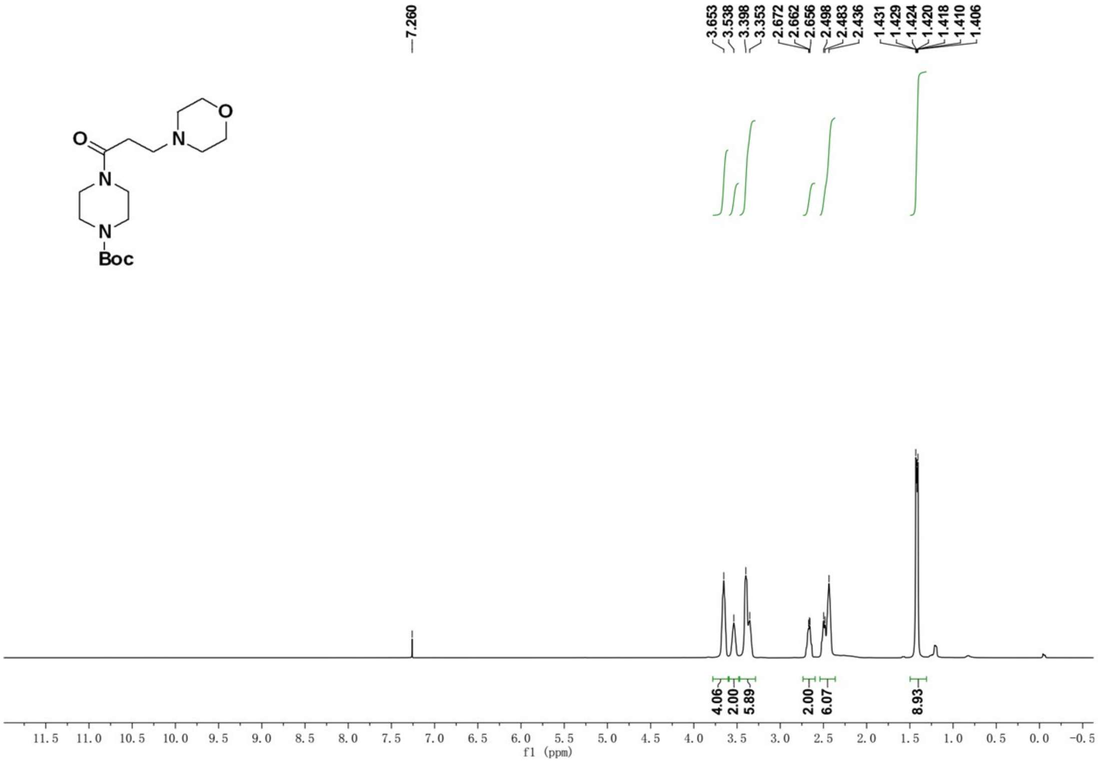

^{13}C NMR spectra of **3fe**

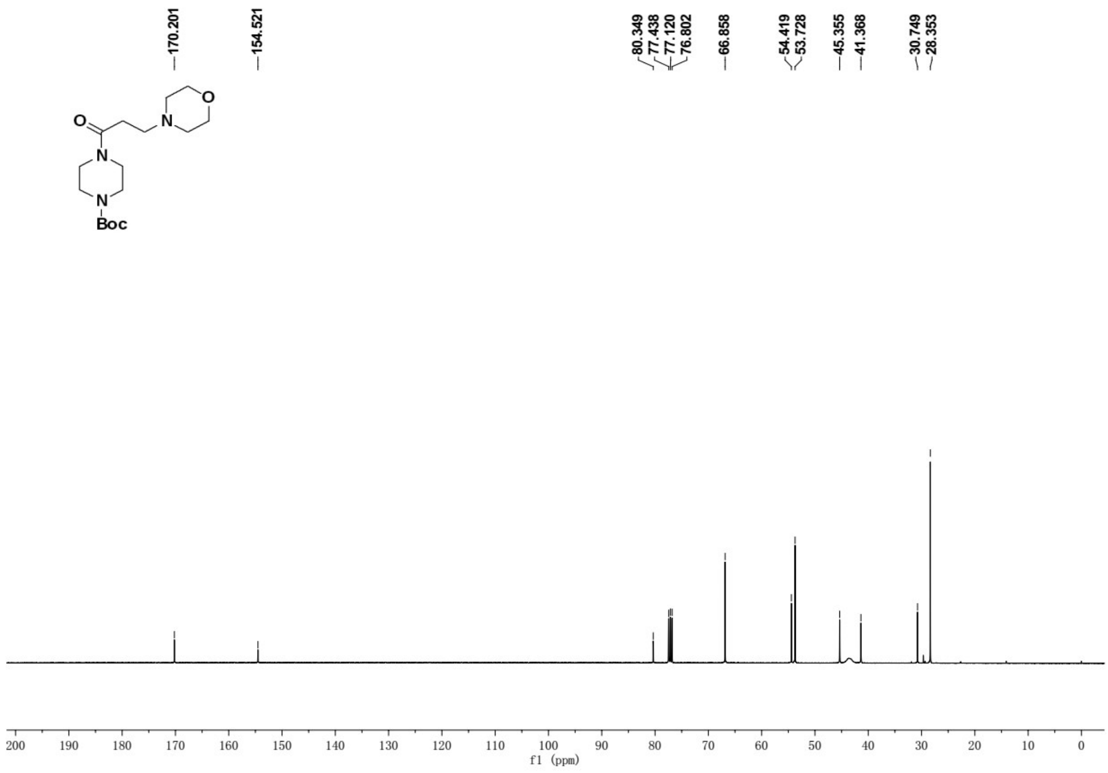

^{1}H NMR spectra of **3fb**

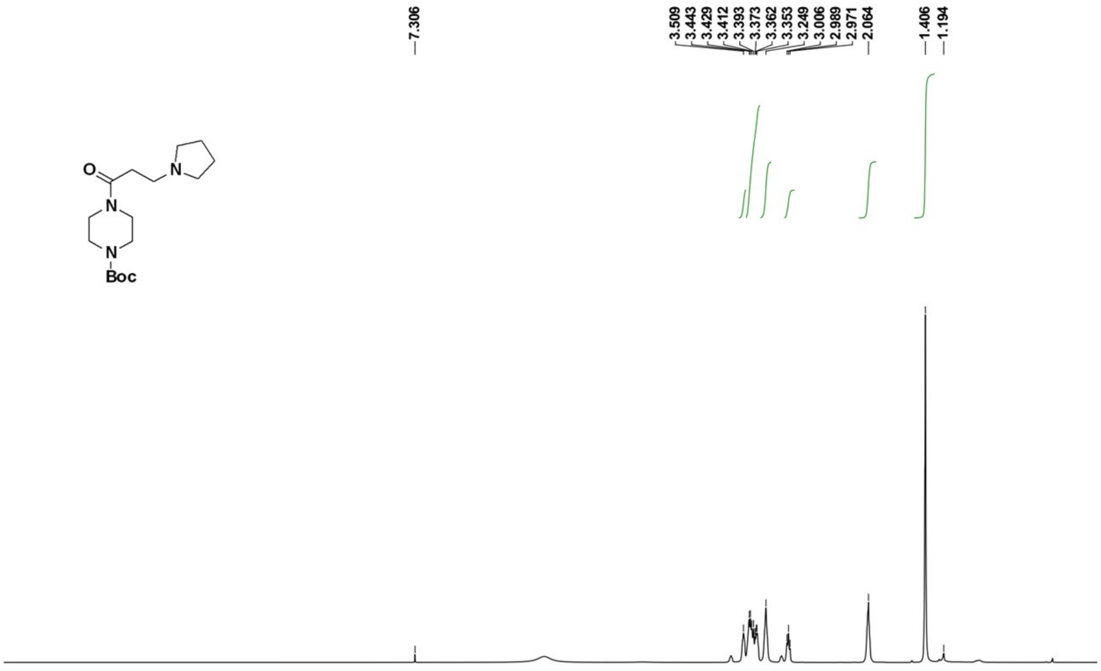

^{13}C NMR spectra of **3fb**

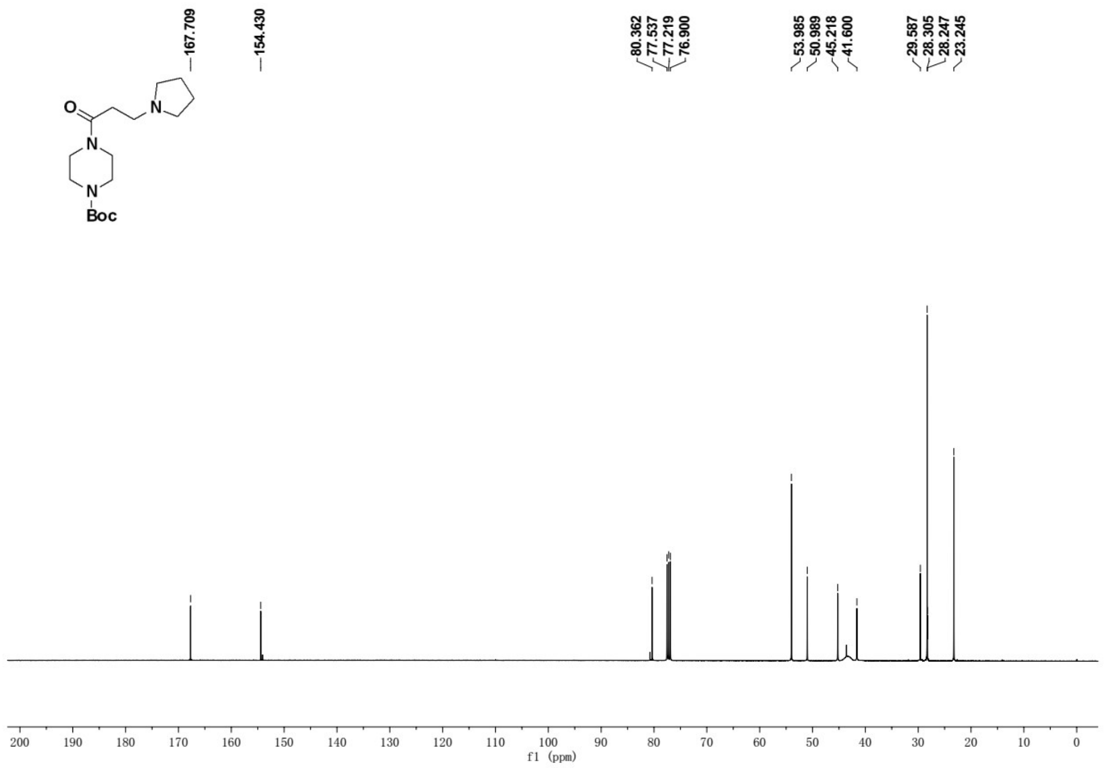

^{1}H NMR spectra of **3fc**

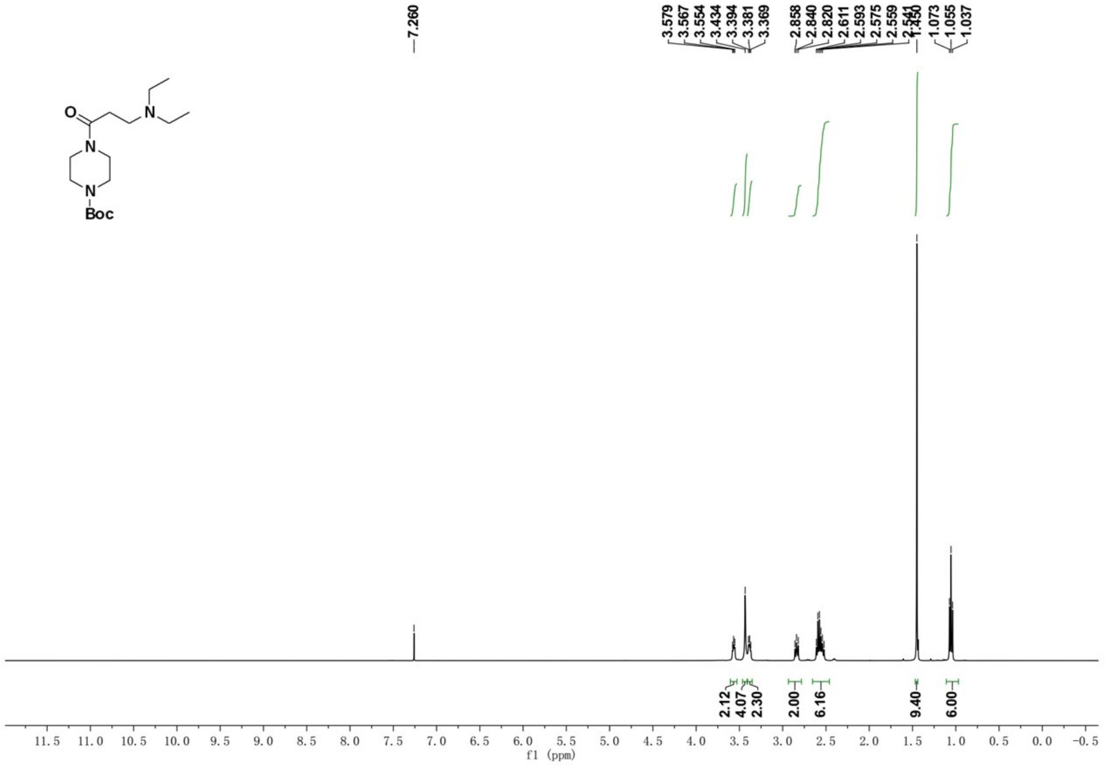

^{13}C NMR spectra of **3fc**

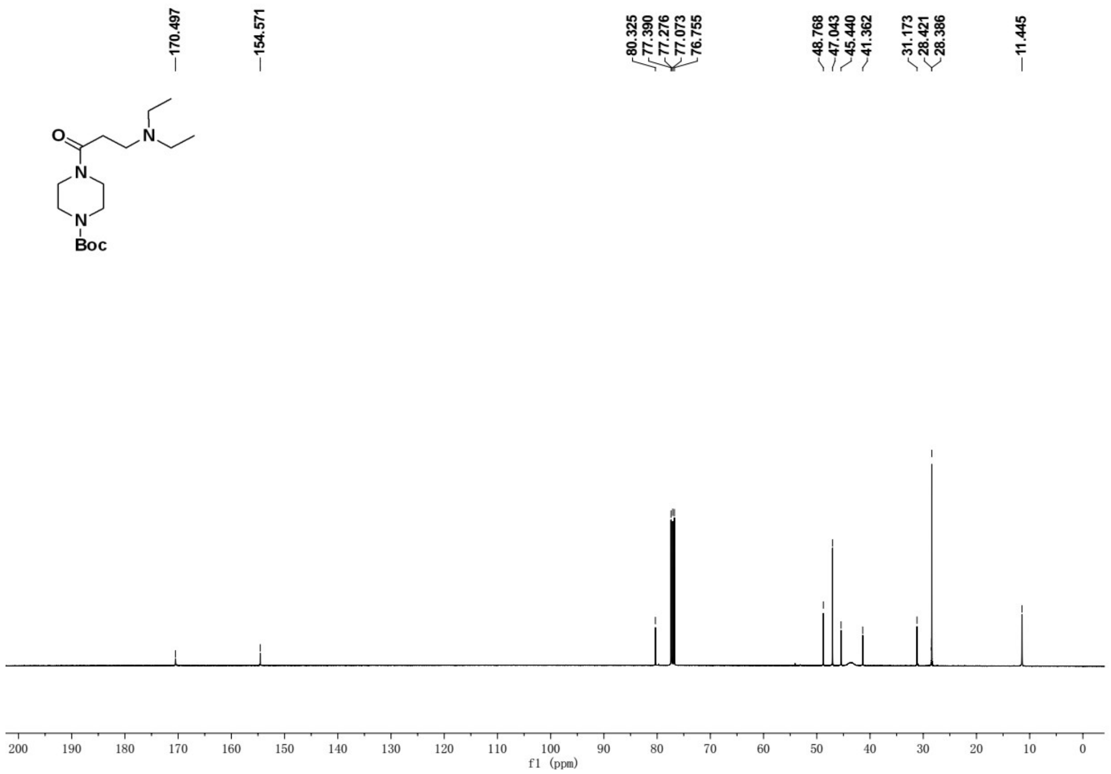

^{1}H NMR spectra of **3fd**

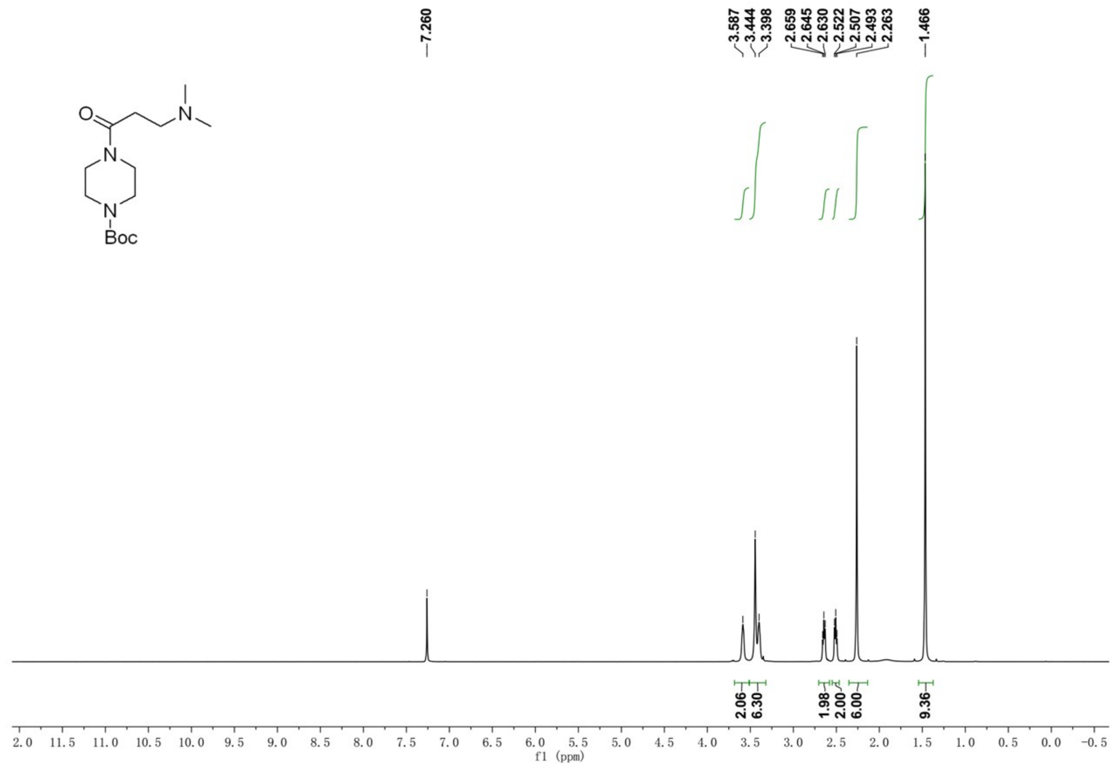

^{13}C NMR spectra of **3fd**

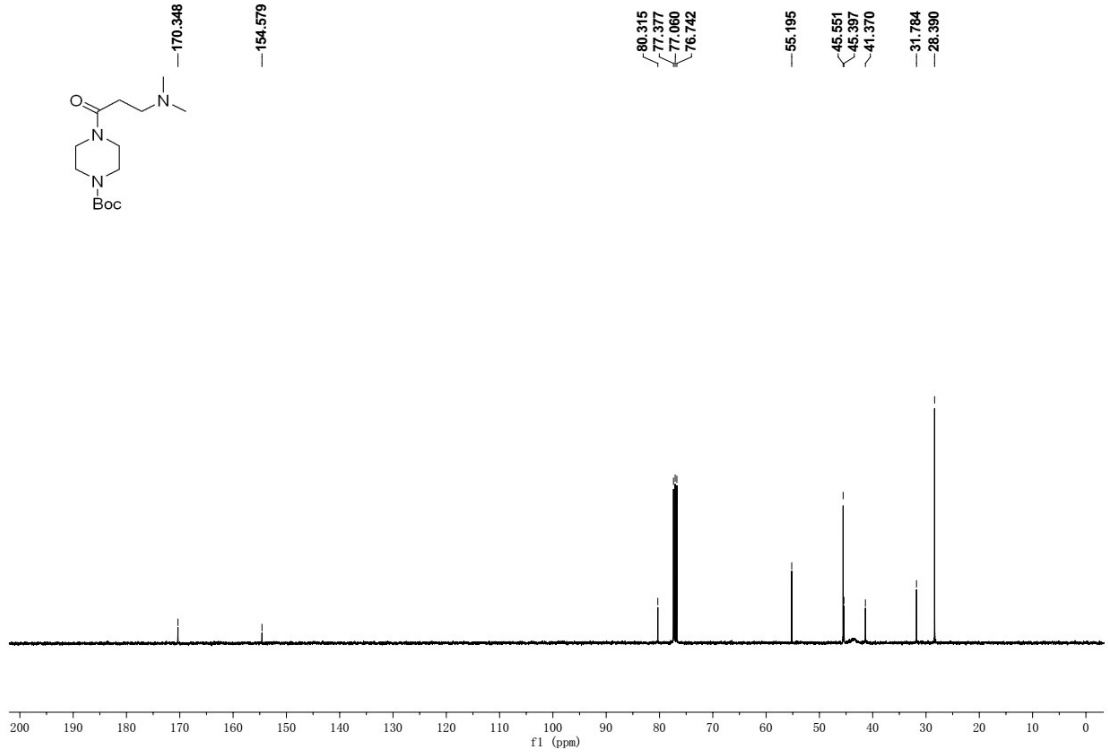

^{1}H NMR spectra of **5ad**

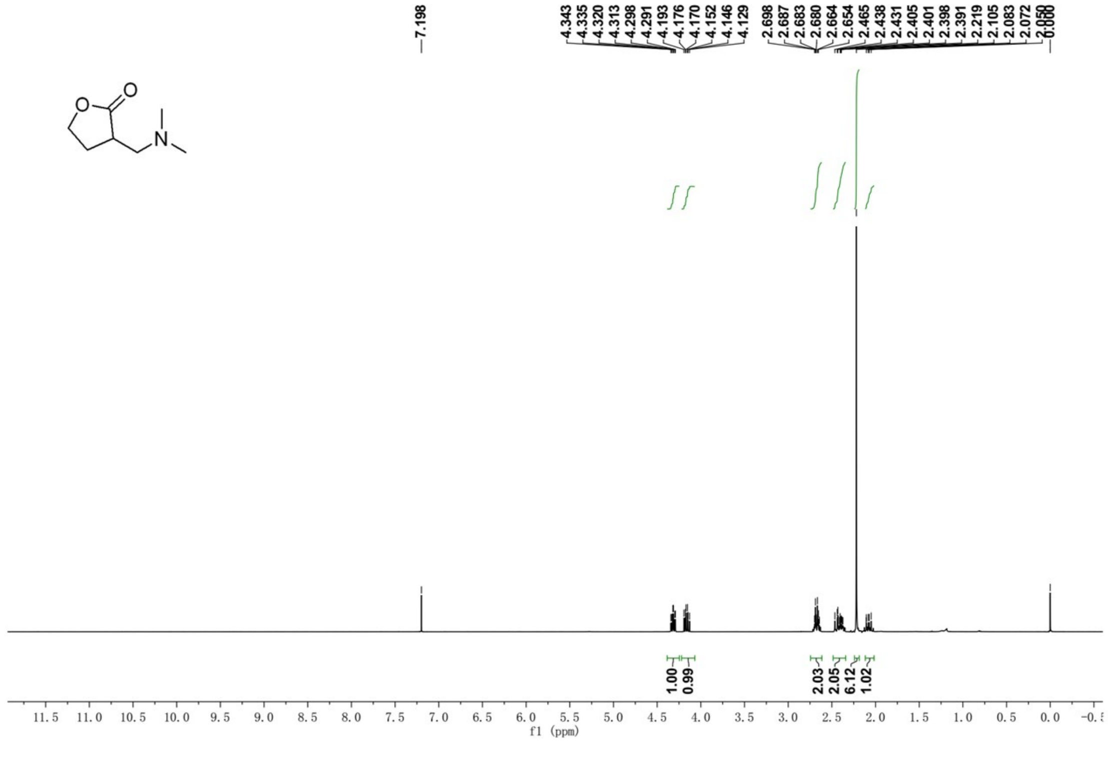

^{13}C NMR spectra of **5ad**

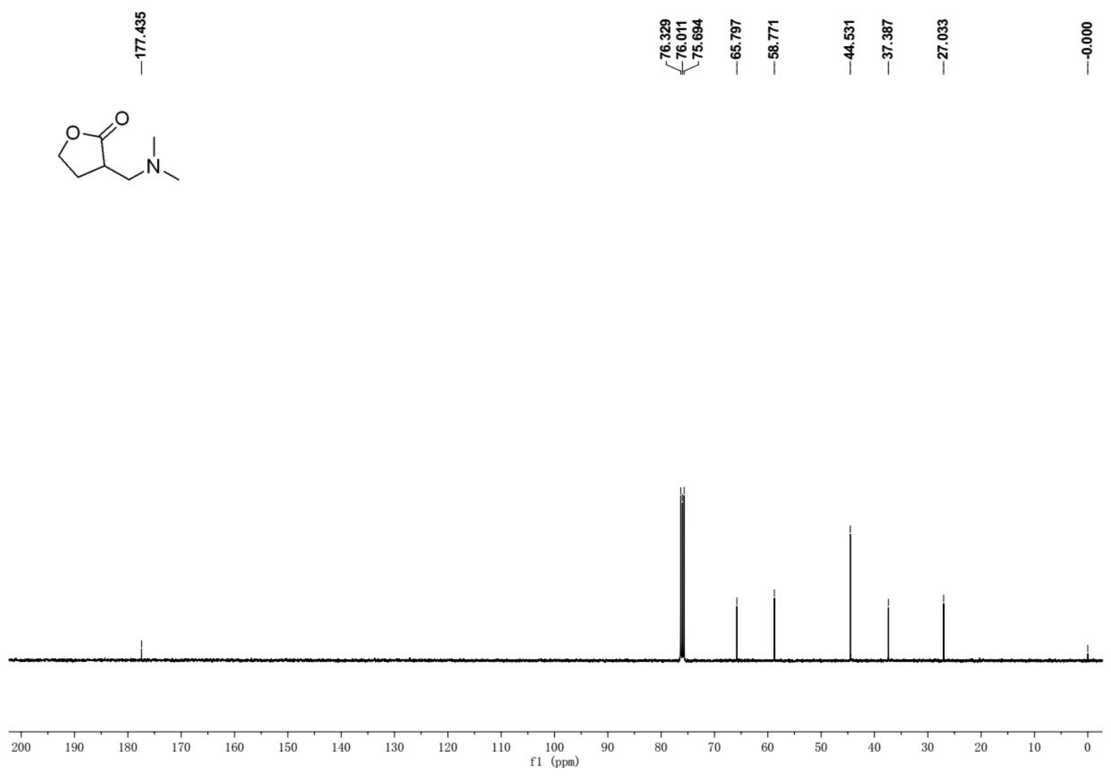

^{1}H NMR spectra of **5ba**

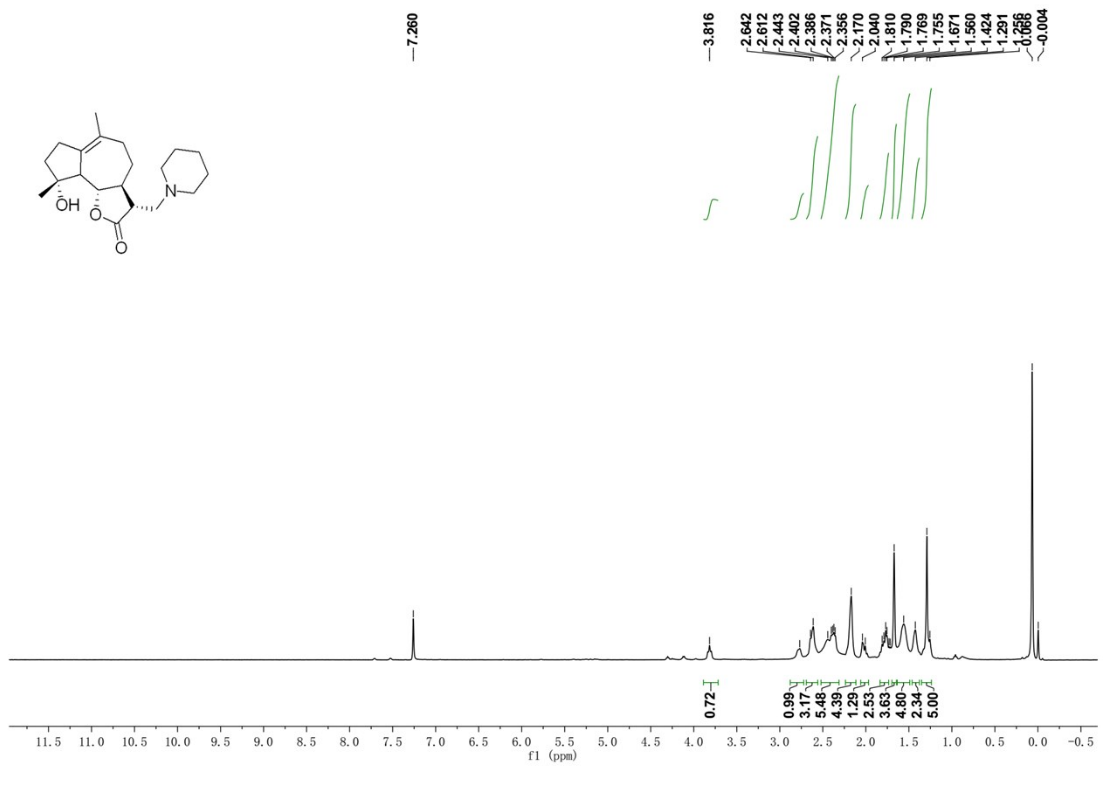

^{13}C NMR spectra of **5ba**

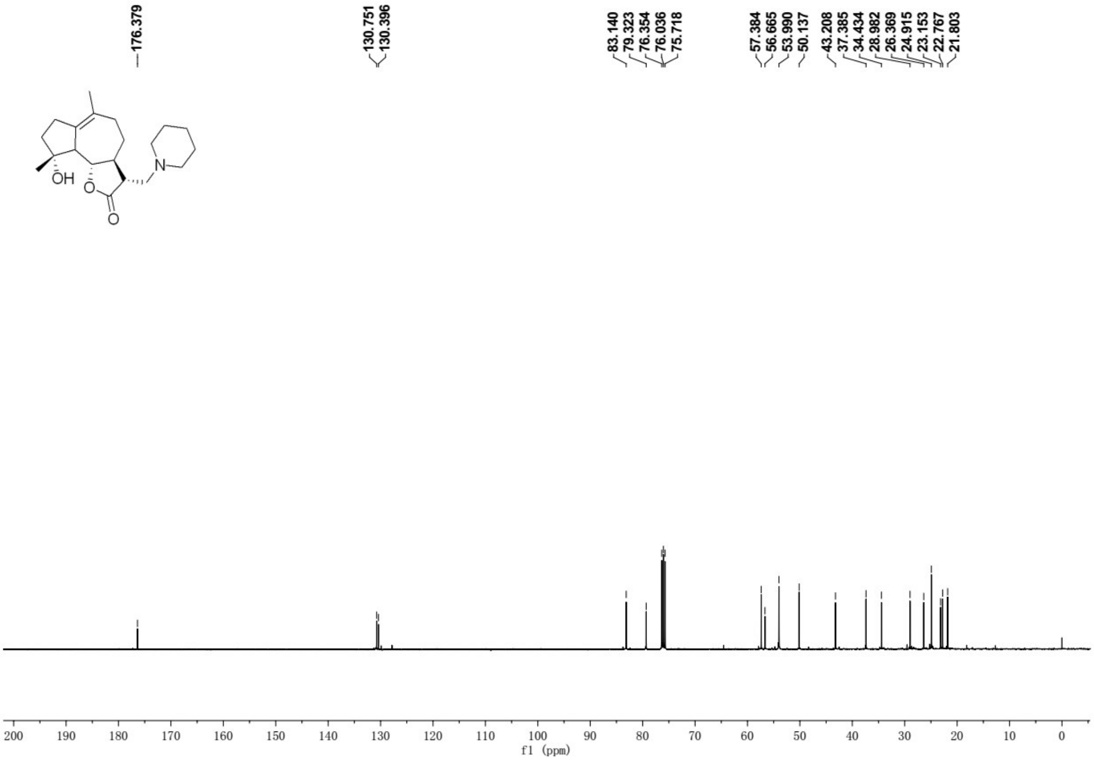

^{1}H NMR spectra of **5bb**

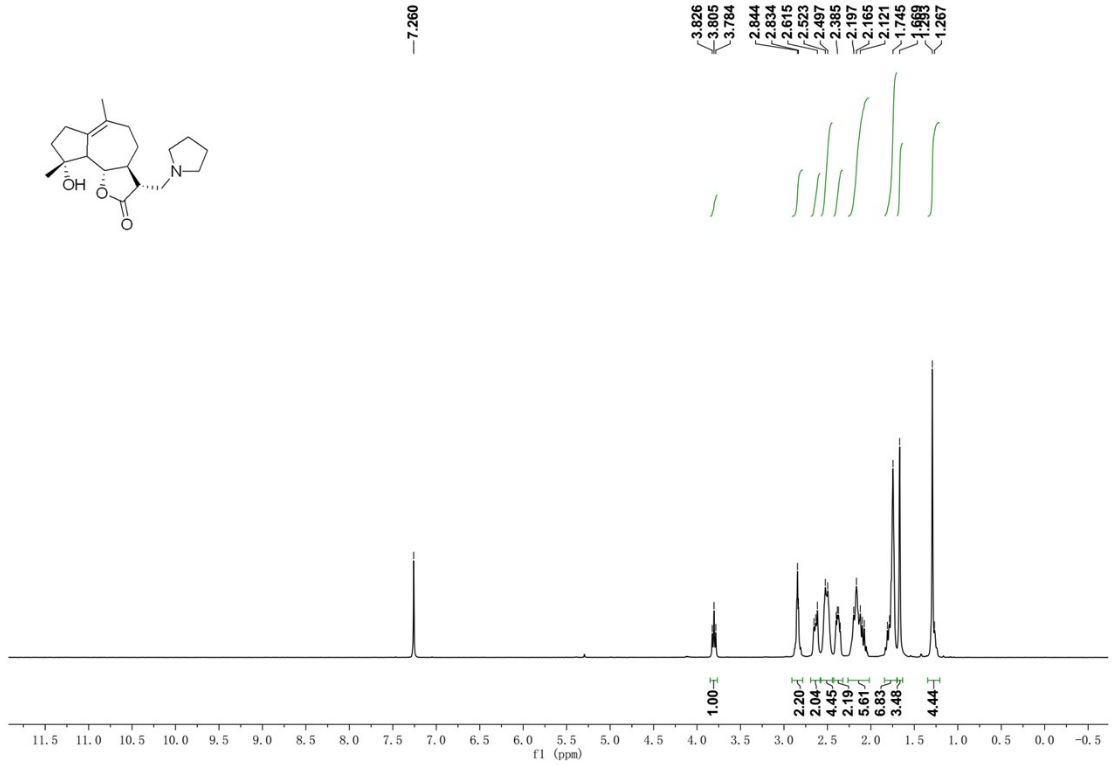

^{13}C NMR spectra of **5bb**

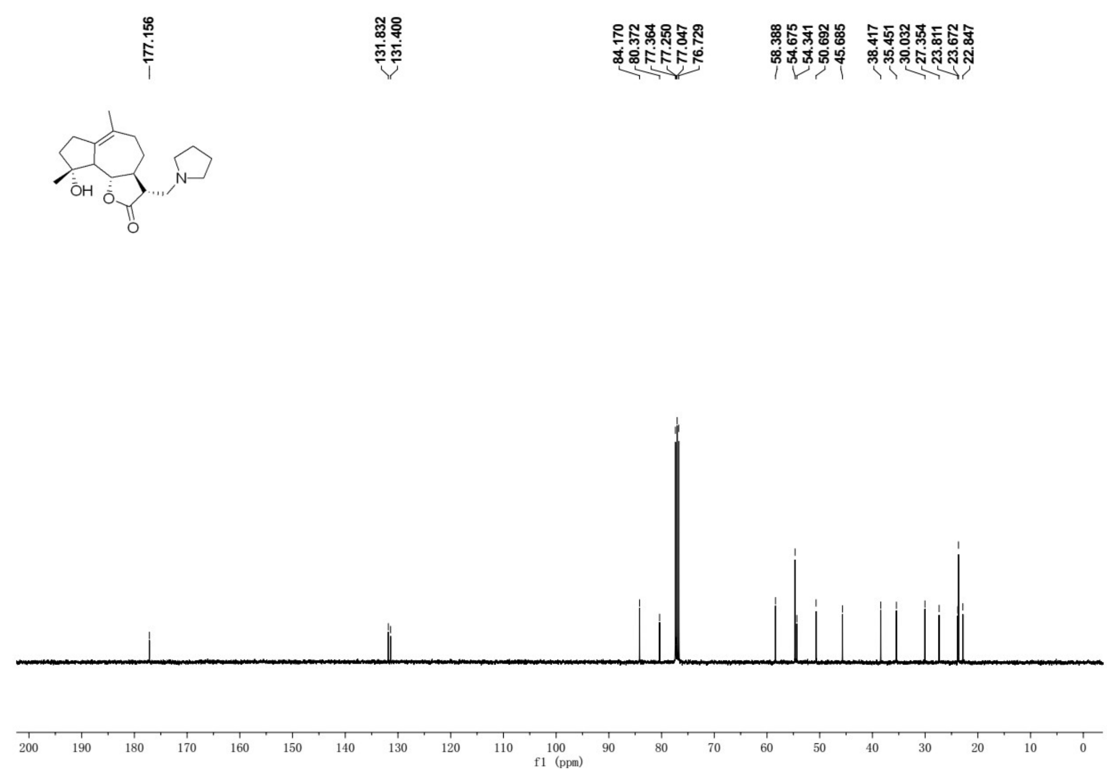

^{1}H NMR spectra of **5bd**

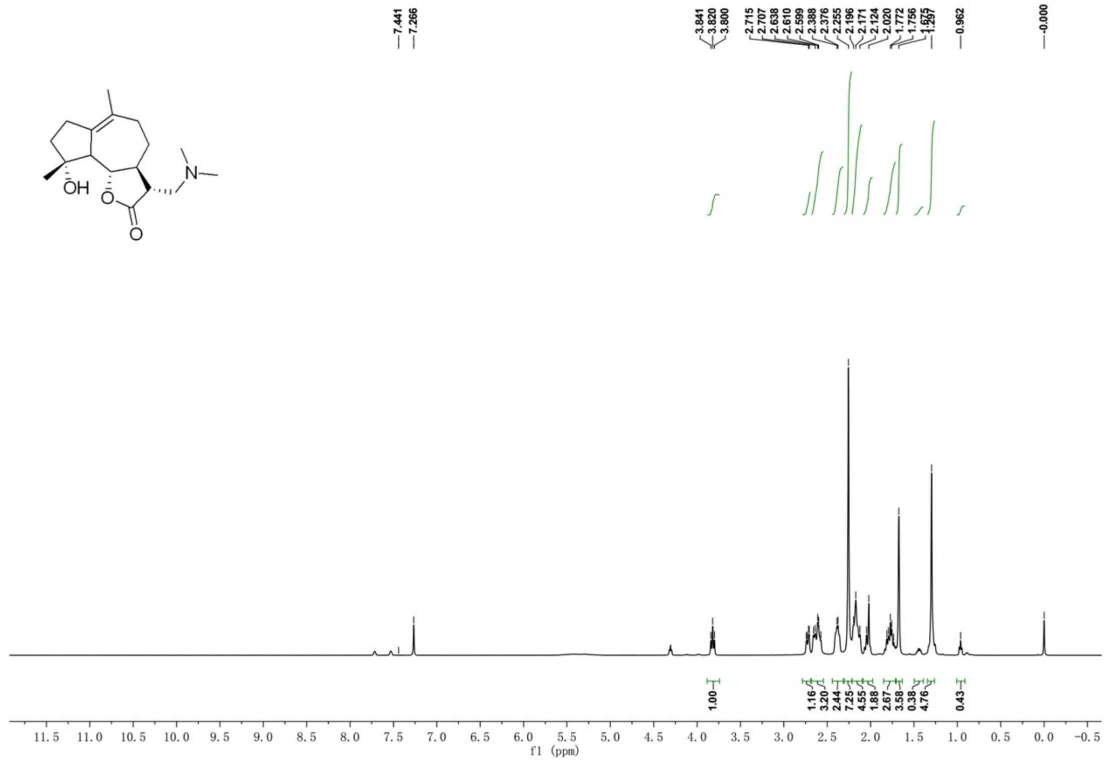

^{13}C NMR spectra of **5bd**

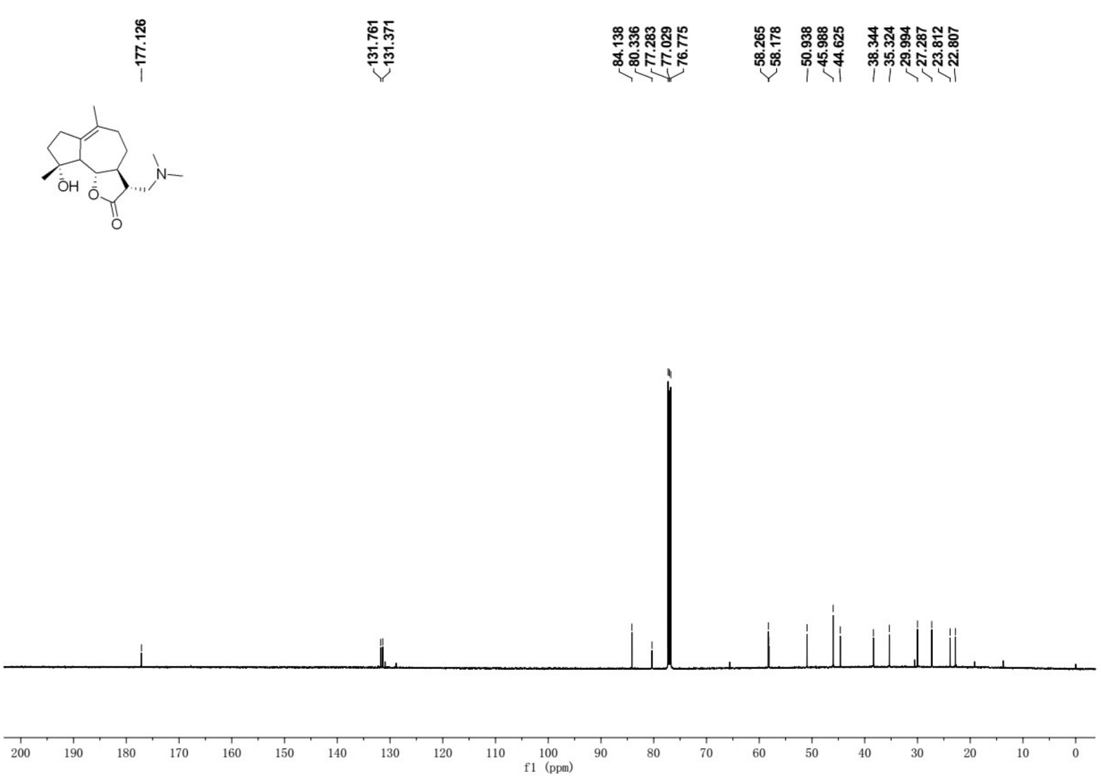

^{1}H NMR spectra of **5be**

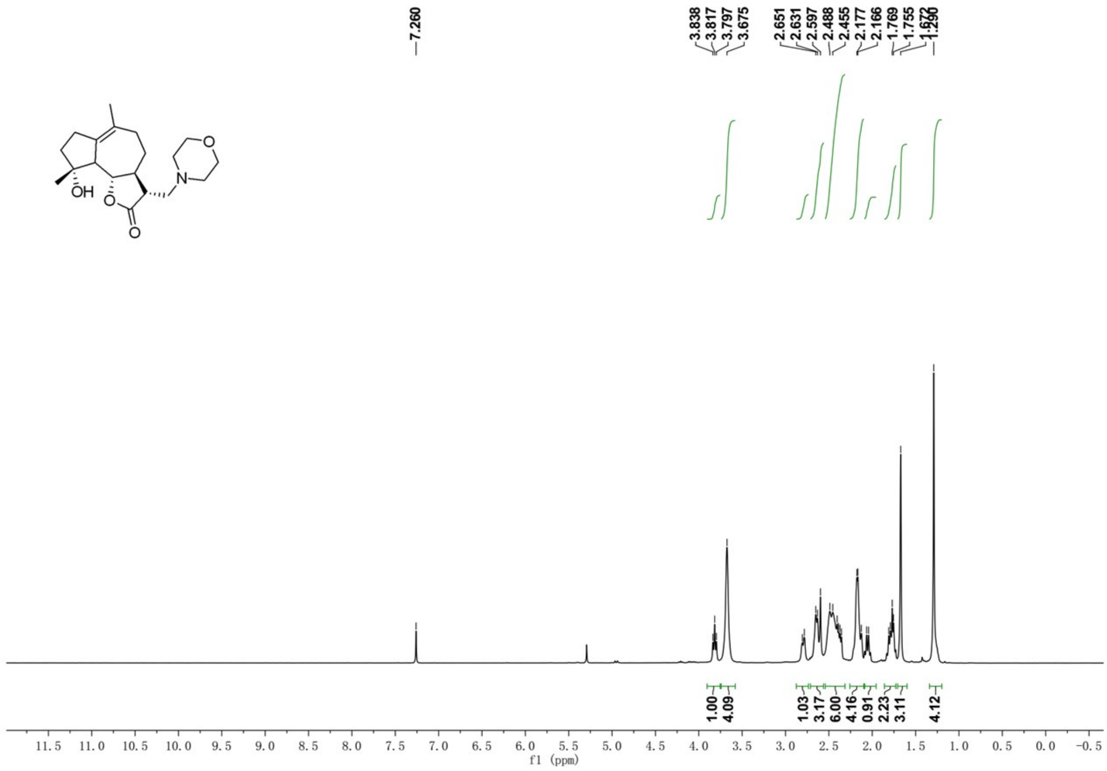

^{13}C NMR spectra of **5be**

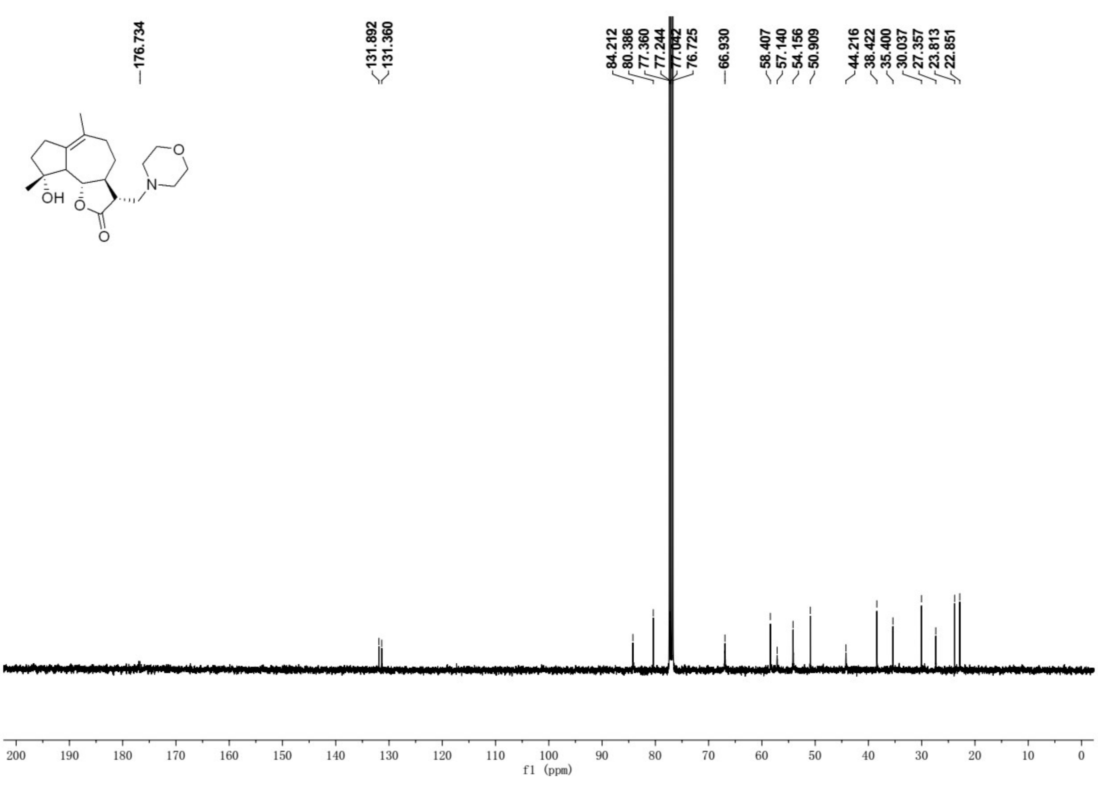

^{1}H NMR spectra of **5bh**

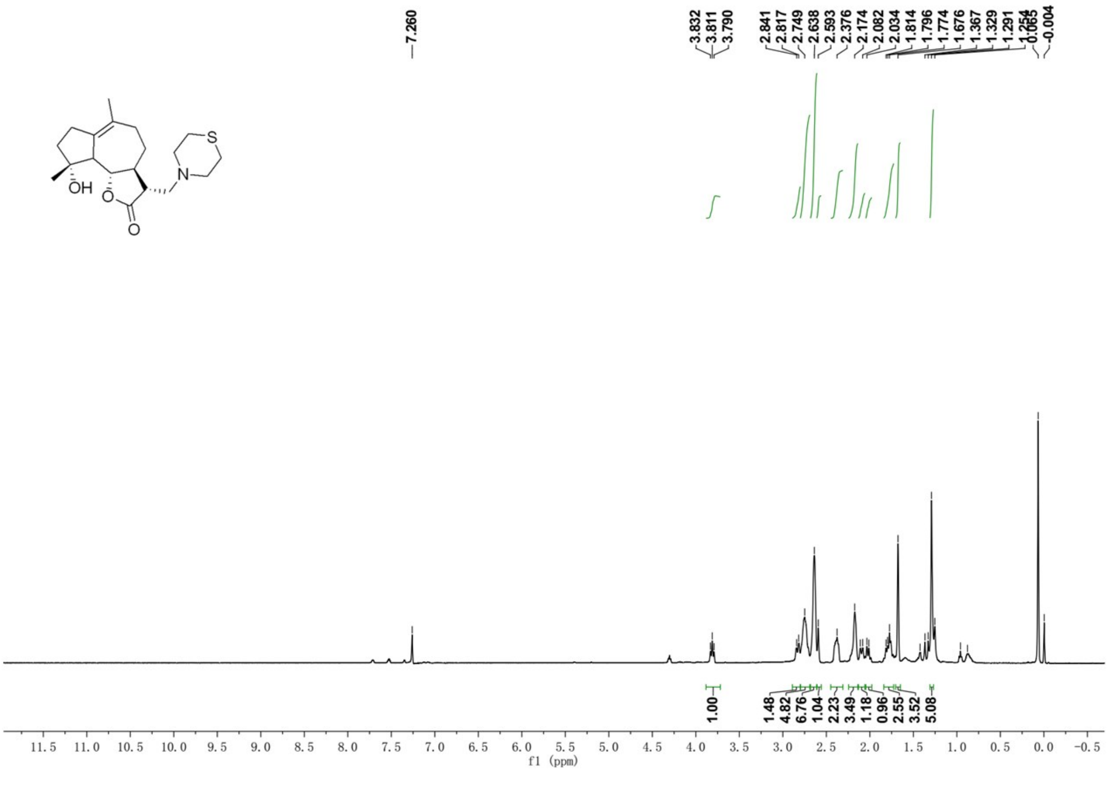

^{13}C NMR spectra of **5bh**

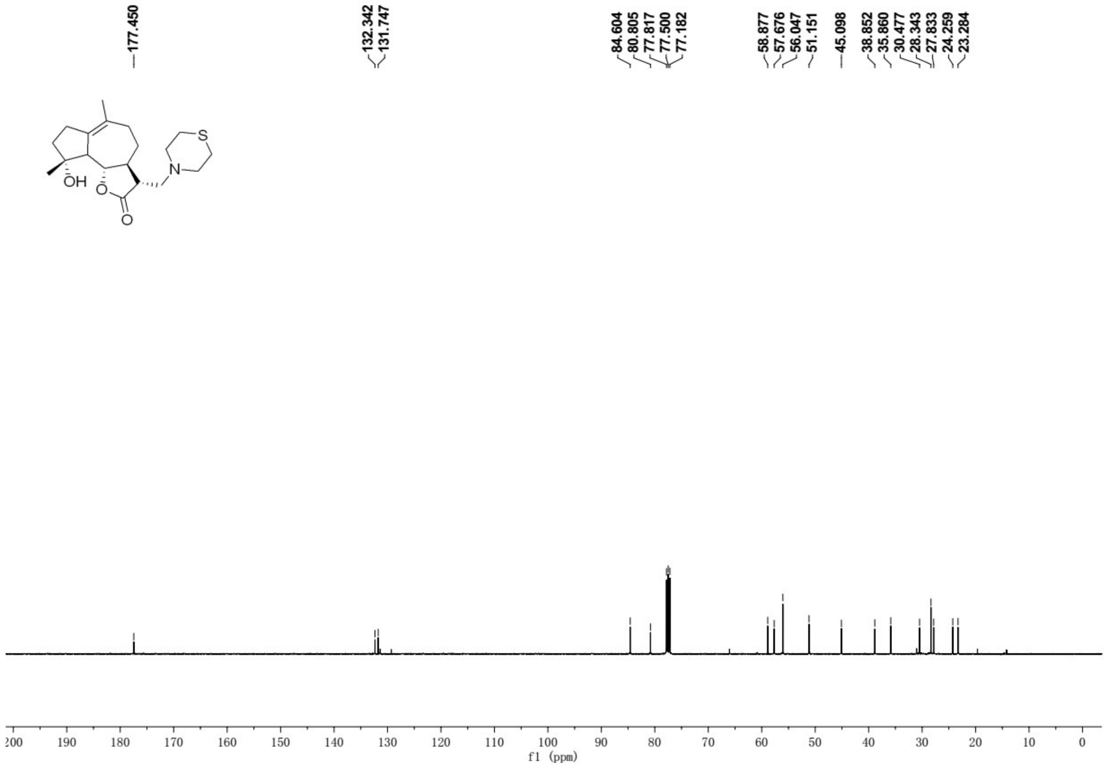

^{1}H NMR spectra of **5bi**

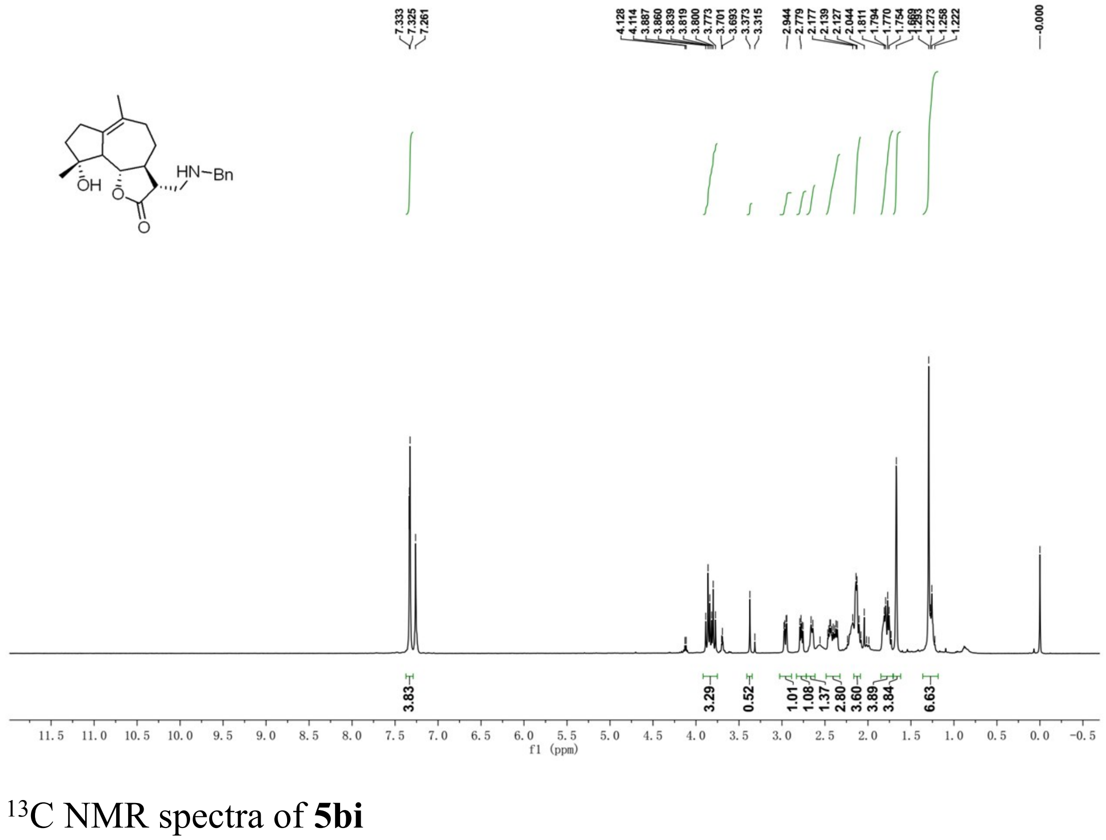

^{13}C NMR spectra of **5bi**

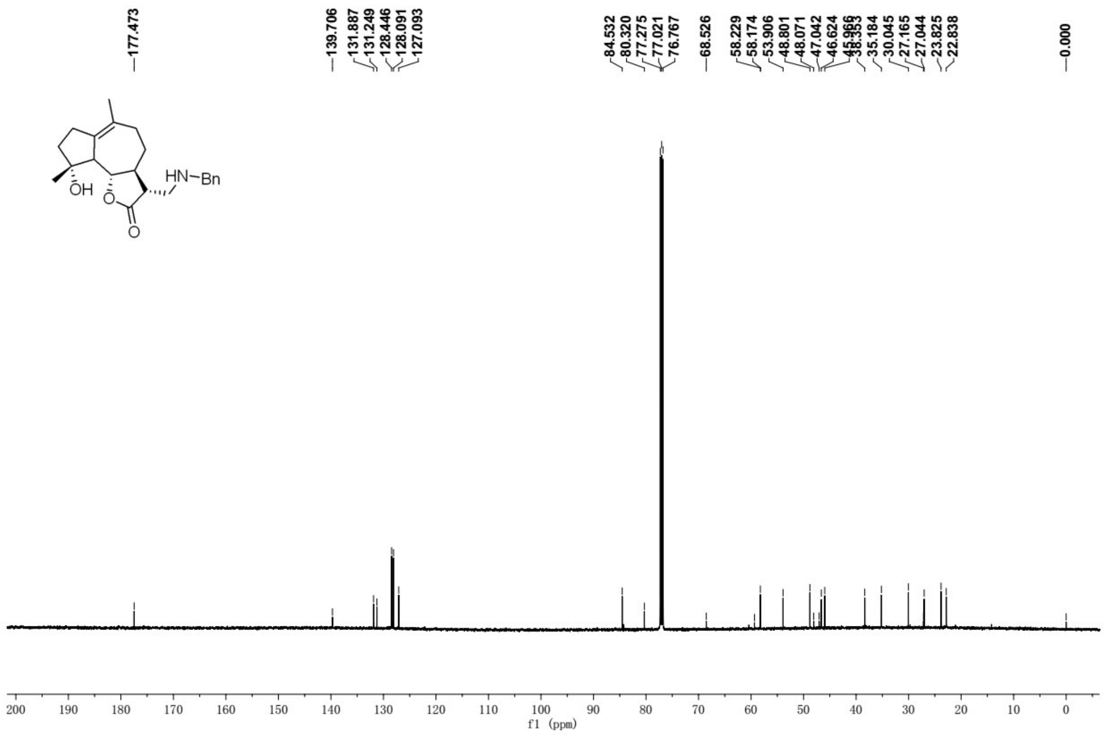

^{1}H NMR spectra of **5bf**

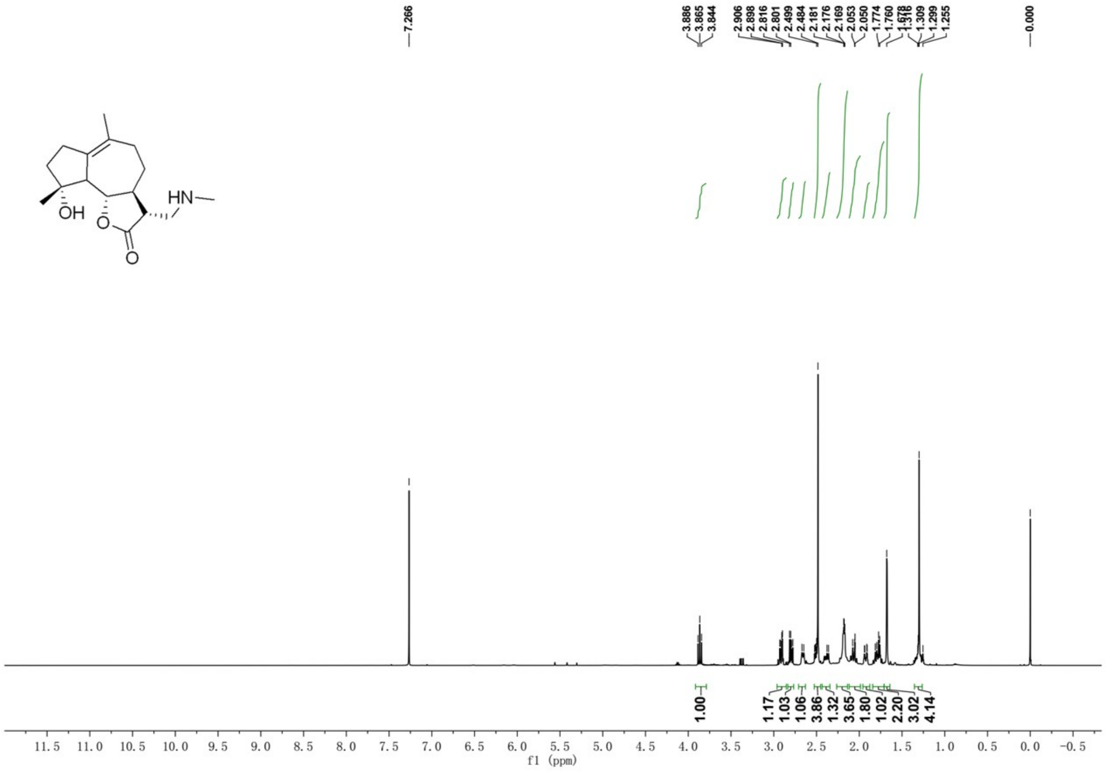

^{13}C NMR spectra of **5bf**

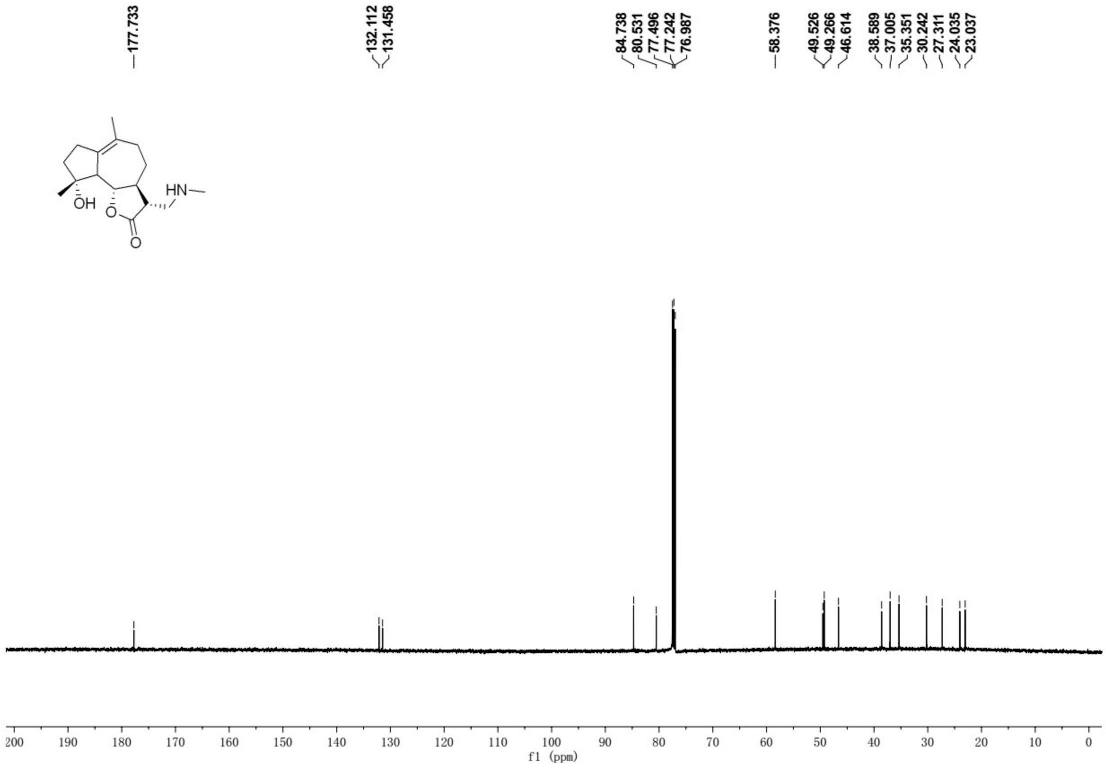

^{1}H NMR spectra of **5bj**

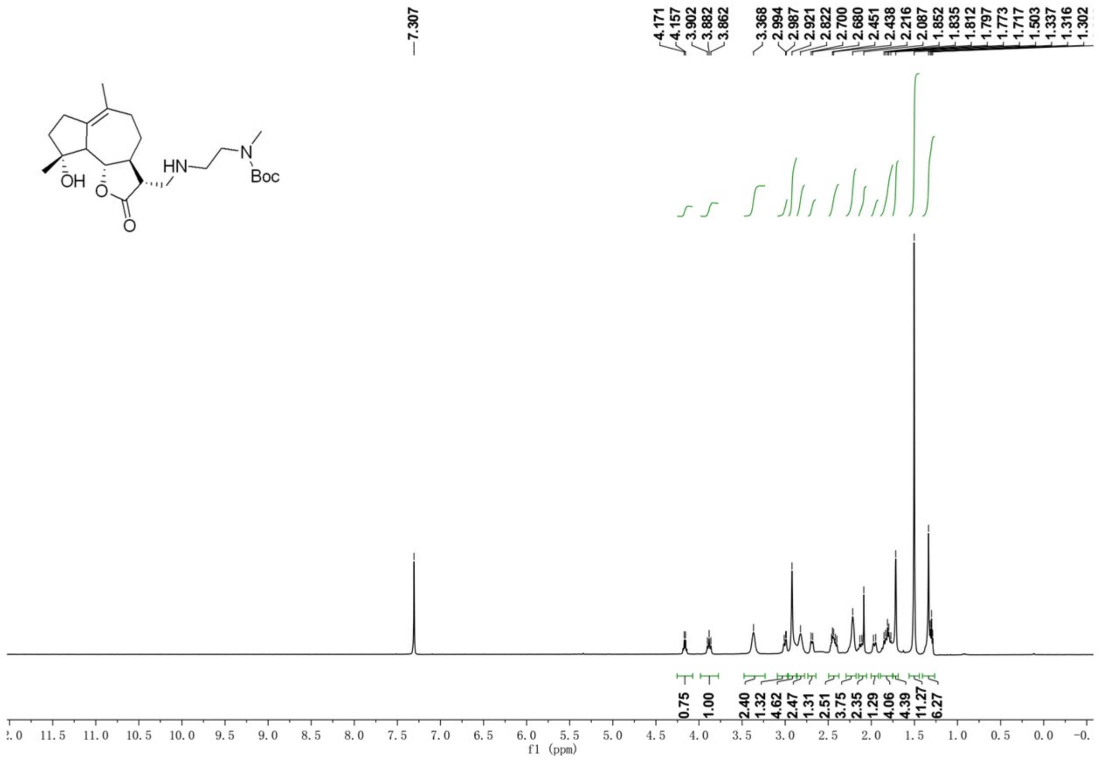

^{13}C NMR spectra of **5bj**

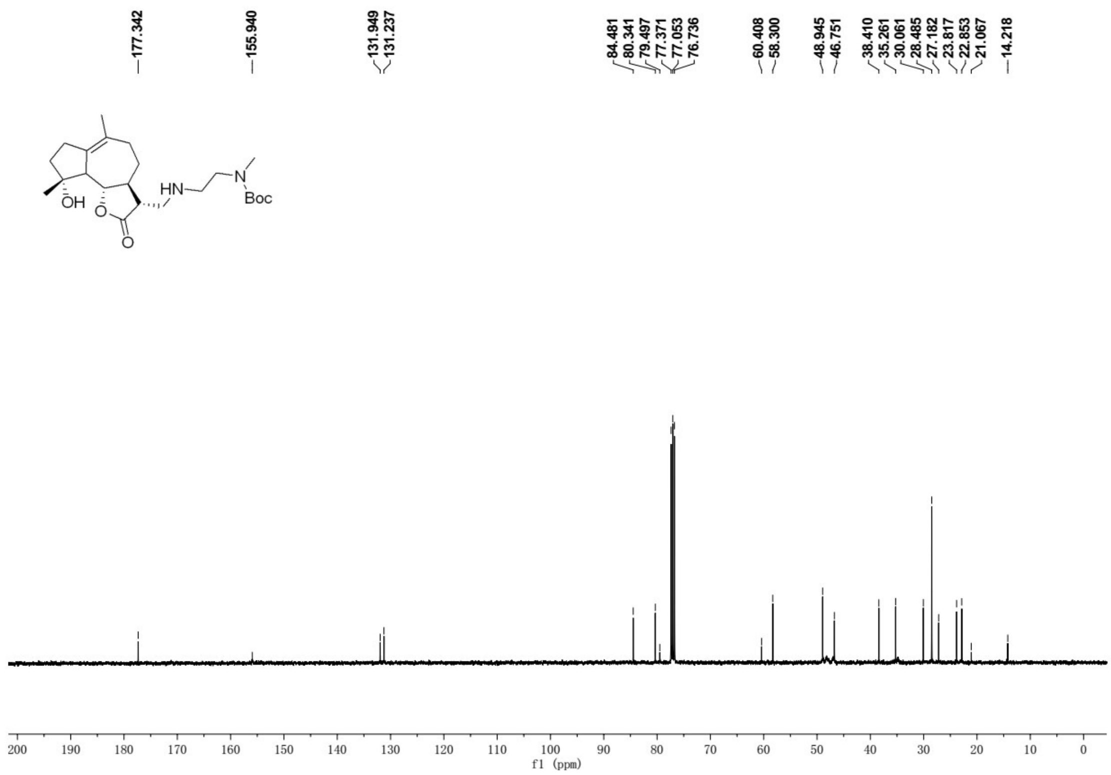

^{1}H NMR spectra of **5cd**

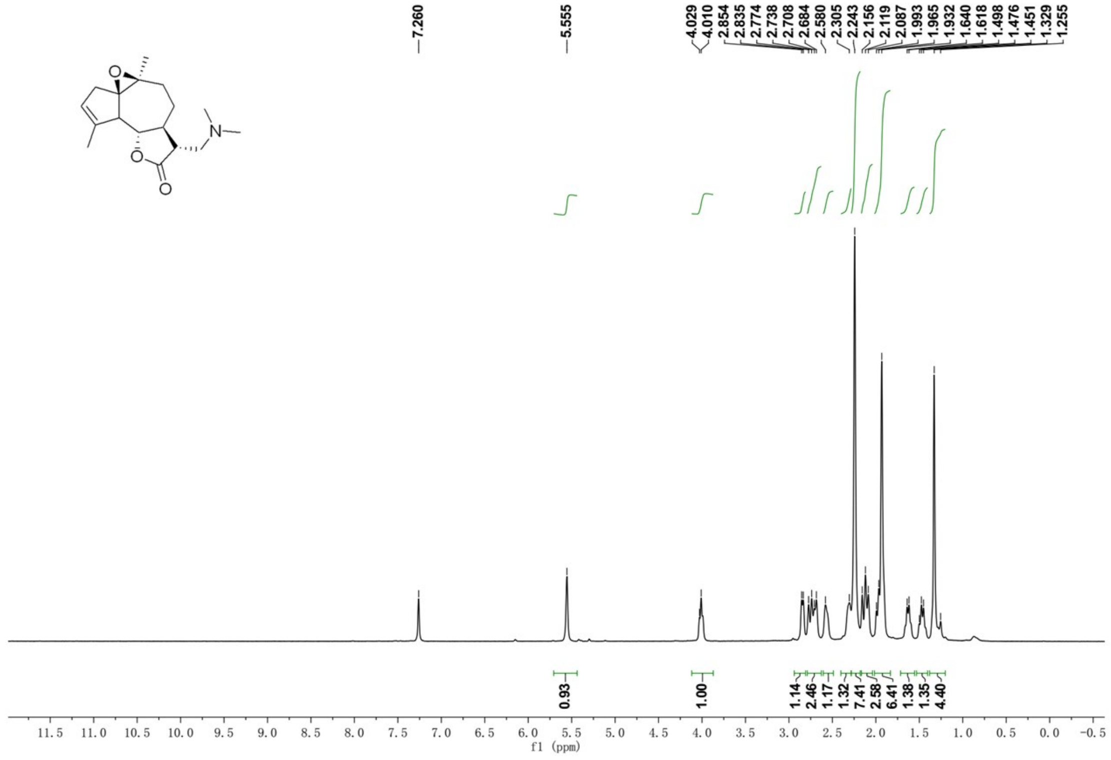

^{13}C NMR spectra of **5cd**

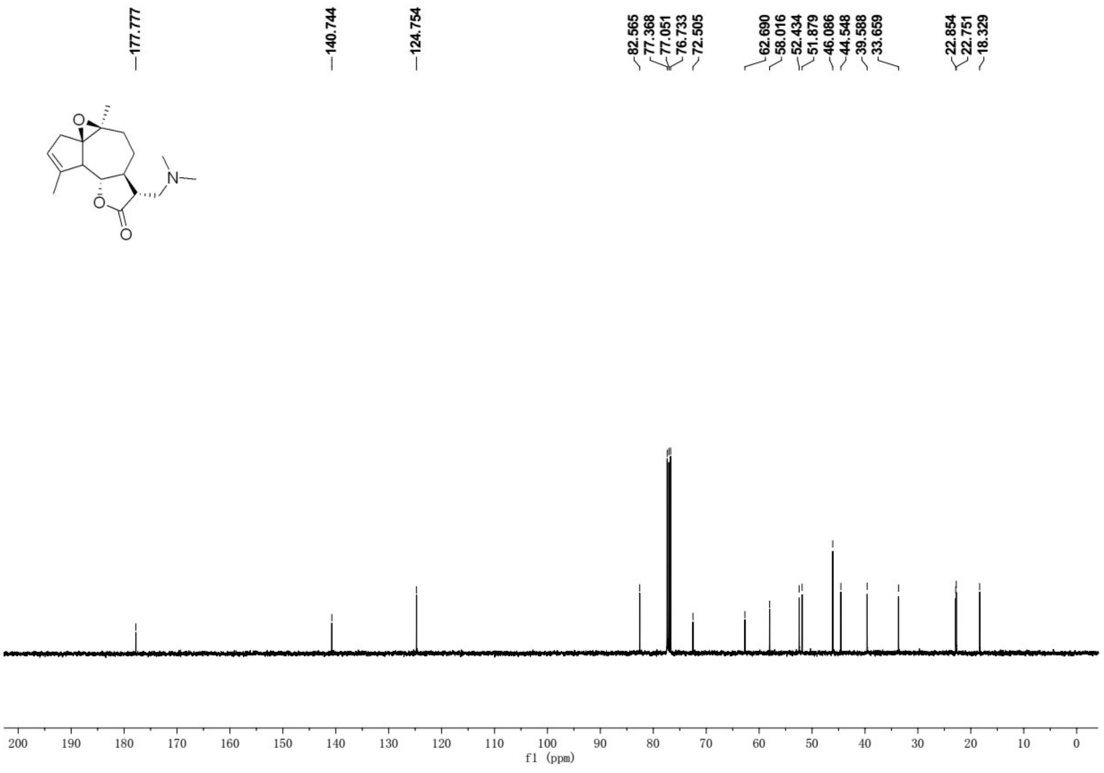

^{1}H NMR spectra of **5dd**

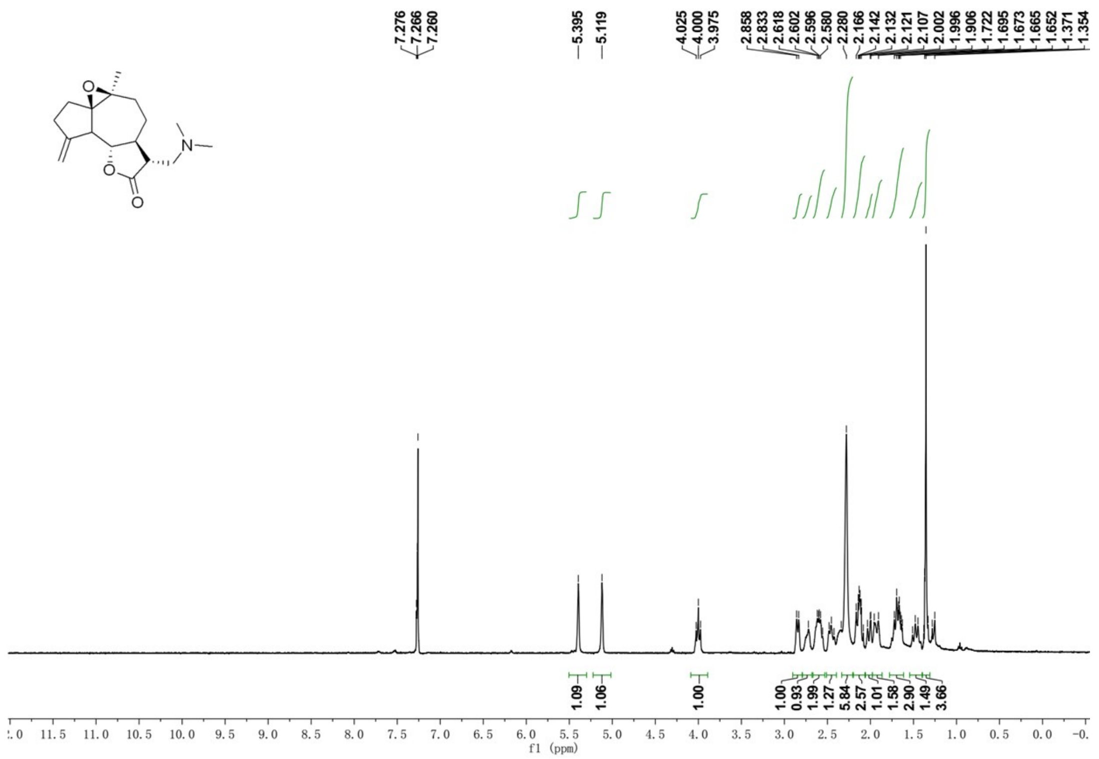

^{13}C NMR spectra of **5dd**

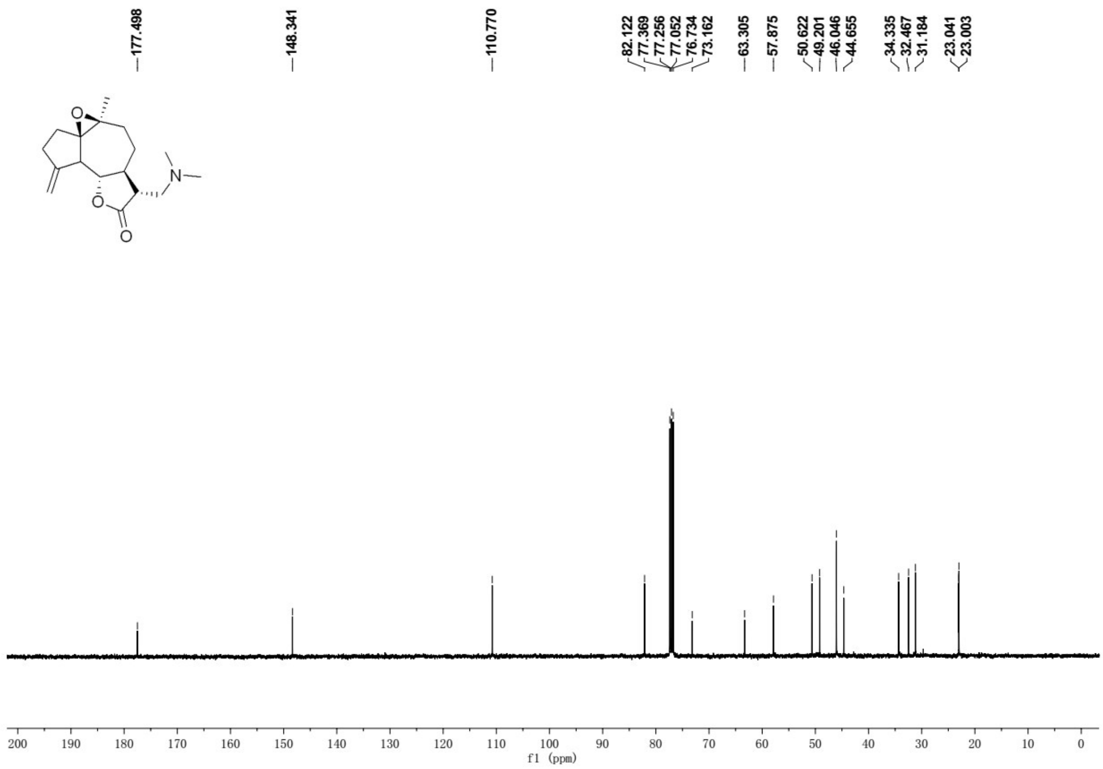

^{1}H NMR spectra of **5ed**

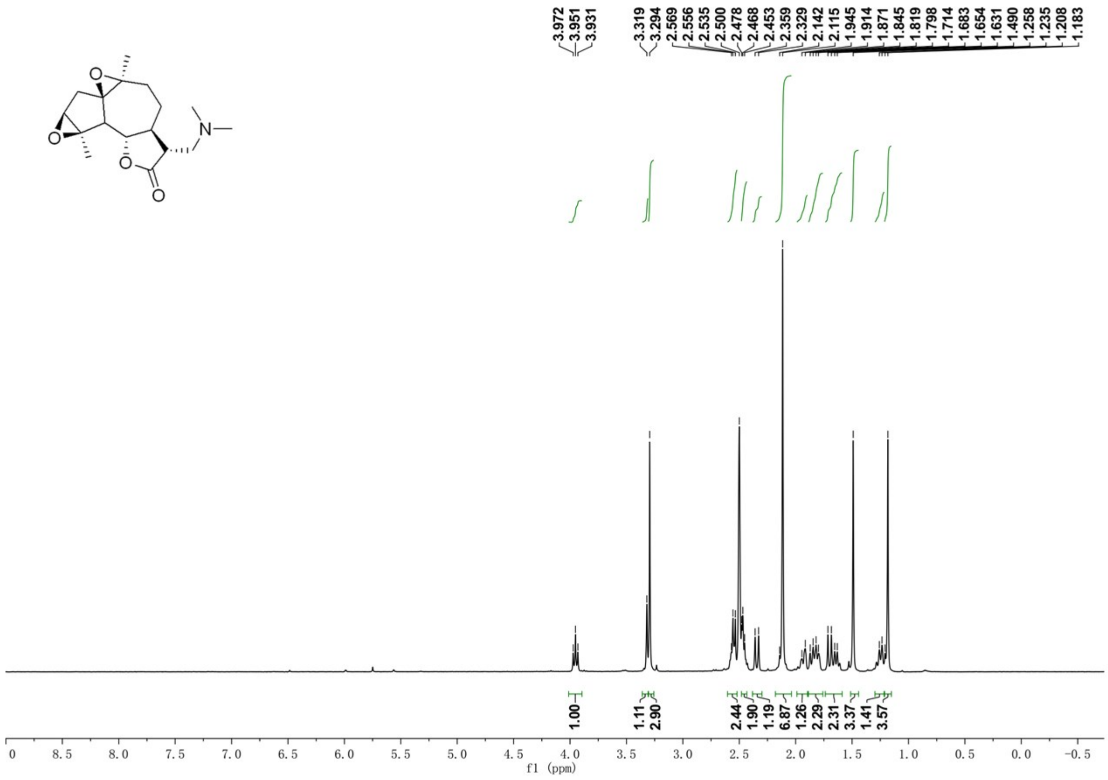

^{13}C NMR spectra of **5ed**

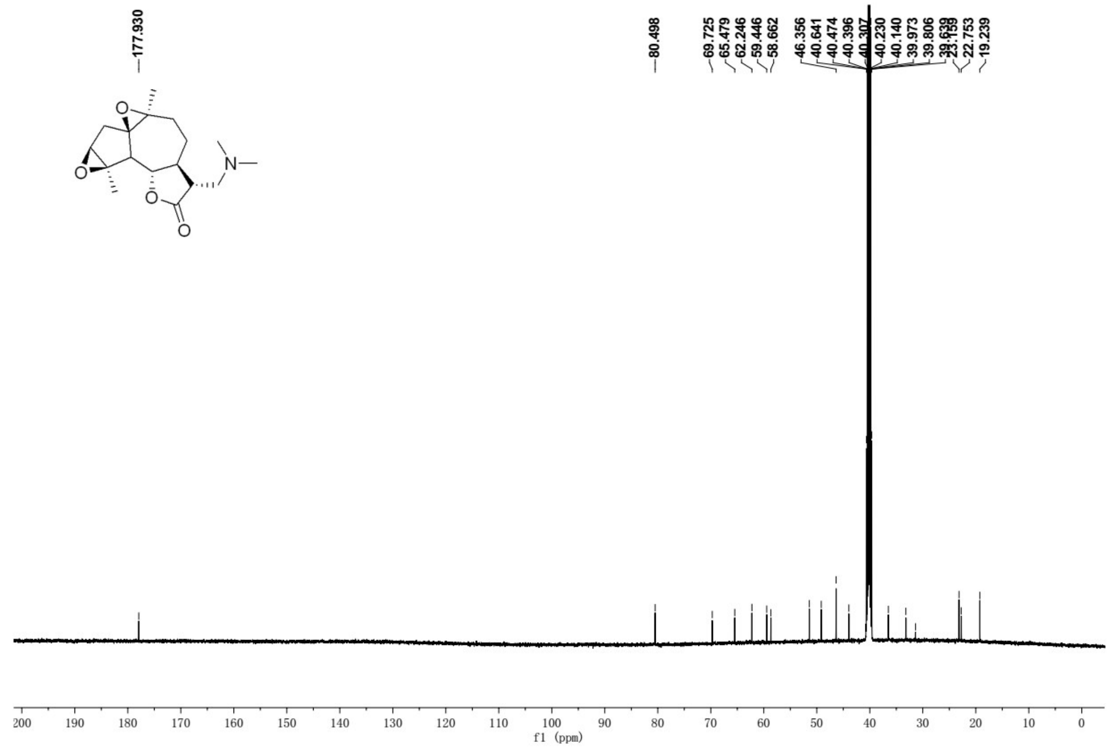

^{1}H NMR spectra of **5fd**

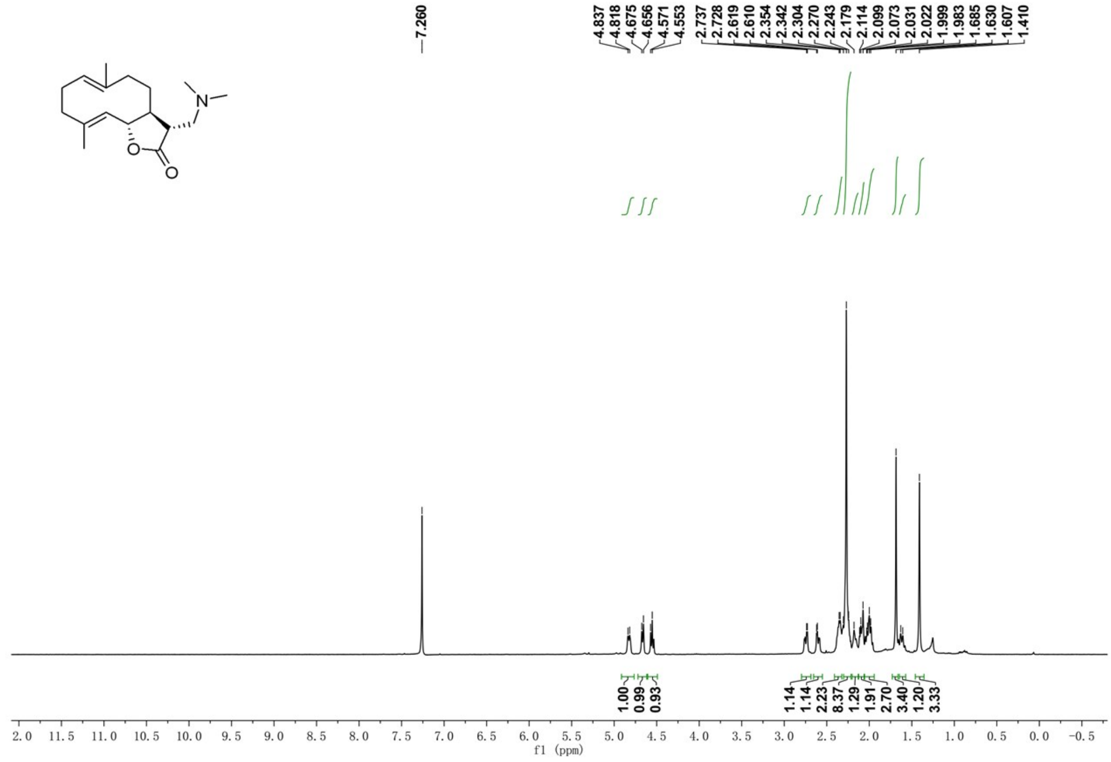

^{13}C NMR spectra of **5fd**

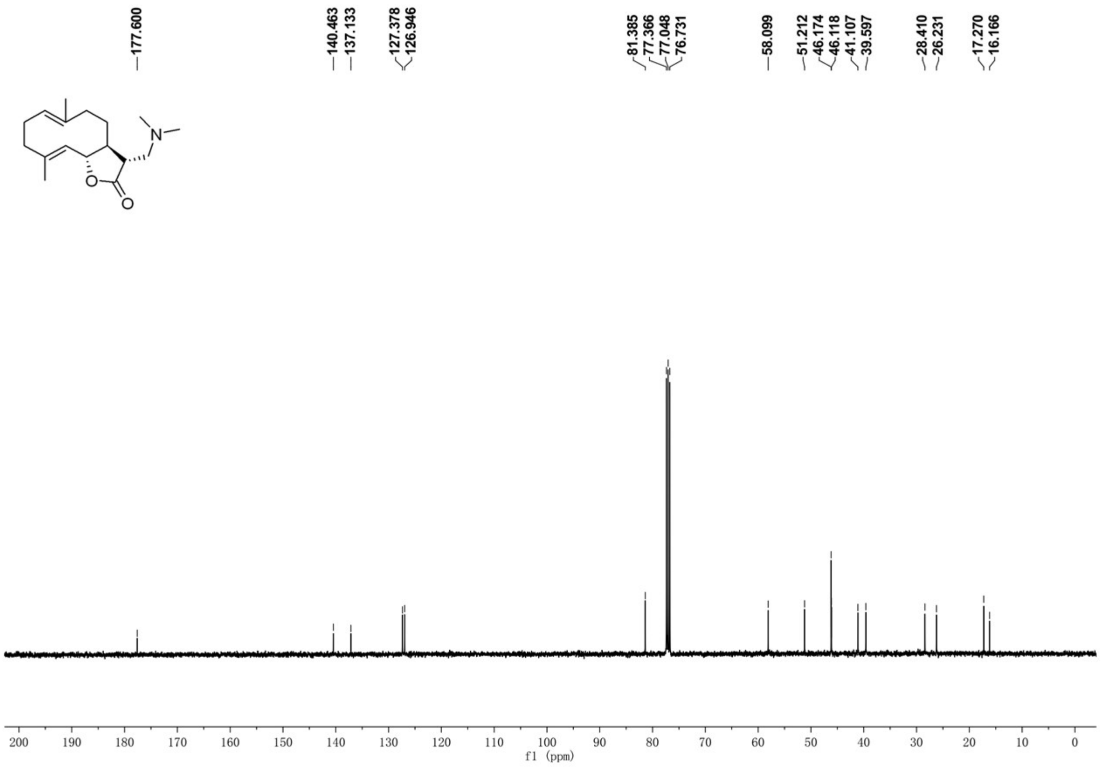

^{1}H NMR spectra of **5gd**

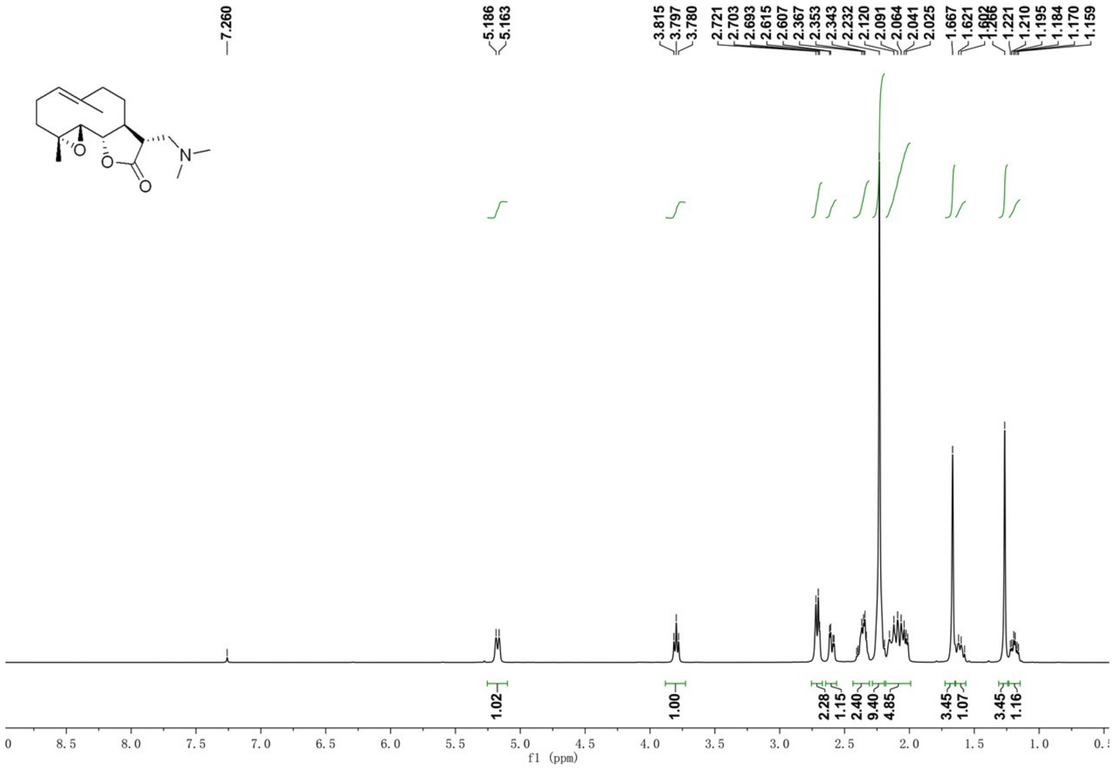

^{13}C NMR spectra of **5gd**

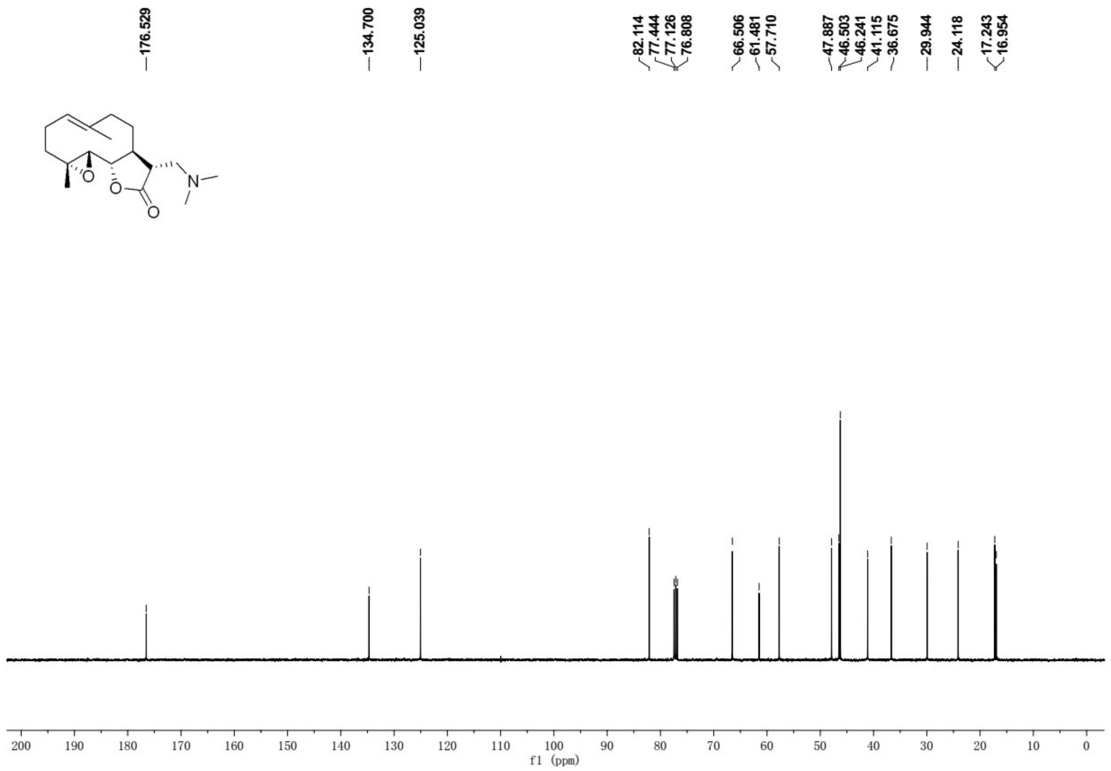

^{1}H NMR spectra of **6a**

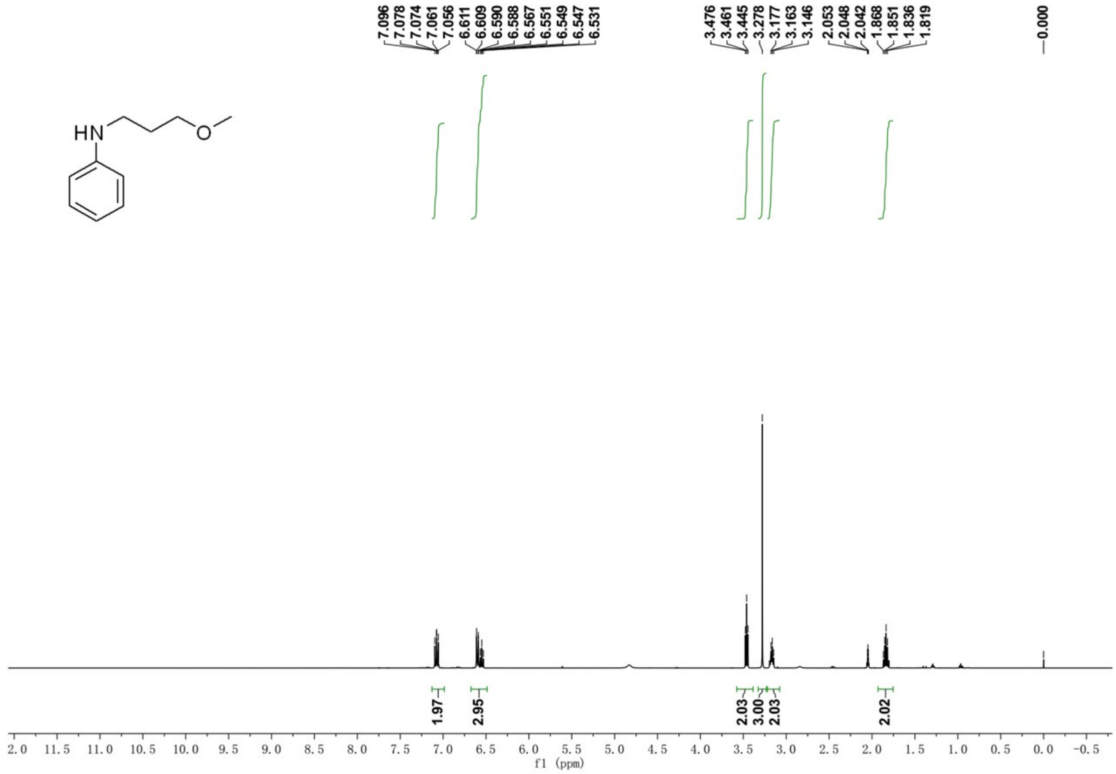

^{1}H NMR spectra of **7a**

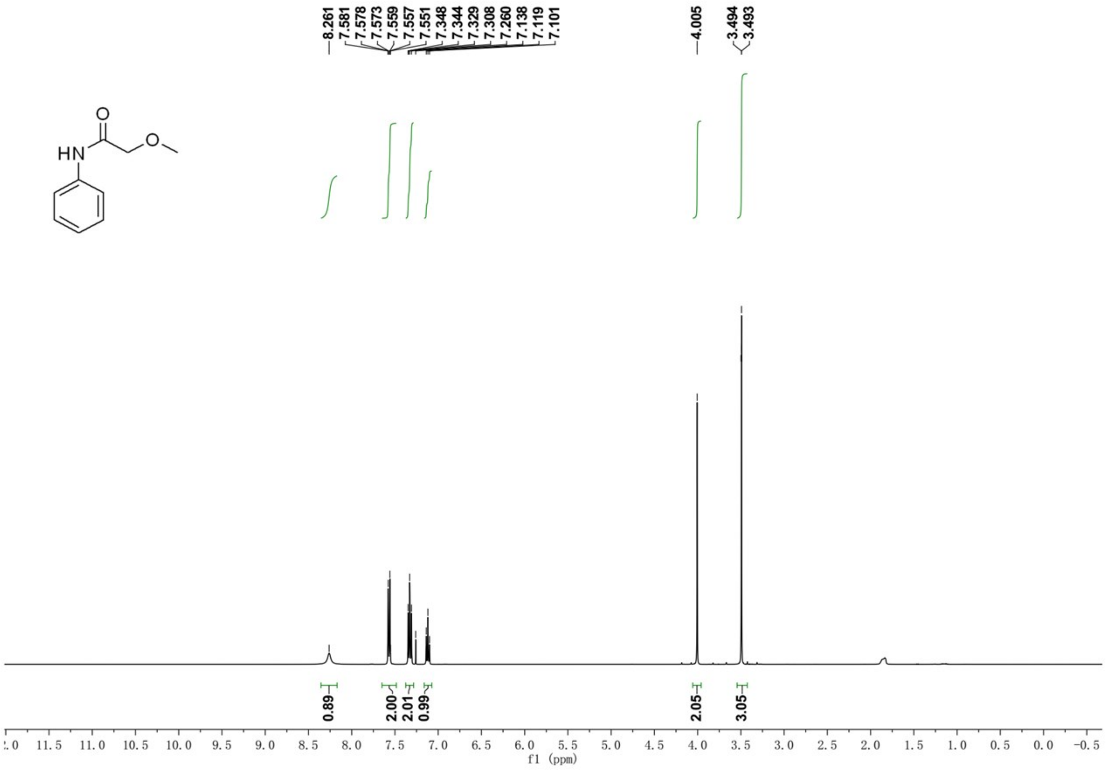

^{1}H NMR spectra of **8a**

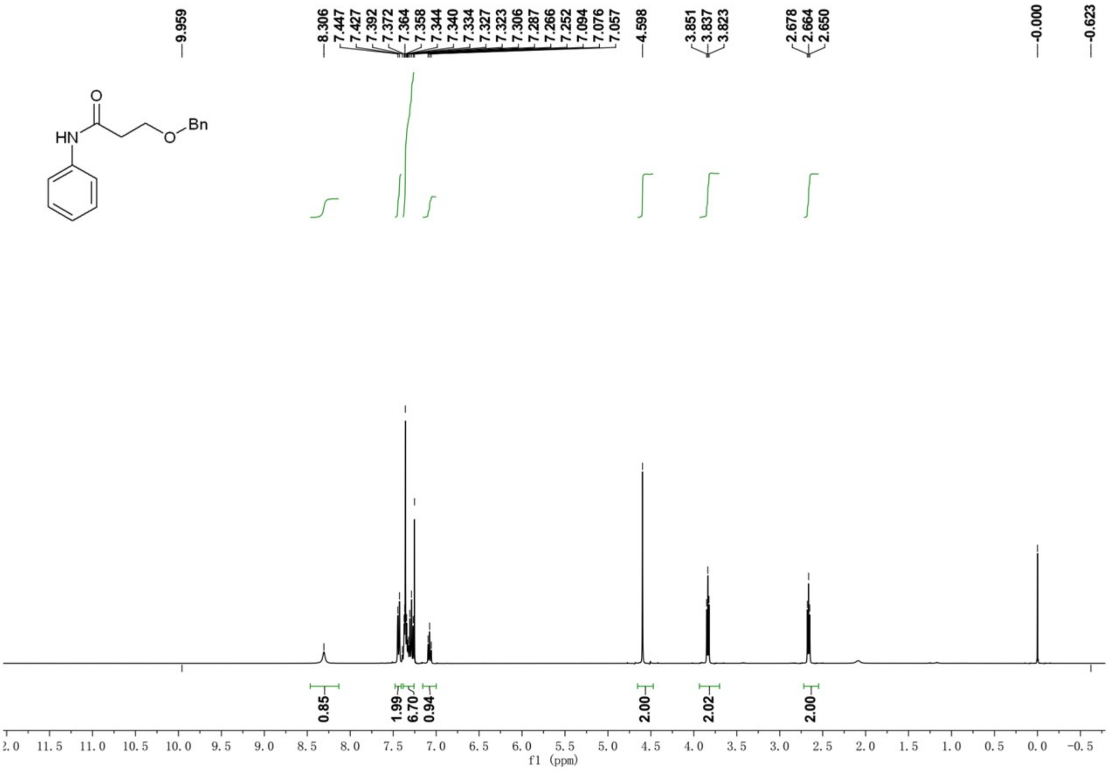

^{1}H NMR spectra of **8b**

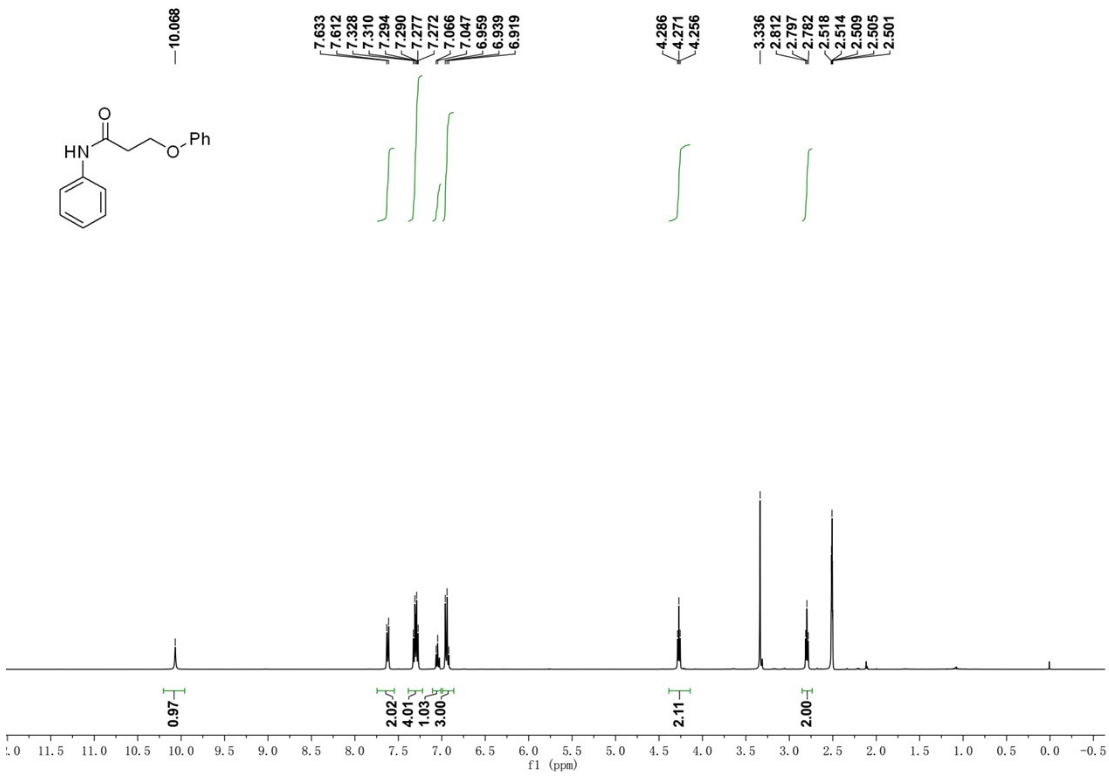

^{1}H NMR spectra of **9a**

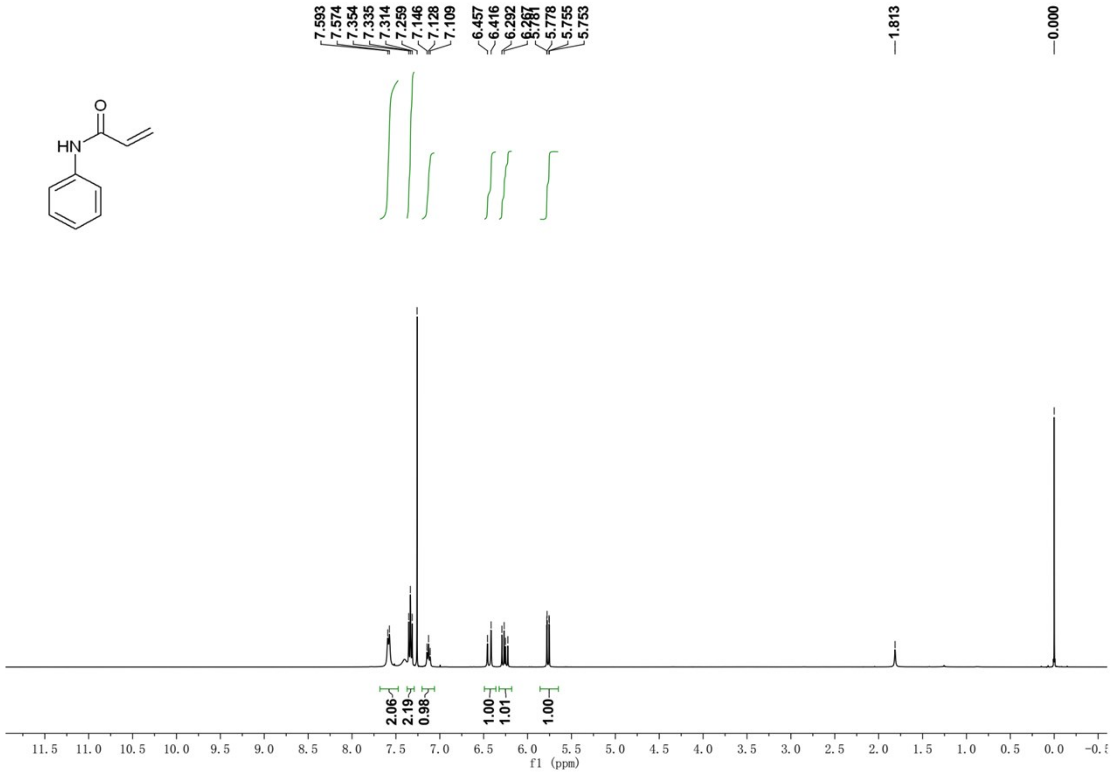

^{13}C NMR spectra of **9a**

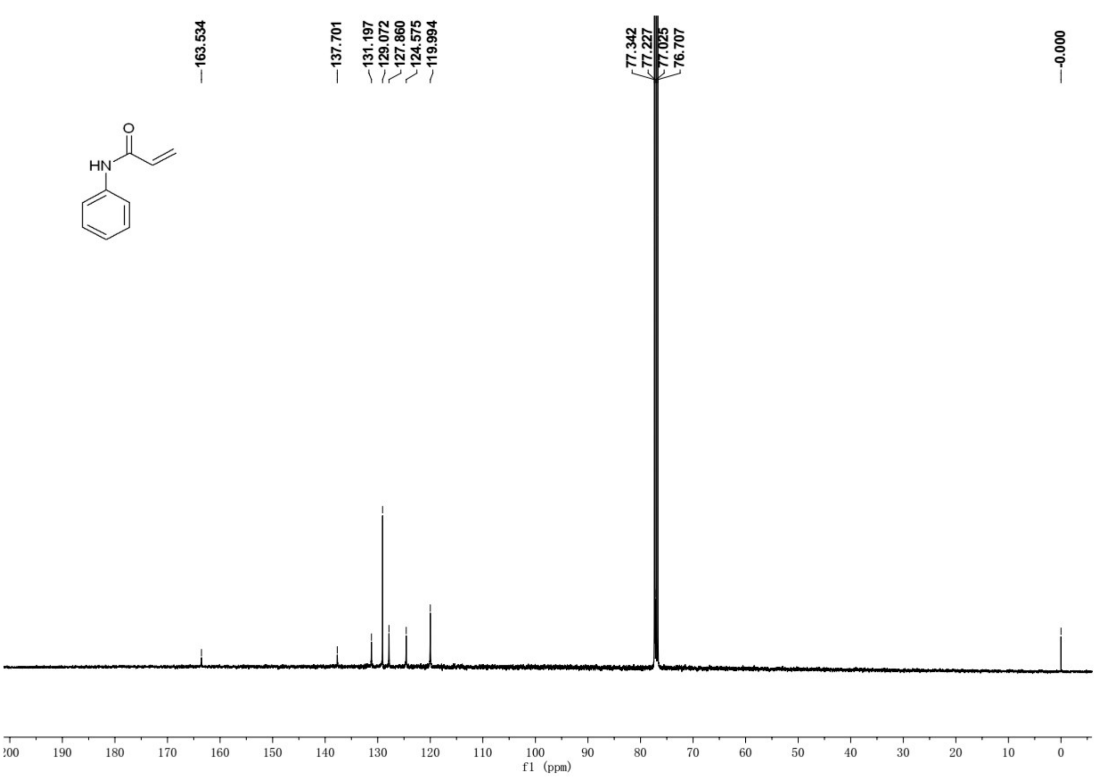

^{1}H NMR spectra of **10a**

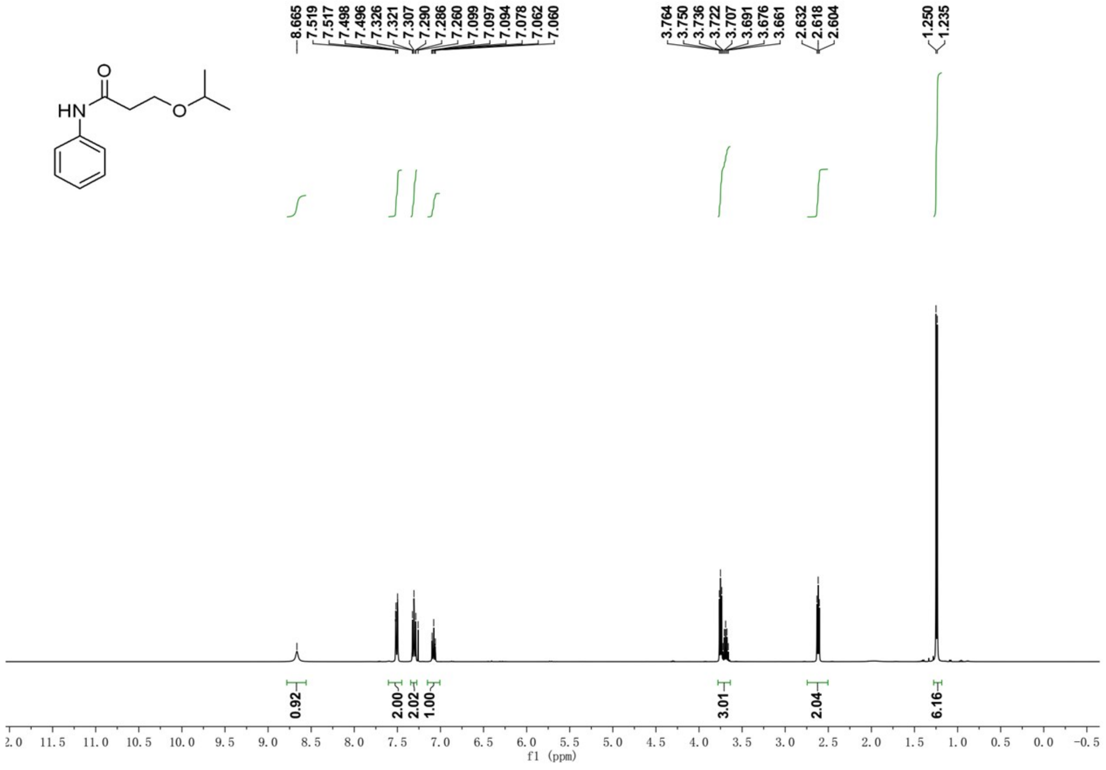

^{13}C NMR spectra of **10a**

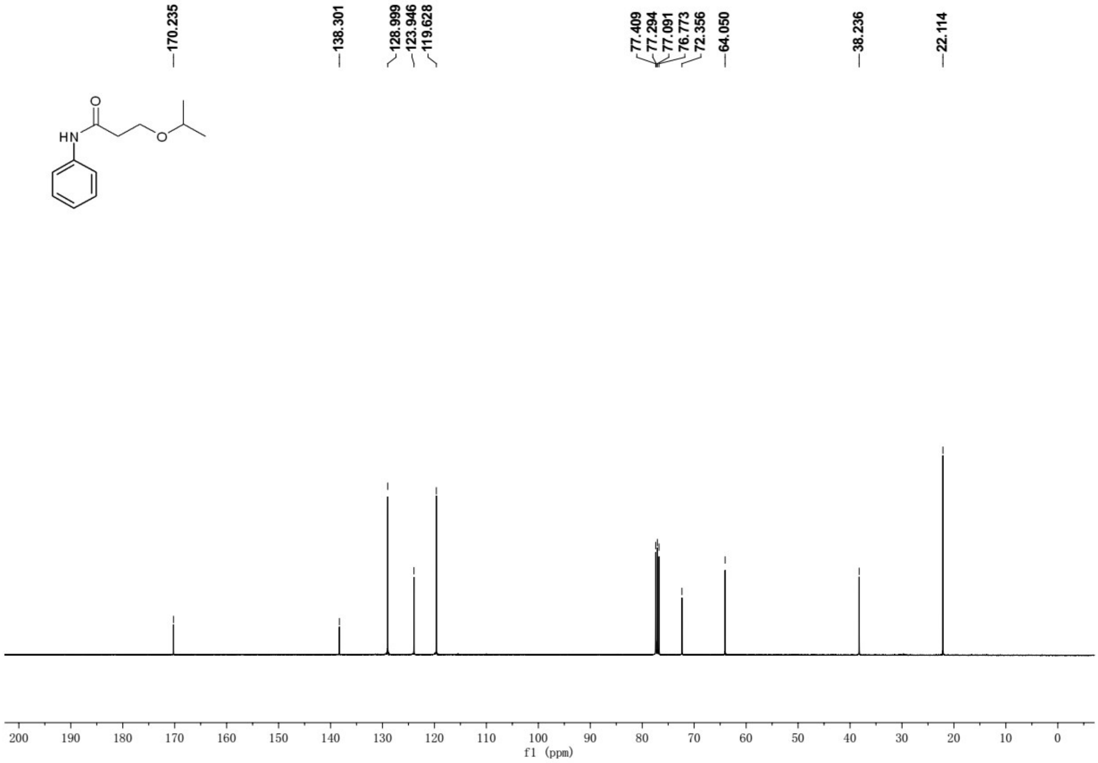

170.235
138.301
128.999
123.946
119.628
77.409
77.294
77.091
76.773
72.356
64.050
38.236
22.114
HN
O
O
200
190
180
170
160
150
140
130
120
110
100
90
80
70
60
50
40
30
20
10
0
f1 (ppm)
